# Intramolecular PCET of α‐Keto Acids: Synthesis of Trifluoromethyl Ketones via Ketyl Radicals

**DOI:** 10.1002/chem.202501613

**Published:** 2025-05-30

**Authors:** Rifat N. Nabi, Kimberly A. Jarquin, Anupam Karmakar, Kyle E. Brunner, Daniel K. Kim

**Affiliations:** ^1^ Department of Chemistry Temple University 1901 North 13th Street Philadelphia Pennsylvania 19122 USA

**Keywords:** dicarbofunctionalization, ketyl radical, lactone, photoredox, trifluoromethyl ketone

## Abstract

Due to favourable pharmacological properties, the introduction of the fluorine atom into small molecules has become increasingly more common over the past few decades. In particular, the trifluoromethyl ketone group is of great interest due to its role as a carboxylic acid bioisostere. Recently, these fragments have been accessed via acyl radical equivalents. Herein, we disclose an unconventional intramolecular proton‐coupled electron transfer activation of α‐keto acids by photoredox catalysis to generate ketyl radicals toward accessing functionalizable lactones. These lactones serve as synthons for modular one‐pot syntheses of trifluoromethyl ketones in a net dicarbofunctionalization of electron‐rich olefins.

## Graphical abstract

Decarboxylation of *α*‐keto acids have conventionally served as simple building blocks for the generation of nucleophilic acyl radicals. Herein, we demonstrate a distinct approach for this substrate class, showcasing the generation of ketyl radicals through a proton‐coupled electron transfer (PCET) mechanism. Coupled with aryl olefins, this approach allows for the generation of butyrolactones, a functional intermediate for the synthesis of trifluoromethyl ketones (TFMKs), in a net dicarbofunctionalization of aryl olefins.

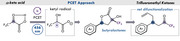

The incorporation of fluorine atoms within medicinal chemistry portfolios has been extensively established in drug discovery and development.^[^ ^1^, ^2^ ^]^ As a result, we are interested in incorporating fluorinated motifs using readily available building blocks. Specifically, trifluoromethyl ketones (TFMKs) have been shown to be strong surrogates for carboxylic acids, with significant impact on pharmacokinetic profiles within pKa, lipophilicity, and cell permeability.^[^ ^3^, ^4^ ^]^ Robust synthetic methods to access TFMKs via C─C bond forming reactions are well reported but often rely on electrophilic reagents such as trifluoroacetic anhydride (TFAA).^[^ ^5^ ^]^ A seminal report by Katayev and coworkers demonstrated that trifluoroacetylation can be achieved through reduction of TFAA, yielding the trifluoromethylacyl radical, which can subsequently couple with electron‐rich olefins.^[^ ^6^ ^]^ In a complementary approach toward electron‐deficient olefins, our group reported a masked trifluoromethylacyl radical equivalent in the form of a trifluoromethyl acetal which allowed for a polarity matched cross‐coupling event (Figure 1A).^[^ ^7^ ^]^

**Figure 1:**
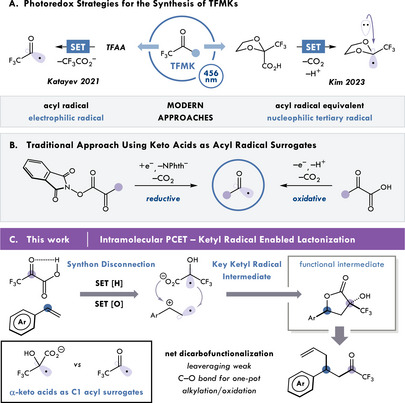
A) Photoredox methods for accessing trifluoromethyl ketones, B) conventional methods for α‐keto acid activation, and C) activation of *α*‐keto acids via PCET for the synthesis of butyrolactones and trifluoromethyl ketones.

To expand the synthetic methods of installing TFMKs, we envisioned taking trifluoropyruvic acid as a readily accessible C1 surrogate for acyl radicals. Among the diverse chemical building blocks used to access acyl radicals, *α*‐keto acids have conventionally served as a simple and convenient substrate class for radical activation.^[^ ^8^, ^9^ ^]^ Specifically, *α*‐keto acids can be activated by single electron transfer (SET), either oxidatively^[^ ^10^, ^11^ ^]^ from the native acid or reductively from the redox‐active ester,^[^ ^12^, ^13^ ^]^ to generate acyl radicals (Figure 1B). While direct generation of the trifluromethylacyl radical would allow for the desired coupling, these acyl radicals are known to be limited in stability as they are particularly susceptible to decarbonylation, making them difficult to handle. To that end, Katayev and co‐workers use concentration and pressure controls to overcome rapid decarbonylation of the acyl radical. We envisioned an alternative approach for activating *α*‐keto acids, whereby initial reduction of trifluoropyruvic acid would result in a stabilized ketyl radical, acting as a masked acyl radical.

It is well known that ketyl radicals can be formed in the presence of super stoichiometric amounts of reductants,^[^ ^14^, ^15^ ^]^ along with catalytic approaches leveraging modern electrochemical and photochemical modes of activation.^[^ ^16^, ^17^, ^18^, ^19^, ^20^, ^21^, ^22^, ^23^ ^]^ Using visible‐light photoredox catalysis, the Knowles group demonstrates the generation of ketyl radicals facilitated by an intermolecular proton‐coupled electron transfer (PCET) mechanism.^[^ ^24^ ^]^ However, a general strategy for the intramolecular activation of carbonyls for the formation of ketyl radicals has yet to be explored.^[^ ^16^ ^]^ We questioned whether *α*‐keto acids could serve as C1 surrogates for acyl radicals, under visible‐light photoredox catalysis, circumventing issues of direct decarboxylation of pyruvic acids.

Herein, we present an alternative activation of *α*‐keto acids through a PCET‐mediated transformation into a ketyl radical for the synthesis of butyrolactones, which are prevalent motifs in pharmaceutical^[^ ^25^ ^]^ and agricultural^[^ ^26^ ^]^ scaffolds. This transformation is achieved through a radical polar crossover (RPC) lactonization strategy.^[^ ^27^, ^28^, ^29^ ^]^ Furthermore, these α‐trifluoromethylbutyrolactones serve as viable synthetic intermediates for the synthesis of TFMKs in a net difunctionalization of aryl olefins. We commenced reaction optimization by testing various readily accessible *α*‐keto acids. Irradiation of electron‐deficient *α*‐keto acid **1** and diphenyl styrene, with 34 W Kessil 456 nm LED, in the presence of sufficiently low excited state reductant Ir(ppy)_3_ (E_1/2_ [Ir^IV^/^*^Ir^III^] = ‐1.73 V vs. SCE in MeCN),^[^ ^30^ ^]^ afforded high coupling yield (Table 1, entry 1, 86% yield). More oxidizing photocatalysts, such as [Ir[dF(CF3)ppy)_2_(dtbpy)]PF_6_ and 4CzIPN, gave diminished yields. Although EtOAc was found to be the optimal solvent for lactonization due to enhanced solubility of the keto acid, other solvents such as DMF and MeCN proceeded with moderate yields. Furthermore, control experiments revealed that the reaction was unsuccessful in delivering the desired product in the absence of photocatalyst and light (Table 1, entries 7 and 8). In comparison, *α*‐keto acid **2** and **3**, resulted in lower coupling yields (Table 1, entries 2 and 3). This is consistent with cyclic voltammetry data, as *α*‐keto acid **1** is easier to reduce (E_i_ [**1**/**1** ^•–^] = ‐1.01 V vs. SCE in MeCN) in comparison to electron‐donating *α*‐keto acids **2** (E_i_ [**2**/**2** ^•–^] = ‐1.11 V versus SCE in MeCN) and **3** (E_i_ [**3**/**3** ^•–^] = ‐1.17 V vs, SCE in MeCN). Although trifluoropyruvic ester **4** has a lower reduction potential (E_i_ [**4**/**4** ^•–^] = ‐0.79 V vs. SCE in MeCN), coupling was not observed under current reaction conditions (Table 1, entry 4). We suspect the lack of reactivity has to do with a fast back electron transfer (BET) between the resulting ketyl radical anion **4** ^•–^ and the oxidized form of the photocatalyst over productive C─C bond formation. In agreement with the literature, this observation suggests the coupled proton and electron transfer increases the persistency of the ketyl radical that can efficiently react with styrene.^[^ ^31^, ^32^ ^]^ To further probe this hypothesis, we attempted coupling of **4**, in the presence of formic acid, an intermolecular proton source, to reveal radical addition product **4a** in 56% yield as a 1.5:1 mixture of diastereomers (Table 1, entry 5). An essential control reaction shows the use of formate does not lead to product **4a** suggesting the proton is essential for persistent radical formation (Table 1, entry 6). Altogether, these results suggest that keto acids are unique ketyl radical precursors that are enabled by an intramolecular PCET to increase radical persistency by slowing BET.

**Table 1:** Optimization of *α*‐keto acids and reaction conditions. See Supporting Information for reaction details.

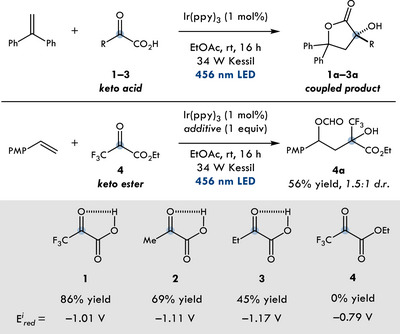			
Entry	Conditions	Product	Yield [%]
1	**1**	**1a**	86
2	**2** instead of **1**	**2a**	69
3	**3** instead of **1**	**3a**	45
4	**4** instead of **1**	**4a**	0
5	**4** + HCO_2_H	**4a**	56
6	**4** + HCO_2_NH_4_	**4a**	0
7	**1**, no photocatalyst	**1a**	0
8	**1**, no light	**1a**	0

With optimized conditions in hand, we evaluated the scope with trifluoropyruvic acid **1** as an intramolecular ketyl radical precursor for the synthesis of highly functionalized butyrolactones. The model substrate, 4‐methoxystyrene, coupled to afford the desired product (**5**) in a 96% isolated yield and 2.8:1 *d.r*. (Figure 2). Notably, 4‐methoxylactone **5** was prepared on a gram scale without any significant impact on yield (93%). Additionally, trimethoxystyrene and ortho‐methoxystyrene coupled to give the desired product (**6** and **7**) in comparable yields (92–94% yields). *t*‐butyl styrene also gave the desired product (**12**) in 85% yield. Ester and halogen containing olefins were amenable to our methodology, yielding the corresponding lactones (**8**–**11**) in moderate yields (55–70% yield). X‐ray crystallography structure of **9** revealed the relative stereochemistry of the major diastereomer. Furthermore, we were delighted that this method can be extended to cyclic 1,2‐disubstituted alkenes. Herein we show that 7‐methoxy‐dihydronapthalene was well tolerated to the fused lactone product **13** in 69% yield. In comparison, RPC of alkyl radicals has presented a significant challenge within the field.^[^ ^31^, ^32^, ^33^ ^]^ Attempts to achieve lactonization of alkyl olefins with trifluoropyruvic acid were unsuccessful (see Supporting Information for details), suggesting insufficient reactivity of the key ketyl radical intermediate.

**Figure 2:**
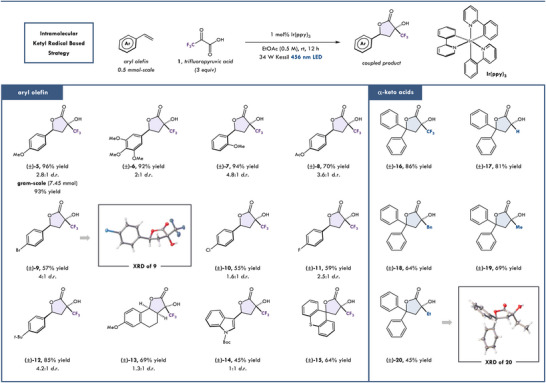
Evaluation of the photoredox‐catalyzed lactonization reaction. See the Supporting Information for reaction details.

In order to highlight the broad application of our protocol, we envisioned using differentiated *α*‐keto acids for the generation of ketyl radicals in the coupling to electron‐rich olefins. As a reference, trifluoropyruvic acid with diphenyl styrene gave the desired product (**16**) in 86% yield. Coupling of glyoxylic acid with diphenyl styrene gave the desired product **17** in 81% yield. Switching to more electron‐donating keto acids, resulted in coupled products **18**–**20**, in lower yields of 45–64% yields, showcasing the preferential polarity matching of the trifluoromethyl ketyl radical (**IV**).

In our proposed catalytic cycle, highlighted in Figure 3A, excited state Ir^III^ **II** reduces trifluoropyruvic acid **1** through a PCET process, generating a trifluoromethyl ketyl radical **IV**. Addition into aryl olefin and SET by oxidant Ir^IV^ **III** (E_1/2_ [Ir^IV^/Ir^III^] = +0.77 V vs. SCE in MeCN) results in the subsequent RPC^[^ ^31^, ^34^ ^]^ of **V**, turning over the catalytic cycle. Lastly, nucleophilic attack of benzylic carbocation **VI** furnishes highly functionalized butyrolactone **VII**. To validate our hypothesis, we first needed to prove that trifluoropyruvic acid quenches excited state photocatalyst. Stern–Volmer analysis revealed that trifluoropyruvic acid **1** (*k*_q_ = 7.58×10^7^ M^−1^s^−1^) quenches excited state Ir(ppy)_3_ faster than 4‐ methoxystyrene (*k*_q_ = 2.19×10^6^ M^−1^s^−1^; Figure 3B). Second, if an intramolecular PCET mechanism is operative then we *should* observe a primary kinetic isotope effect (KIE) with deuterated trifluoropyruvic acid. Stern–Volmer analysis of the deuterated and protonated trifluoropyruvic acid resulted in a measured primary KIE (*k*_H_/*k*_D_ = 1.52). This agrees with other literature precedence for ketyl radicals generated by PCET mechanisms (see Supporting Information for further details).^[^ ^24^ ^]^ In addition, following methods developed by Burés,^[^ ^35^ ^]^ visual time normalization analysis was used to conduct initial kinetics in order to reveal that the reaction is first order in trifluoropyruvic acid and zeroth order in styrene (see Supporting Information for further details). With this information in hand, we propose that PCET is the turnover limiting step of the proposed catalytic cycle.

**Figure 3:**
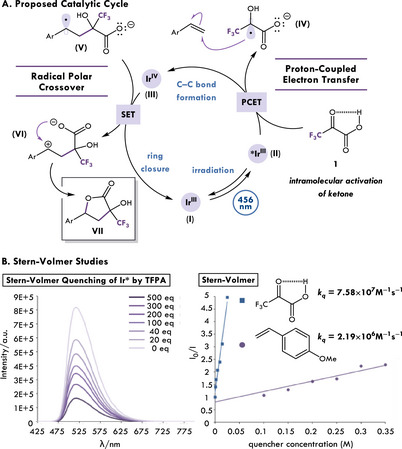
A) Proposed catalytic cycle and B) Stern–Volmer quenching experiments.

In order to demonstrate the value of CF_3_‐butyrolactones as synthetic intermediates, direct conversion to TFMKs has been realized, showcasing the ability for *α*‐keto acids to act as acyl radical surrogates in C1 reagent design, in a one‐pot net dicarbofunctionalization reaction. Taking advantage of the labile benzylic C─O bond, net dicarbofunctionalization allows for the synthesis of complex building blocks from readily available starting materials.^[^ ^36^ ^]^ It has been previously demonstrated that benzylic *O*‐heterocycles, such as furans and lactones, can be ring opened under mild Lewis acidic conditions, in order to furnish the respective C─C bond formation.^[^ ^37^ ^]^ Furthermore, the resulting hydroxy‐CF_3_ acid can undergo stepwise oxidative decarboxylation in order to yield the desired TFMK via an interrupted net dicarbofunctionalization of electron rich styrenes. Using these conditions as precedent, we have optimized and showcased a one‐pot modular synthesis of TFMKs through a dual FeCl_3_ mediated ring opening and functionalization with allyl TMS and subsequent oxidative decarboxylation under aerobic conditions^[^ ^38^, ^39^ ^]^ (Figure 4). We were delighted in finding that one‐pot ring opening and subsequent oxidative decarboxylation of electron‐rich methoxy styrenes furnished the respective TFMKs (**21**–**23**) in high yields (94–97%). Functional handles such as acetoxy lactone gave the desired product (**23**) in 62% yield. Lactones bearing halogenated aryl substituents appeared to be challenging substrates for functionalization under the current conditions and required stronger Lewis acids^[^ ^40^ ^]^ such as Sc(OTf)_3_ to give the respective TFMKs **25**–**27** in 45–52% yields (see Supporting Information for further details). In addition, heterocycles such as thioxanthane lactone, gave the functionalized TFMK **29** in 96% yield. Furthermore, ring opening of fused lactone **13** gave the desired TFMK **30** in 85% yield as a mixture of diastereomers (7:1 d.r.).

**Figure 4:**
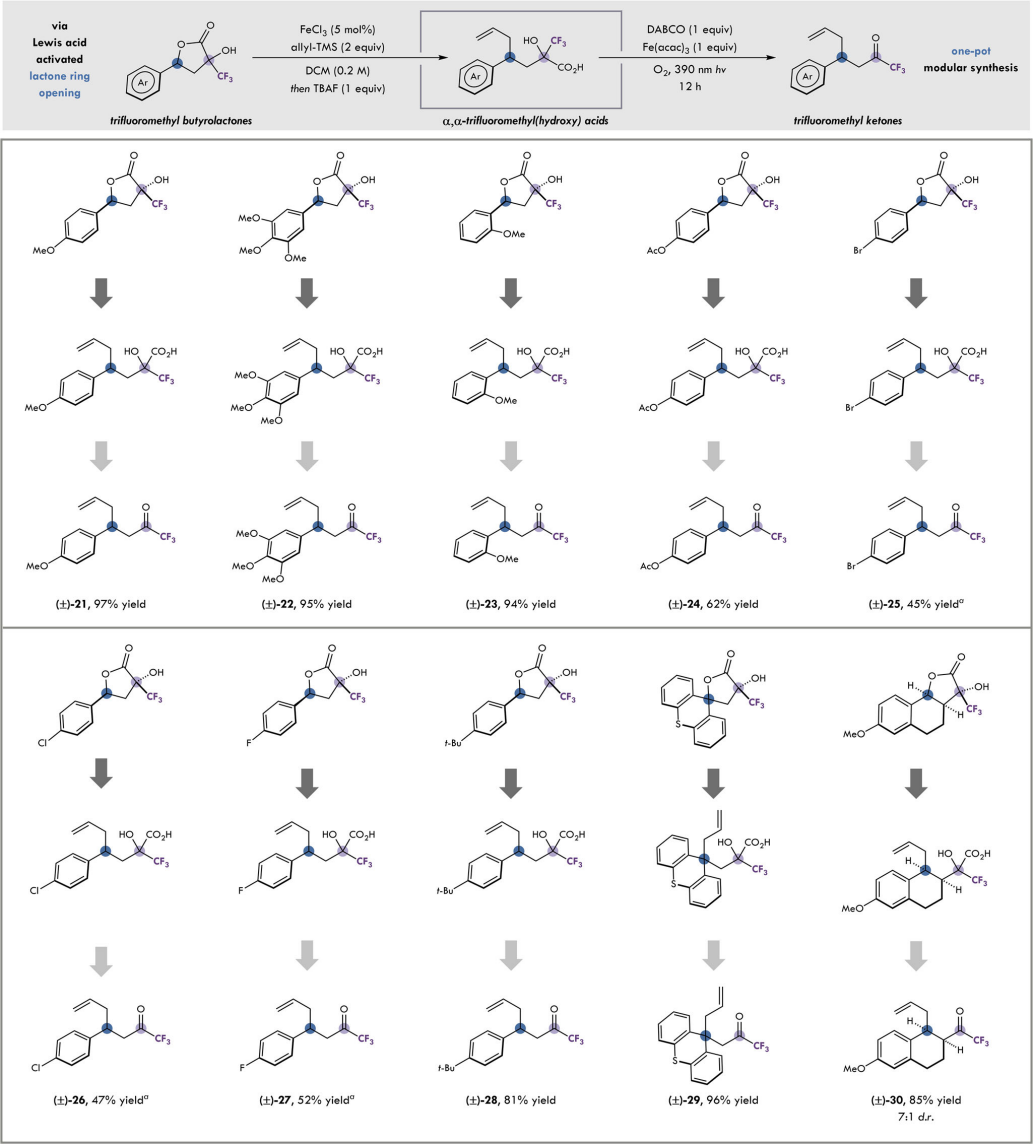
Evaluation of one‐pot synthesis of trifluoromethyl ketones. See Supporting Information for reaction details. *^a^* Sc(OTf)_3_ was used as a Lewis acid.

In conclusion, we have demonstrated a photoredox catalyzed method for the generation of ketyl radicals from intramolecular hydrogen bonding of keto acids. This PCET mechanism is corroborated by Stern–Volmer quenching studies including the use of kinetic isotope effects. This method enables rapid access to highly functionalized lactones. Furthermore, we can take advantage of the weak C─O bond and forge a new C─C bond in an overall net difunctionalization of aryl olefins, providing a modular synthesis of TFMKs.

## Conflict of Interests

The authors declare no conflict of interest.

## Supporting information



Supporting Information

## Data Availability

The data that support the findings of this study are available in the supplementary material of this article.
