# Supplementary material for: Intramolecular PCET of α‐Keto Acids: Synthesis of Trifluoromethyl Ketones via Ketyl Radicals
Source: Chemistry. 2025 May 30;31(37):e202501613. doi: 10.1002/chem.202501613 (PMC12223336; doi:10.1002/chem.202501613)
Supplement: Supplementary file 1 — Supporting Information [file CHEM-31-e202501613-s001.docx]

**Supporting Information**

**Intramolecular PCET of α-Keto Acids:
Synthesis of Trifluoromethyl Ketones via Ketyl Radicals**

*Rifat N. Nabi, Kimberly A. Jarquin, Anupam Karmakar, Kyle E. Brunner, and Daniel K. Kim**

*Department of Chemistry, Temple University 1901 North 13th Street, Philadelphia, Pennsylvania, 19122, United States**corresponding author. Email: danielkim@temple.edu

**Table of Contents**

[**General Information** 2](#_Toc191988823)

[**Cyclic Voltammetry Data** $\boldsymbol{\alpha}$**-keto acids** 6](#_Toc191988824)

[**Stern-Volmer Quenching Studies** 9](#_Toc191988825)

[**Kinetic Order Determination** 14](#_Toc191988826)

[**General Procedure for the Synthesis of Starting Materials and Characterization** 17](#_Toc191988827)

[**General Procedure for PCET Lactonization and Characterization** 23](#_Toc191988828)

[**General Procedure for One-Pot Trifluoromethyl Ketone Synthesis** 40](#_Toc191988829)

[**X-Ray Diffraction Data** 52](#_Toc191988830)

[**References** 58](#_Toc191988831)

[**1H, 13C NMR and 19F NMR spectra of all compounds** 59](#_Toc191988832)

# **General Information**

Commercially available reagents were used without additional purification, unless otherwise indicated. Reaction vials (8 mL) were purchased from Fischer Scientific, oven dried overnight, and cooled to room temperature prior to use. Solvents were purified using Pure Process Technology 5-Solvent Purification System (DMF, MeCN, THF, DCM, Et_2_O). Anhydrous, reagent grade, ethyl acetate was purchased and used as received from Sigma Aldrich. Photocatalysts were purchased and used as received, Ir(ppy)3 (Combi-Blocks, PN: QG-1453). Unless otherwise noted, reactions were performed with rigorous exclusion of air and moisture. Thin layer chromatography was carried out using TLC Silica gel 60 F254 plates (Sigma Aldrich, PN: 1.05715.0001). For flash column chromatography, bulk silica gel (230–400 mesh) was used (Natland International Corporation, PN: 80001-20). Purification was carried out using ACS grade solvents.

Nuclear magnetic resonance spectra (1H NMR, 13C NMR) were recorded on a Bruker Unity 400 MHz, 500 MHz, or 600 MHz spectrometers in CDCl3, DMSO-d6, or MeOD-d4 solutions. 1H NMR Data are reported in terms of chemical shift (δ, ppm), multiplicity (s = singlet, d = doublet, t = triplet, q = quartet, m = multiplet, br = broad), coupling constant (Hz), and integration. Chemical shifts are reported as parts per million (ppm) standardized to the resulting spectra were internally referenced to the residual proteo-solvent signals (7.26 ppm for CDCl3; 2.50 ppm for DMSO-d6; 4.78 for MeOD-d4). 13C NMR data are reported in terms of chemical shift (δ, ppm), multiplicity (q = quartet), and coupling constant (Hz) when there is coupling.

High Resolution Mass Spectroscopy (HRMS) Accurate masses for derivatized products were conducted on an Agilent 6520 Accurate-Mass Q-TOF LC/MS. Samples were taken up in a suitable solvent (MeCN) for analysis. Accurate mass measurement (AMM) analyses were conducted on either a Waters GCT Premier, time-of-flight, GCMS with electron ionization (EI-TOF), or an LCT Premier XE, time-of-flight, LCMS with electrospray ionization (ESI-TOF). The signals were mass measured against an internal lock mass reference of perfluorotributylamine (PFTBA) for EI-GCMS and leucine enkephalin for ESI-LCMS, positive and negative ion modes. Waters software calibrates the instruments and reports measurements, by use of neutral atomic masses. The mass of the electron was not included.

Photochemical reactions were carried out using Kessil PR160L 456 nm at 100% intensity. Reaction vials are set one cm away from the light source using Kessil’s PR160 Rig with Fan Kit.

Cyclic voltammetry (CV) experiments were performed using a CHI 750E electrochemical analyzer/workstation with a three-electrode setup. The working electrode was a glassy carbon electrode (CH Instruments CHI 104), the counter electrode was a platinum wire counter electrode (CH Instruments CHI115), and a Ag/Ag^+^ reference electrode (CH Instruments CHI112) with 0.01 M Ag/AgNO₃ reference solution. Measurements were conducted in acetonitrile (MeCN) at a scan rate of 0.1 V/s with 0.1 M tetrabutylammonium hexafluorophosphate (TBAPF₆) as the supporting electrolyte.

All experiments were carried out under a constant flow of nitrogen to avoid oxygen interference. Potentials are reported relative to the saturated calomel electrode (SCE), with the Ag⁺/Ag couple measured at +343 mV vs. SCE in MeCN.^1^ Reduction potentials are typically determined from the midpoint between the anodic and cathodic peak potentials. However, since our system involves irreversible redox processes, we instead use the inflection point potential (Eᵢ) of the first observable wave in the forward scan. In these cases, Eᵢ values are calculated following the method outlined by Vullev.^2^

D8 VENTURE (Mo)

Single-crystal X-ray crystallographic data were obtained on a Bruker D8 Venture Duo diffractometer and a PHOTON III CMOS detector with an Oxford Cryostream 700 low-temperature device. The radiation was from a sealed-tube molybdenum Kα source with an IμS DIAMOND microfocus optic. Crystals were mounted on a MiTeGen loop with paratone-N oil and collected at 100K in ω/φ scansets. Integration was performed using SAINT, and data were reduced and absorption-corrected using SADABS (Bruker, 2008). Space group determination was performed using XPREP (Sheldrick, 2008), and the structure was solved using intrinsic phasing with SHELXT (Sheldrick, 2015b) The structural models were refined using the least-squares approach with the ShelX package (Sheldrick, 2015a) and Olex2 as a GUI (Dolomanov et al., 2009).

D8 VENTURE (Cu)

Single-crystal X-ray crystallographic data were obtained on a Bruker D8 Venture Duo diffractometer and a PHOTON III CMOS detector with an Oxford Cryostream 700 low-temperature device. The radiation was from a sealed-tube copper Kα source with an IμS 3.0 microfocus optic. Crystals were mounted on a MiTeGen loop with paratone-N oil and collected at 100K in ω/φ scansets. Integration was performed using SAINT, and data were reduced and absorption-corrected using SADABS (Bruker, 2008). Space group determination was performed using XPREP (Sheldrick, 2008), and the structure was solved using intrinsic phasing with SHELXT (Sheldrick, 2015b) The structural models were refined using the least-squares approach with the ShelX package (Sheldrick, 2015a) and Olex2 as a GUI (Dolomanov et al., 2009).

Citations:

Bruker (2008). COSMO, SAINT, and XPREP. Bruker AXS Inc, Madison, Wisconsin, USA.

Sheldrick, G. M. (2008). XPREP Version 2008/2, Bruker AXS Inc, Madison, Wisconsin, USA.

Sheldrick, G. M. (2015b). Acta Cryst. A71, 3–8.

Sheldrick, G. M. (2015a). Acta Cryst. C71, 3–8.

Dolomanov, O. V., Bourhis, L. J., Gildea, R. J., Howard, J. A. K. & Puschmann, H. (2009). J. Appl. Cryst. 42, 339–341.

# **Reaction Optimization and Control Experiments**

**Procedure for Optimization Studies**

To an oven dried 8-mL vial with a stir bar, the respective photocatalysts, keto acid, styrene, and solvent (0.4 mL, 0.5 M) were added. The vial was capped and degassed with bubbling nitrogen stream for 15 minutes. The liquid alkenes (0.2 mmol, 1 equiv) are then added and the headspace is purged for an additional 1 minute. The vial is then sealed with parafilm and irradiated using 34 W Kessil PR160L 456 nm (1 cm away, with cooling fan to keep the reaction at room temperature) for 16 hours. Trifluorotoluene (internal standard, 0.2 mmol) was added then the reaction mixture was analyzed by ^19^F NMR. The vial was opened, diluted with DCM, and reduced under pressure. The crude material was purified by flash column chromatography.

**Figure S1.** Control Reactions

*Trifluoropyruvic acid was most soluble in ethyl acetate, consistent with the observed yield*

**Figure S2.** Solvent Evaluation

**Figure S3.** Stoichiometry Evaluation

*(Ir^IV^/*Ir^III^ = −1.73 V vs. SCE)*

**Figure S4.** Photocatalyst Evaluation

**Figure S5.** Time Evaluation

# **Unsuccessful Alkyl Olefins**

**Figure S6.** Norbornene Solvent Evaluation

**Figure S7.** Norbornene Catalyst Evaluation

**Figure S8.** 2-methyl-4-phenyl-1-butene Solvent Evaluation

**Figure S9.** 2-methyl-4-phenyl-1-butene Catalyst Evaluation

# **Cyclic Voltammetry Data** $\boldsymbol{\alpha}$**-keto acids**

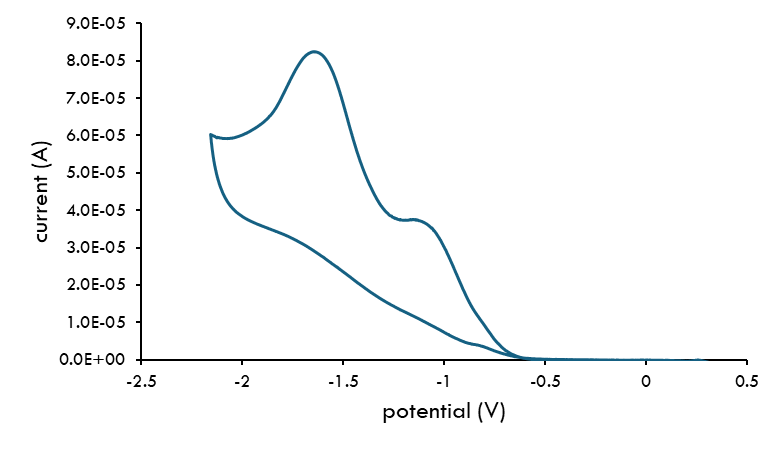


**Figure S10.** CV of (**1**) in MeCN (0.1 M NBu_4_PF_6_), 0.1 V/s scan rate


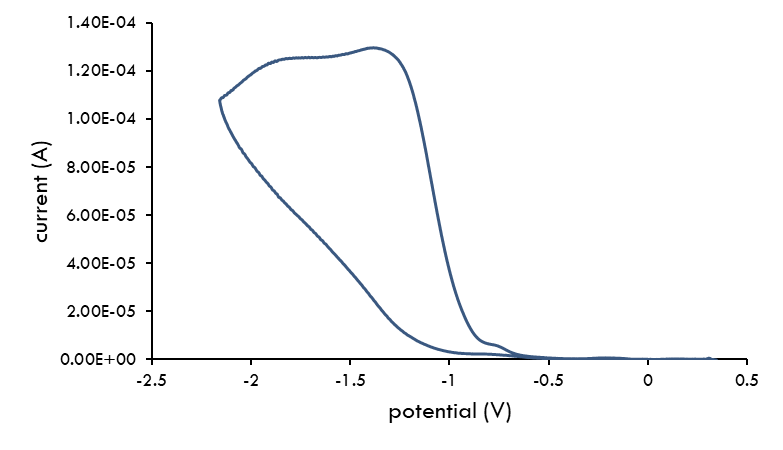


**Figure S11.** CV of (**2**) in MeCN (0.1 M NBu_4_PF_6_), 0.1 V/s scan rate


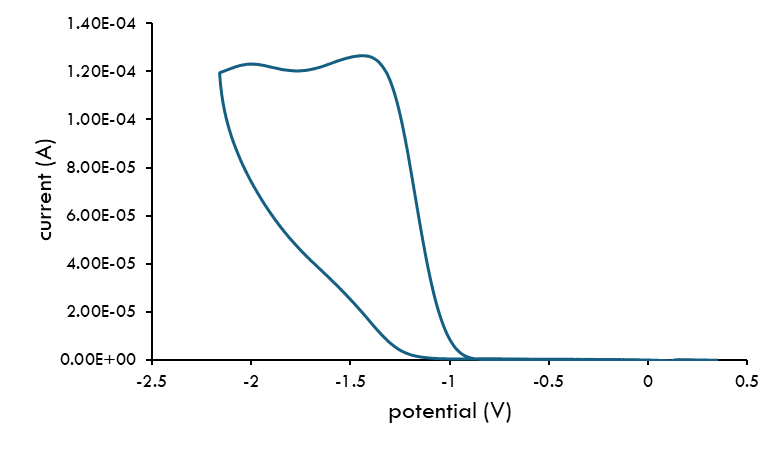


**Figure S12.** CV of (**3**) in MeCN (0.1 M NBu_4_PF_6_), 0.1 V/s scan rate


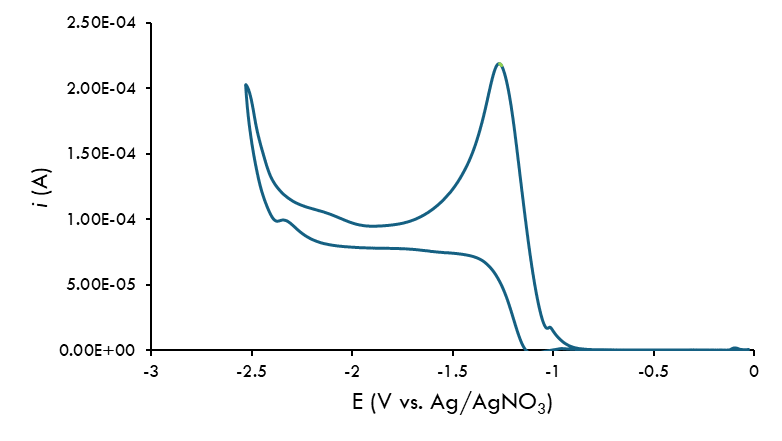


**Figure S13.** CV of (**4**) in MeCN (0.1 M NBu_4_PF_6_), 0.1 V/s scan rate.


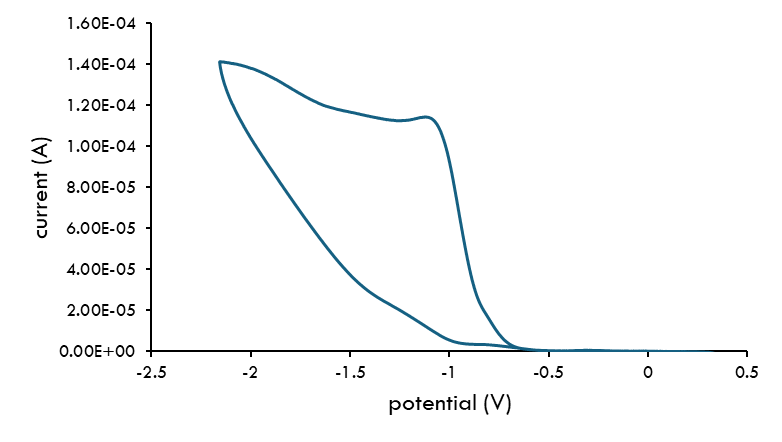


**Figure S14.** CV of (**4**) in MeCN (0.1 M NBu_4_PF_6_), 0.1 V/s scan rate

# **Stern-Volmer Quenching Studies**

$$\frac{I_{0}}{I}=1+k_{q}\tau_{0}[Q]$$

Stern–Volmer quenching studies were conducted in order to gain a deeper understanding of the photocatalyst excited state.

$I_{0}$ represents the fluorescence intensity of the photocatalyst in the absence of a quencher, while $I$ is the fluorescence intensity in the presence of a known quencher concentration, determined from the integrated area under the emission curve. The term $k_{q}$ denotes the quenching rate constant, and $\tau_{0}$ represents the excited-state lifetime of the photocatalyst, which has been reported as $1.90\times{10}^{-6} s$for Ir(ppy)_3_ in acetonitrile at 25°C.^3^

**Preparations of stock solutions:**

All preparation of solutions were done inside of a glovebox with oxygen and water free solvent free in volumetric flasks. Serial dilutions were done in order to obtain all concentrations.

A stock solution of Ir(ppy)_3_ was prepared by dissolving 3.27 mg Ir(ppy)_3_ (4.99 µmol, 499 µM) in 100 mL of dry acetonitrile.

A stock solution of 4-methoxystyrene by dissolving 559.8 µL (561 mg, 3.50 mmol, 350 mM) in 10 mL of dry acetonitrile.

A stock solution of ethyl trifluoropyruvate (ETFP) was prepared by dissolving 33.1 µL (42.5 mg, 250 µmol, 25 mM) in 10 mL of dry acetonitrile.

A stock solution of trifluoropyruvic acid (TFPA) was prepared by dissolving 35.5 mg TFPA (250 µmol, 25 mM) in 10 mL of dry acetonitrile.

A stock solution of deuterated trifluoropyruvic acid (*d*-TFPA) was prepared by dissolving 35.8 mg *d*-TFPA (250 µmol, 25 mM) in 10 mL of dry acetonitrile.

Each sample was prepared in the glovebox with minimal light exposure in quartz cuvettes (3.5 mL, 10 mm × 10 mm, PTFE screw cap). Each cuvette was filled with 0.5 mL of photocatalyst stock solution, 0.5 mL of reagent stock solution, and 0.5 mL of dry acetonitrile. After capping the cuvette, the cap was parafilmed, and the cuvette was wrapped in aluminum foil to avoid light exposure. Data was collected for each sample immediately after preparation. Parameters for spectra were set accordingly, excitation at 430 nm, emission range from 460 nm to 800 nm, 1 nm step, excitation slit of 1 nm and emission slit of 2 nm. Emission spectrum of 1.5 mL of dry acetonitrile was taken first in order to serve as our blank. To ensure consistency, a fresh batch of iridium stock solution was prepared before testing each substrate, along with a new emission spectrum for each batch.

In order to obtain the *k_q_* value for each, the slope of the line, *K_SV_*, was divided by $\tau_{0}=1.90\times{10}^{-6} s.$


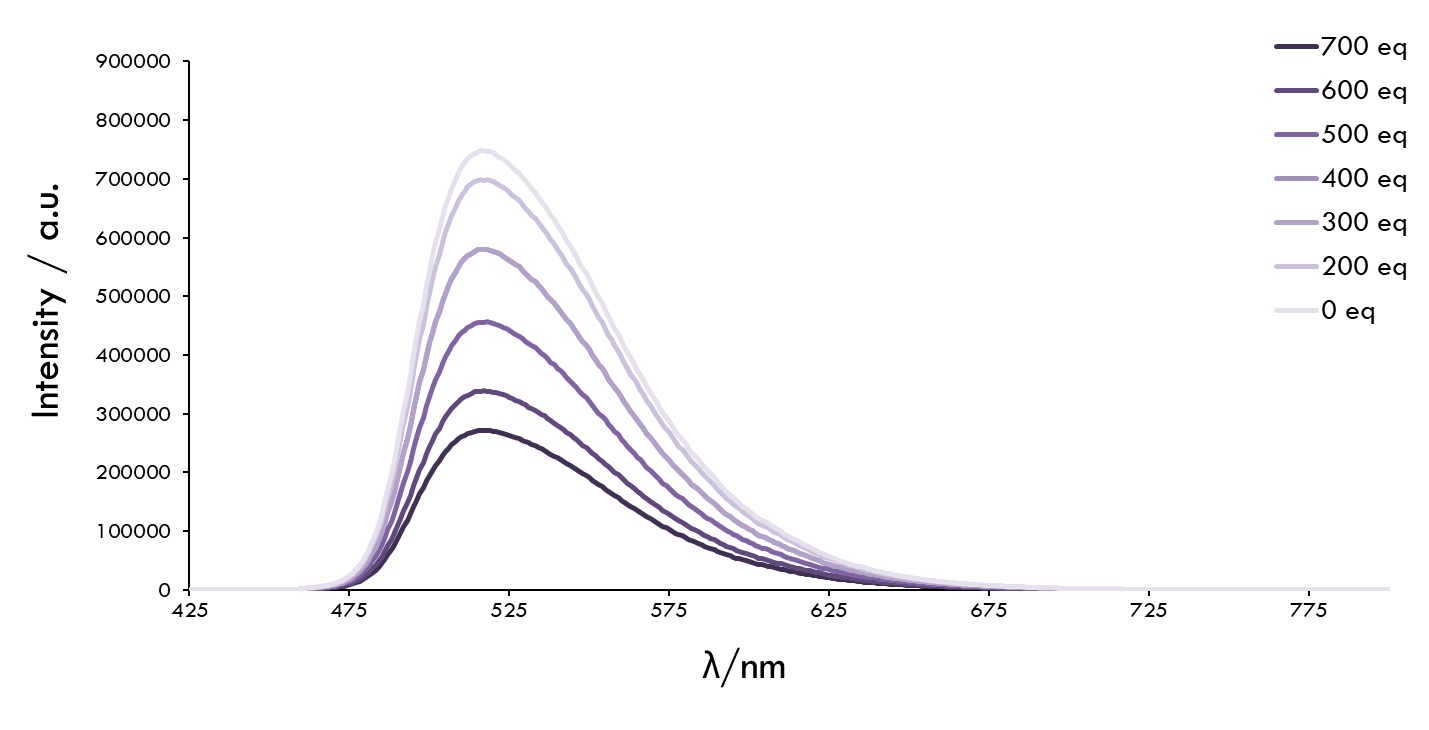


**Figure S15.** Emission quenching of Ir(ppy)_3_ with 4-methoxy styrene in acetonitrile


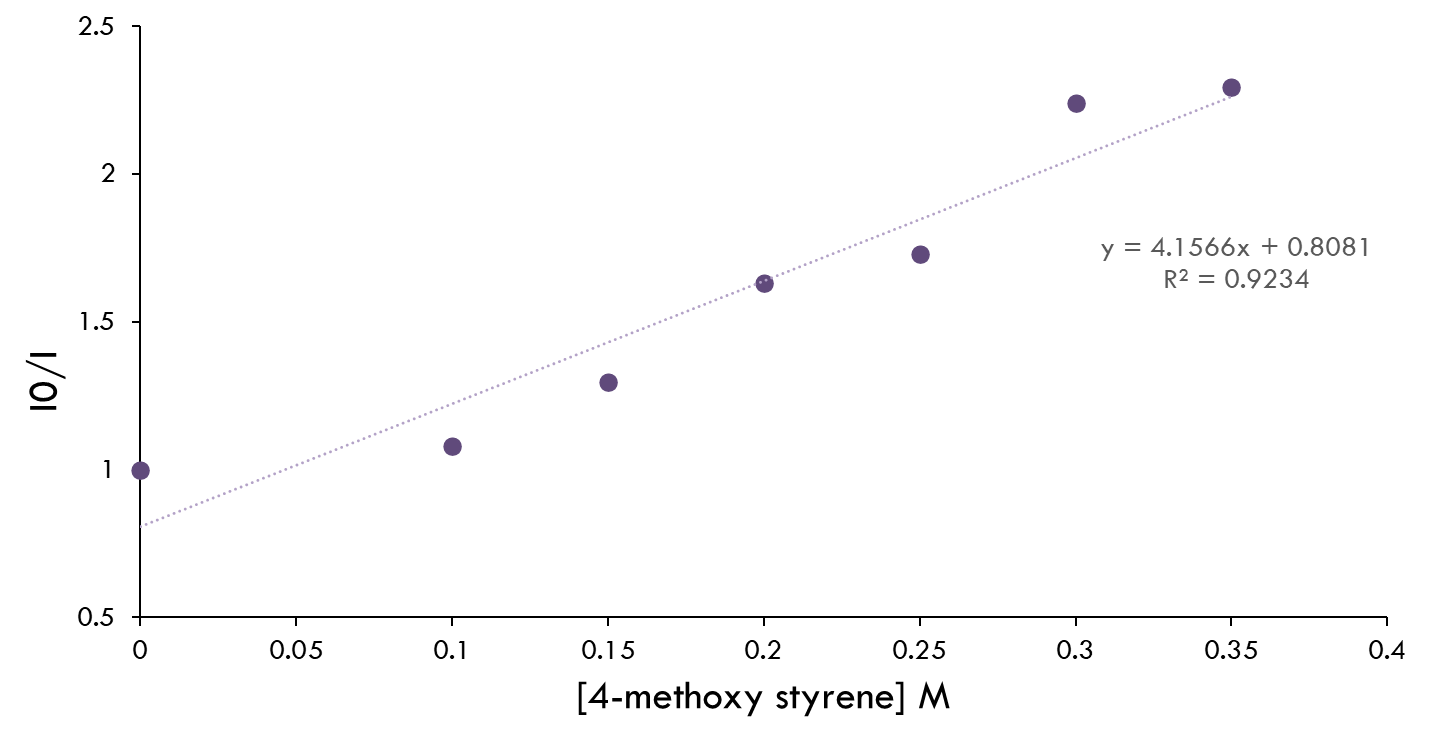


**Figure S16.** Stern–Volmer plot of Ir(ppy)_3_ with 4-methoxy styrene in acetonitrile. Calculated quenching constant of $k_{q}=2.19\times{10}^{6}M^{-1}s^{-1}$


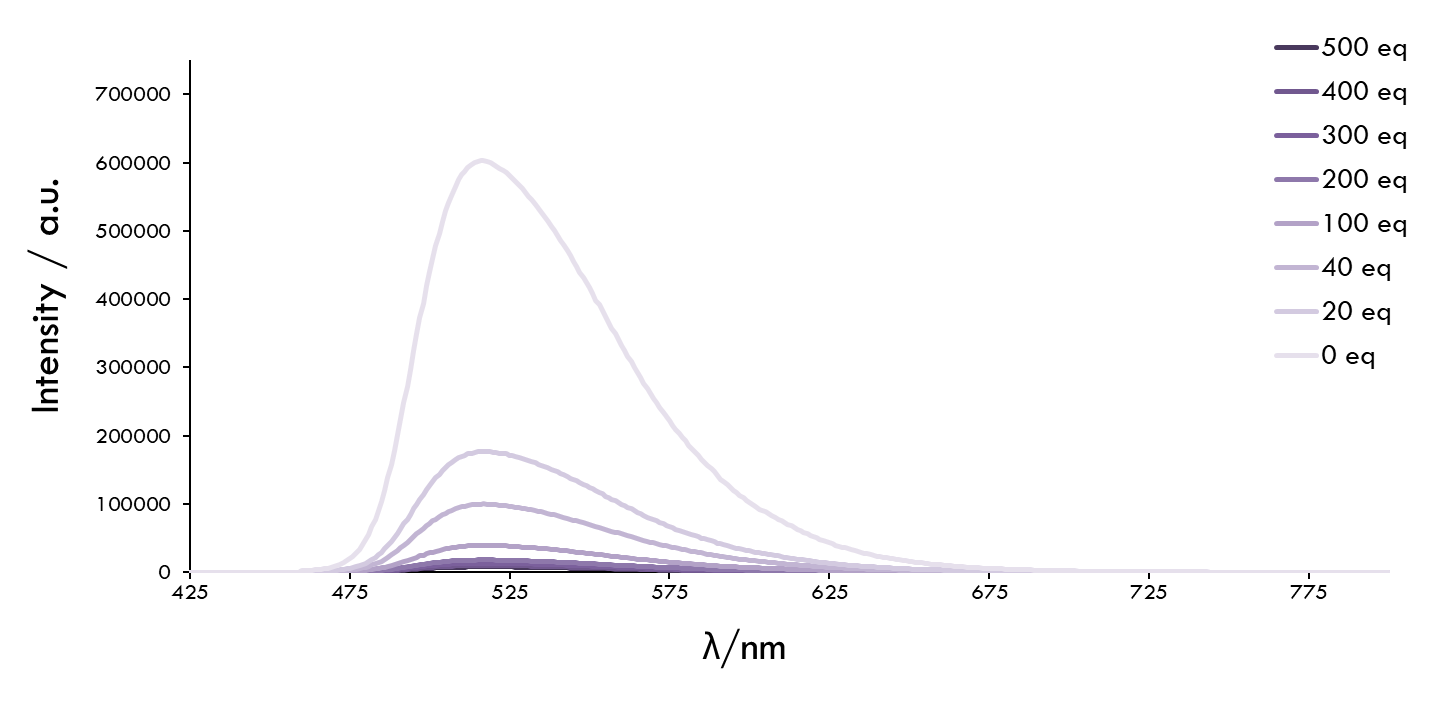


**Figure S17.** Emission quenching of Ir(ppy)_3_ with ethyl trifluoropyruvate (ETFP) in acetonitrile


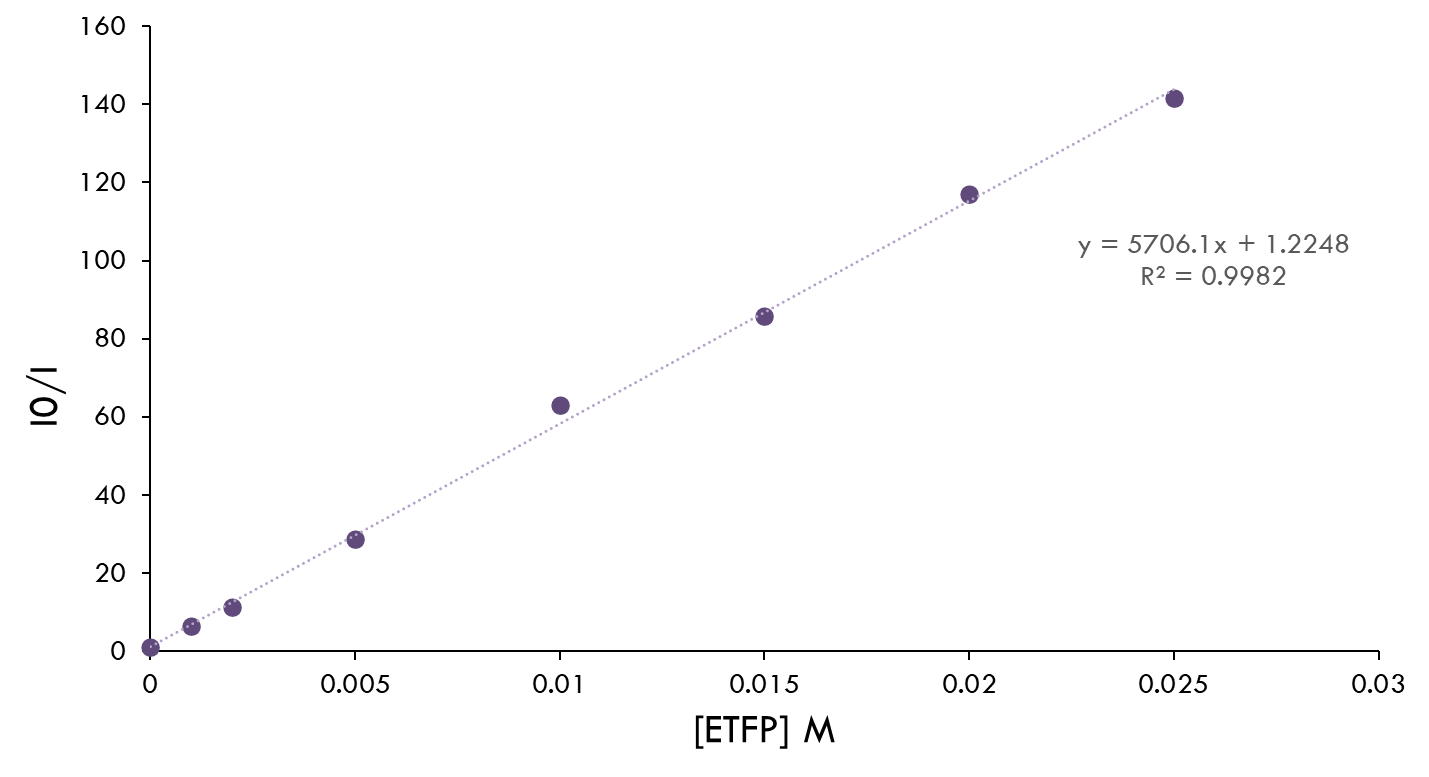


**Figure S18.** Stern–Volmer plot of Ir(ppy)_3_ with ethyl trifluoropyruvate (ETFP) in acetonitrile. Calculated quenching constant of $k_{q}=3.00\times{10}^{9} M^{-1}s^{-1}$


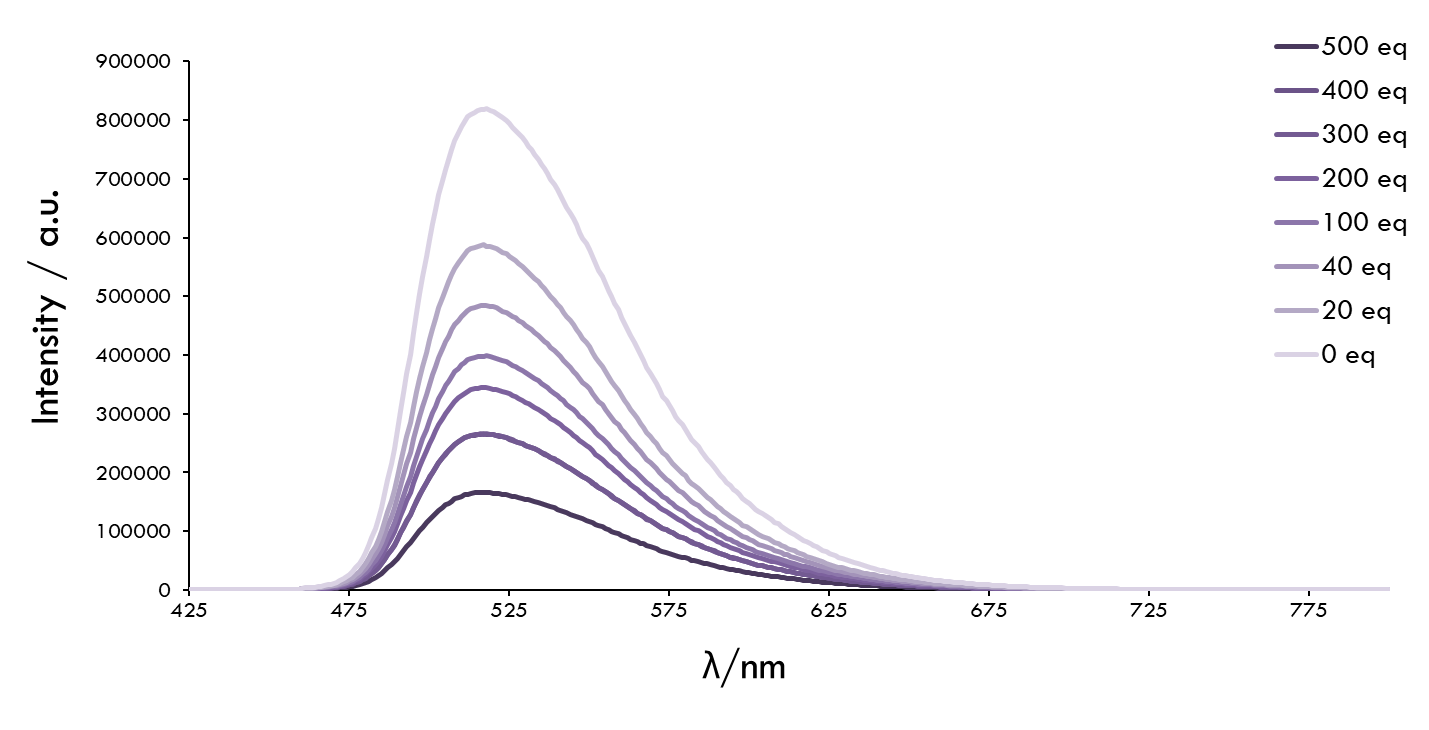


**Figure S19.** Emission quenching of Ir(ppy)_3_ with trifluoropyruvic acid (TFPA) in acetonitrile


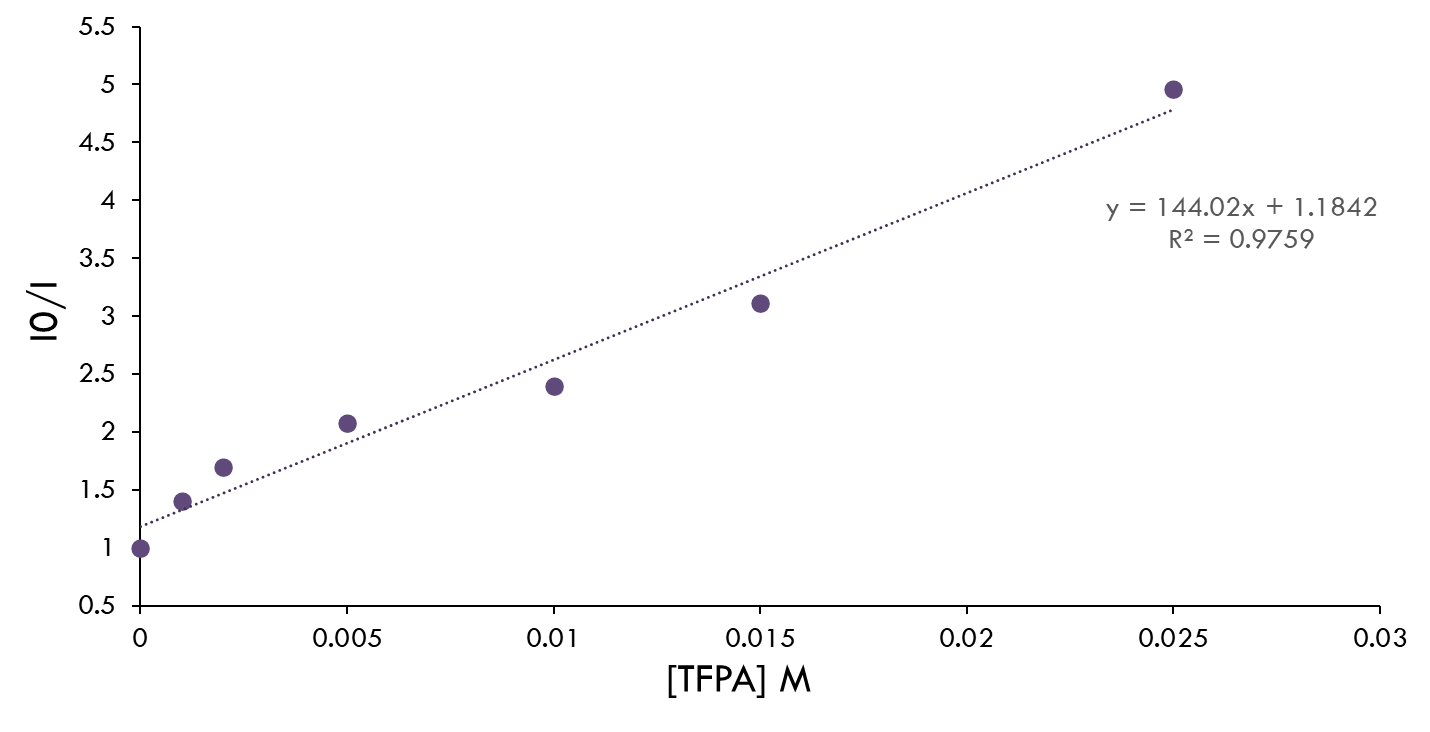


**Figure S20.** Stern–Volmer plot of Ir(ppy)_3_ with trifluoropyruvic acid (TFPA) in acetonitrile. Calculated quenching constant of $k_{q}=7.58\times{10}^{7}M^{-1}s^{-1}$


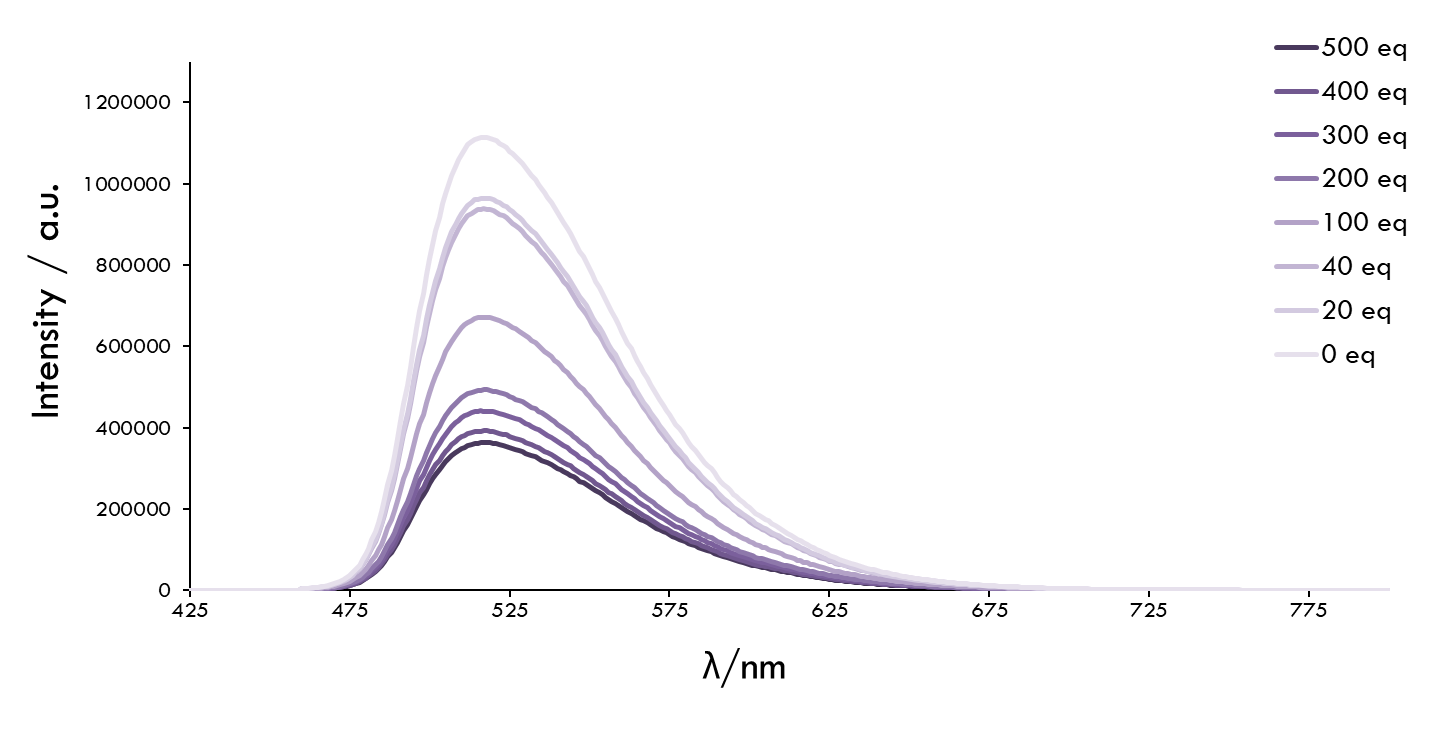


**Figure S21.** Emission quenching of Ir(ppy)_3_ with deuterated trifluoropyruvic acid (d-TFPA) in acetonitrile


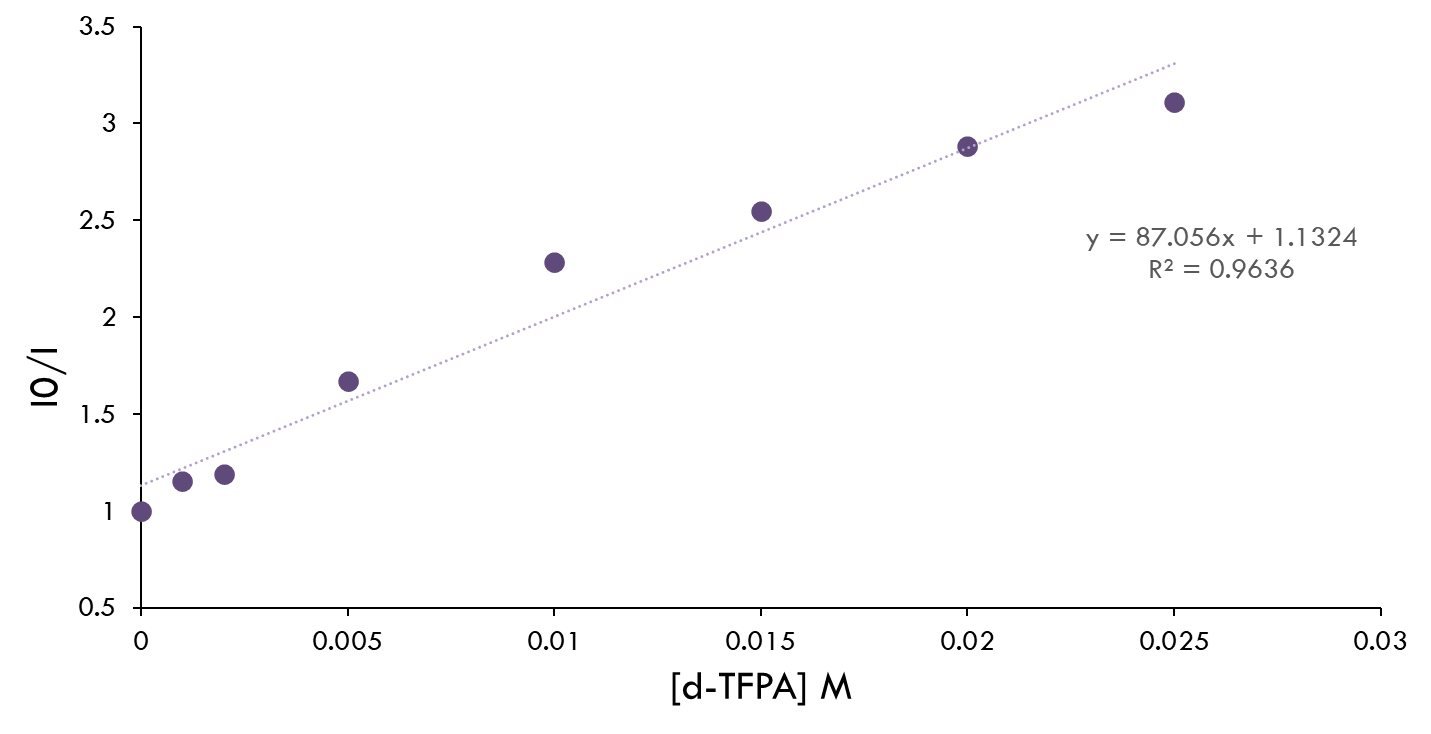


**Figure S22.** Stern–Volmer plot of Ir(ppy)_3_ with deuterated trifluoropyruvic acid (d-TFPA) in acetonitrile. Calculated quenching constant of $k_{q}=4.48\times{10}^{7}M^{-1}s^{-1}$

**Kinetic Isotope Effect Study:**

$$\frac{k_{H}}{k_{D}}= \frac{144.02}{94.541}=1.52$$

# **Kinetic Order Determination**

Kinetic orders of trifluoropyruvic acid **(1)** and 4-methoxystyrene **(S1)** were determined by the method of Variable Time Normalization Analysis (VTNA) developed by Burés.^4^ Kinetic orders were determined by product formation curves through the modification of a given power (β) of the concentration-adjusted x-axis (Σ[B]β Δt) in order to account for the respective influence of a given reaction substrate (B) on the overall rate. The kinetic order that provides the best visual overlap of the product formation curves, suggests the reaction order in substrate. Reaction components studied were evaluated in triplicate and each data point shown is the average of three runs.

VTNA reaction setup for standard conditions (control: 1 equiv styrene, 3 equiv trifluoropyruvic acid).

**Stock Solution**: To a 10-mL volumetric flask was added 32.7 mg of Ir(ppy)_3_ (0.05 mmol, 1 mol%), 2.13 g of trifluoropyruvic acid (15 mmol, 3 equiv) and 5 mL of anhydrous EtOAc. To this solution, was then added 670 mg of 4-methoxystyrene (5 mmol, 1 equiv) and 1.13 g of 4-bromobenzotrifluoride, as an internal standard, (5 mmol, 5 equiv). This solution was then diluted to 10-mL with EtOAc and sonicated for a homogenous solution.

**Reaction setup** [1.5 M trifluoropyruvic acid, 0.5 M styrene]: In triplicate, to an 8-mL vial with a stir bar, 2 mL of the above stock solution was added, capped and purged with inert gas for 10 minutes. An initial time point (t = 0 h) was collected by taking 50 $\mu L$ of the reaction mixture. The reaction mixture was sealed with parafilm and irradiated by Kessil 456 nm light at 100% intensity. Every hour, for the course of 8 hours, an aliquot of 50 $\mu L$ was removed via a syringe, under positive flow of an inert nitrogen gas balloon. The aliquot was diluted with DMSO-d6 and analyzed via ^1^H and ^19^F NMR in order to determine starting material consumption and product formation concentrations. This procedure was varied for styrene and trifluoropyruvic acid concentrations, in order to give the VTNA curves below.

**Trifluoropyruvic acid VTNA**

**Figure S23.** *Order determined by visual inspection of the overlap of product formation curves*

***VTNA suggests that the reaction is first-order in pyruvic acid***

**Styrene VTNA**

**Figure S24.** *Order determined by visual inspection of the overlap of product formation curves*

***VTNA suggests that the reaction is zeroth-order in styrene***

# **General Procedure for the Synthesis of Starting Materials and Characterization**

**1. Preparation of Keto Acids**

- **S2**–**S4** and **4a**–**4b**, were purchased from Combi-blocks and Oakwood chemical, and used as received.

**3,3,3-trifluoro-2-oxopropanoic acid (1)**

3,3,3-trifluoro-2-oxopropanoic acid (trifluoropyruvic acid) was synthesized according to a patent,^5^ with slightly modified reaction conditions. Ethyl trifluoropyruvate (10 mL, 60 mmol, 1.0 equiv) was added to a round bottom flask, followed by addition of EtOH (83.3 mL, 0.98 M). To this solution, an aqueous solution of NaOH (6.3 g, 120 mmol, 2.0 equiv in 83.3 mL of H_2_O) was added dropwise. The reaction was allowed to stir at room temperature for 16 hours. The reaction mixture was then acidified to pH = 1 and extracted with EtOAc (3 x 100mL). The organic layers were combined, dried over Na_2_SO_4_, and the solvent was removed under reduced pressure. Further drying was required on high vac in order to afford the title compound **1**, as a white solid (8.0 g, 95% yield).

**^1^H NMR (500 MHz, DMSO)** *δ* 9.25 (brs, 1H)

**^13^C NMR (126 MHz, DMSO)** *δ* 168.8, 122.9 (q, *J* = 288.2 Hz), 90.9 (q, *J* = 31.8 Hz)

**^19^F NMR (376 MHz, DMSO)** *δ* -81.4 (s, 3F)

**HRMS AMM (ESI–TOF)** m/z calculated for C3F3O3- [M**–**H]**^–^** 140.9805, found 140.9802

**3,3,3-trifluoro-2-oxopropanoic acid-*d* (1-*d*)**

To a round bottom flask, 3,3,3-trifluoro-2-oxopropanoic acid (8.0 g, 56.3 mmol, 1.0 equiv) in D_2_O (30 mL, 0.03 M) was added and heated to 80 $^{\circ}$C for 16 hours, while vigorously stirring. Afterwards, the mixture is diluted with anhydrous acetonitrile and lyopholization of the reaction mixture afforded **1–*d*** as a white solid (8.0 g).

**^13^C NMR (126 MHz, DMSO**) *δ* 168.0, 122.5 (q, *J* = 288.3 Hz), 90.5 (q, *J* = 31.9 Hz)

**^19^F NMR (376 MHz, CDCl_3_)** *δ*-81.6 (s, 3F)

**HRMS AMM (ESI–TOF)** m/z calculated for C3F3O3- [M**–**D]**^–^** 140.9805, found 140.9802

**2. Preparation of Substrates**

- Electron-­rich styrenes **S1** and **S4**–**­S8** were purchased from Combi-blocks and Oakwood chemical, and used as received.

**General Procedure for the Synthesis of Alkenes: S2**–**­S3, S9**–**­12**

Following the procedure of reported literature,^6^ a solution of methyltriphenylphosphonium bromide (1.32 g, 1.2 equiv) in dry THF (0.2 M) was cooled to -78$^{\circ}$C. *n*-BuLi (1.8 mL, 2.5 M in hexanes, 1.5 equiv) was added dropwise under inert atmosphere. The reaction mixture was allowed to stir for 30 minutes and the respective ketone (1 equiv) was added. The reaction mixture was allowed to stir at room temperature, until starting material was consumed by TLC. After the reaction was complete, saturated NH_4_Cl (50 mL) and EtOAc (30 mL) were added, the layers were separated, and the aqueous layer was extracted with EtOAc (3 × 30 mL). The organic extracts were combined, dried over Na_2_SO_4_, filtered and concentrated in vacuo. The residue was purified by flash column chromatography (hexanes/EtOAc = 20:1) to afford **S2**–**­S3, S9**–**­12**.

**1,2,3-trimethoxy-5-vinylbenzene (S2)**

**^1^H NMR (500 MHz, CDCl_3_)** *δ* 6.65­6.59 (dd, *J* = 10.77, 4.73 Hz 1H), 6.62 (s, 1H), 5.65 (d, *J* = 17.5 Hz, 1H), 5.20 (d, *J* = 10.7 Hz, 1H), 3.86 (s, 6H), 3.84 (s, 3H)

**tert-butyl 3-vinyl-1H-indole-1-carboxylate (S10)**

**^1^H NMR (500 MHz, CDCl_3_)** *δ* 8.17 (d, *J* = 7.7 Hz, 1H), 7.80 (d, *J* = 7.8 Hz, 1H), 7.63 (s, 1H), 7.37 – 7.23 (m, 2H), 6.82 (ddd, *J* = 17.8, 11.3, 1.8 Hz, 1H), 5.81 (d, *J* = 17.8 Hz, 1H), 5.33 (d, *J* = 11.3 Hz, 1H), 1.68 (s, 9H)

**9-methylene-9H-thioxanthene (S11)**

**^1^H NMR (500 MHz, CDCl_3_)** *δ* 7.65­7.61 (m, 2H), 7.38­7.35 (m, 2H), 7.28­7.24 (m, 4H), 5.56 (s, 2H)

**1,1-Diphenylethylene (S12)**

**^1^H NMR (500 MHz, CDCl_3_)** δ 7.40­7.36 (m, 10 H), 5.51 (s, 2H)

**Synthesis of 7-methoxy-1,2-dihydronaphthalene (S9)**

*Step 1*. Following reported patent,^7^ NaBH_4_ (5 equiv) was added to a solution of 4-methoxytetralone (1 equiv) in MeOH (0.2 M), at 0$^{\circ}$C. The solution was allowed to stir for 1 hour before diluting with water and extracted with DCM (3 x 100 mL). The organic extracts were combined, dried over Na_2_SO_4_, filtered and concentrated in vacuo. The resulting yellow oil (**S9a**) was used in the next step of the reaction without any further purification.

*Step 2.* Following a reported patent,^4^ 6-Methoxy-1,2,3,4-tetrahydronaphthalen-1-ol (**S9a**, 1 equiv), THF (2.0 M) and *p*-toluenesulfonic acid monohydrate (5 mol%) were added to a round bottom flask. The reaction mixture was allowed to reflux for 1 hour. The reaction mixture was then cooled and washed with sat. NaHCO_3_, brine, and extracted with DCM (3 x 100 mL). The organic extracts were combined, dried over Na_2_SO_4_, filtered and concentrated in vacuo. The residue was purified by flash column chromatography (hexanes/EtOAc = 10:1) to give **S9** as a yellow oil (95% yield).

**^1^H NMR (500 MHz, CDCl_3_)** *δ* 7.26 (d, *J* = 9.0 Hz, 1H), 7.03 – 6.97 (m, 2H), 6.74 (dt, *J* = 9.6, 1.9 Hz, 1H), 6.21 (dt, *J* = 9.6, 4.3 Hz, 1H), 4.09 (s, 3H), 3.09 (t, *J* = 8.2 Hz, 2H), 2.60 (tdd, *J* = 7.9, 4.3, 1.8 Hz, 2H)

**Synthesis of tert-butyl 3-formyl-1H-indole-1-carboxylate (S10a)**

Following reported literature,^8^ triethylamine (1.2 equiv) and DMAP (10 mol%) were added to a solution of indole-3-carboxaldehyde (1 equiv) in DCM (0.2 M). To this solution, Boc_2_O (1.3 equiv) was added and allowed to stir for 1 hour. After the reaction was complete, the mixture was extracted with DCM (3 x 100 mL). The organic extracts were combined, dried over Na_2_SO_4_, filtered and concentrated in vacuo. The residue was purified by flash column chromatography (hexanes/EtOAc = 20:1) to give **S10a** as a white solid (quantitative yield).

**^1^H NMR (500 MHz, CDCl_3_)** *δ* 10.10 (s, 1H), 8.29 (dd, *J* = 7.7, 1.2 Hz, 1H), 8.23 (s, 1H), 8.15 (d, *J* = 8.2 Hz, 1H), 7.44 – 7.35 (m, 2H), 1.71 (s, 9H)

# **General Procedure for PCET Lactonization and Characterization**

*Condition A: Trifluoropyruvic Acid*

To an oven dried 8-mL vial with a stir bar was added photocatalyst Ir(ppy)_3_ (3.25 mg, 0.005 mmol, 1 mol%), trifluoropyruvic acid (213 mg, 1.5 mmol, 3 equiv) and anhydrous EtOAc (1 mL, 0.5 M). The vial was capped and degassed with bubbling nitrogen stream for 15 minutes. The volatile liquid alkenes (0.5 mmol, 1 equiv) are then added and the headspace is purged for an additional 1 minute. The vial is then sealed with parafilm and irradiated using 34 W Kessil PR160L 456 nm (1 cm away, with cooling fan to keep the reaction at room temperature), while stirring at 500 RPM. After 16 hours, the reaction is taken off of the Kessil lamp, and the parafilm and cap are removed. Trifluorotoluene (internal standard, 0.5 mmol) was then added and the reaction mixture, recapped, and allowed to stir at 200 RPM for 5 minutes. An aliquot was then dissolved in CDCl_3_ and was analyzed by ^19^F NMR. The vial was opened, diluted with DCM, and reduced under pressure. The crude material was then dry loaded onto silica for flash column chromatography.

*Note: After purification, hydroxylactones often held on to solvents such as EtOAc and could not be removed by simple high-vac. Small amounts of ethyl acetate were removed by concentrating the material with chloroform and then allowed to evaporate on high-vac.*


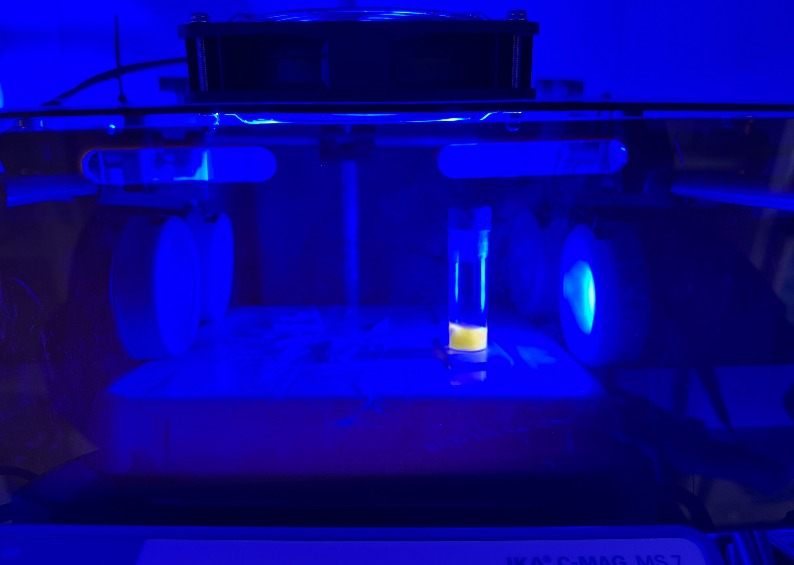


**Figure S25**.*Typical setup for 0.5 mmol reaction*

*Condition B: Keto Acids*

To an oven dried 8-mL vial with a stir bar was added photocatalyst Ir(ppy)_3_ (3.25 mg, 0.005 mmol, 1 mol%), keto-acid (0.5 mmol, 1 equiv) and anhydrous EtOAc (1 mL, 0.5 M). The vial was capped and degassed with bubbling nitrogen stream for 15 minutes. Diphenyl styrene was then added to the reaction mixture (270.4 mg, 1.5 mmol, 3 equiv) and the headspace is purged for an additional 1 minute. The vial is then sealed with parafilm and irradiated using 34 W Kessil PR160L 456 nm (1 cm away, with cooling fan to keep the reaction at room temperature), while stirring at 500 RPM. After 16 hours, the reaction mixture was concentrated and 1,3,5 ­trimethoxybenzene (internal standard, 0.5 mmol) was added to the crude material. The crude material was dissolved in CDCl_3_ and allowed to stir at 200 RPM for 5 minutes. The crude material was analyzed by ^1^H NMR and then dry loaded onto silica for flash column chromatography.

*Note: After purification, hydroxylactones often held on to solvents such as EtOAc and could not be removed by simple high-vac. Small amounts of ethyl acetate were removed by concentrating the material with chloroform and then allowed to evaporate on high-vac.*

**3-hydroxy-5-(4-methoxyphenyl)-3-(trifluoromethyl)dihydrofuran-2(3H)-one (5)**

**Small scale**: Prepared following the general procedure condition A. The crude material was purified by flash column chromatography (hexanes/EtOAc = 6:1) to afford 132.0 mg of **5** in 96% yield as a 2.8:1 diastereomeric mixture, isolated as a white solid.

**Gram scale**: To an oven dried 50 mL round bottom flask equipped with a stir bar was added photocatalyst Ir(ppy)_3_ (48.0 mg, 0.074 mmol, 1 mol%), trifluoropyruvic acid (3.2 g, 7.46 mmol, 3.0 equiv), and anhydrous EtOAc (14.9 mL, 0.5 M). The round bottom flask was capped with a septum and degassed with bubbling nitrogen stream for 15 minutes. Olefin (1 mL, 7.46 mmol, 1.0 equiv) was added and the headspace is purged for an additional 1 minute. The round bottom flask is then sealed with parafilm and irradiated using two 34 W Kessil PR160L 456 nm (2 cm away, with cooling fan to keep the reaction at room temperature) for 16 hours. The flask was opened and reduced under pressure. The crude material was purified by flash column chromatography (hexanes/EtOAc = 6:1) to afford 1.9 g of **5** in 93% yield as a white solid.


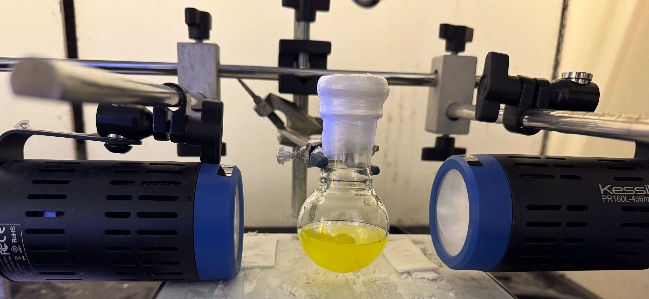

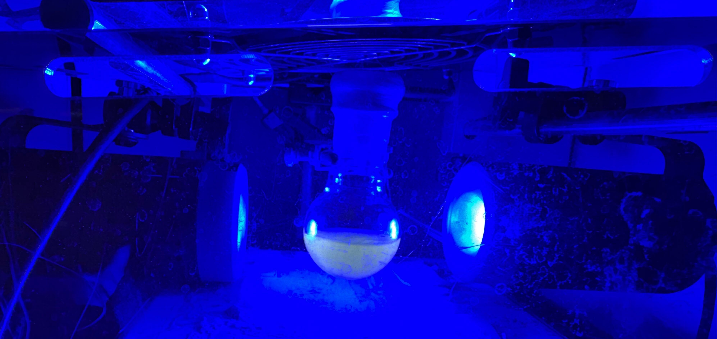


**Figure S26**. Set-up for gram-scale reaction

**Major Diastereomer:**

**^1^H NMR (500 MHz, CDCl_3_)** *δ* 7.31–7.27 (m, 2 H), 6.95–6.93 (m, 2H), 5.49 (dd, *J* = 10.0, 6.3 Hz, 1H), 3.83 (s, 1H), 3.14 (dd, *J* = 14.5, 6.3 Hz, 1H), 2.58–2.52 (m, 1H)

**^13^C NMR (126 MHz, CDCl_3_)** *δ* 171.5, 160.6, 128.8, 127.9, 125.71 (q, *J* = 277.2 Hz), 114.58, 79.95, 76.64 (q, *J* = 37.8 Hz), 55.53, 39.20

**^19^F NMR (376 MHz, CDCl_3_)** *δ* -79.9 (s, 3F)

**Minor Diastereomer:**

**^1^H NMR (500 MHz, CDCl_3_)** *δ* 7.31–7.27 (m, 2 H), 6.95–6.93 (m, 2H), 5.66 (dd, *J* = 9.7, 6.0 Hz, 1H), 3.83 (s, 1H), 2.78 (dd, *J* = 14.3, 6.0 Hz, 1H), 2.58–2.52 (m, 1H)

**^13^C NMR (126 MHz, CDCl_3_)** *δ* 170.3, 160.5, 128.6, 127.5, 125.28 (q, *J* = 327.6 Hz), 114.56, 79.02, 76.51 (q, *J* = 32.76 Hz), 55.53, 39.1

**^19^F NMR (376 MHz, CDCl_3_)** *δ* -79.8 (s, 3F)

**HRMS AMM (ESI–TOF)** m/z calculated for C_12_H_12_F_3_O_4_^+^ [M**+**H]**^+^** 277.0688, found 277.0679

**3-hydroxy-3-(trifluoromethyl)-5-(3,4,5-trimethoxyphenyl)dihydrofuran-2(3H)-one (6)**

Prepared following the general procedure condition A. The crude material was purified by flash column chromatography (hexanes/EtOAc = 6:1) to afford 154.60 mg of **6** in 92% yield as a 2:1 diastereomeric mixture, isolated as a white solid.

**Major Diastereomer:**

**^1^H NMR (500 MHz, CDCl_3_)** *δ* 6.56 (s, 1H), 5.46 (dd, *J* = 9.9, 6.4 Hz, 2H), 3.86 (s, 9H), 3.17 (dd, *J* = 14.6, 6.4 Hz, 1H), 2.52 (m, 1H)

**^13^C NMR (126 MHz, CDCl_3_)** *δ* 171.5, 153.84, 138.7, 132.7, 123.4 (q, *J* = 286.0 Hz), 103.0, 79.10, 76.3 (q, *J* = 31.5 Hz), 60.99, 56.38, 39.4

**^19^F NMR (376 MHz, CDCl_3_)** *δ* -79.7 (s, 3F)

**Minor Diastereomer:**

**^1^H NMR (500 MHz, CDCl_3_)** δ 6.52 (s, 1H), 5.64 (dd, *J* = 9.6, 6.0 Hz, 1H), 3.84 (s, 9H), 2.82 (dd, *J* = 14.2, 6.0 Hz, 1H), 2.52 (m, 1H)

**^13^C NMR (126 MHz, CDCl_3_)** *δ* 170.2, 153.84, 138.6, 132.6, 123.0 (q, *J* = 283.5 Hz), 102.6, 78.9, 76.7 (q, *J* = 31.5 Hz), 60.99, 56.38, 39.5

**^19^F NMR (376 MHz, CDCl_3_)** *δ* -79.8 (s, 3F)

**HRMS AMM (ESI–TOF)** m/z calculated for C_14_H_15_F_3_NaO_6_^+^ [M**+**Na]**^+^** 359.0718, found 359.0706

**3-hydroxy-5-(2-methoxyphenyl)-3-(trifluoromethyl)dihydrofuran-2(3H)-one (7)**

Prepared following the general procedure condition A. The crude material was purified by flash column chromatography (hexanes/EtOAc = 6:1) to afford 129.7 mg of **7** in 94% yield as a 4.8:1 diastereomeric mixture, isolated as a white solid.

**Major Diastereomer:**

**^1^H NMR (500 MHz, CDCl_3_)** *δ* 7.37–7.32 (m, 2H), 7.02–6.91 (m, 2H), 5.92 (dd, *J* = 8.93, 1.76 Hz, 1H), 3.85 (s, 3H), 3.77 (s, 1H) 2.90 (dd, *J* = 14.5, 5.45 Hz, 1H), 2.60–2.51 (m, 1H)

**^13^C NMR (126 MHz, CDCl_3_)** *δ* 170.6, 156.4, 130.2, 126.61, 126.19, 125.40, 122.9 (q, *J* = 338.9 Hz), 120.84, 76.3 (q, *J* = 32.7 Hz), 76.11, 55.4, 37.5

**^19^F NMR (376 MHz, CDCl_3_)** *δ* -79.9 (s, 3F)

**Minor Diastereomer:**

**^1^H NMR (500 MHz, CDCl_3_)** *δ* 7.37–7.32 (m, 2H), 7.02–6.91 (m, 2H), 5.74 (dd, *J* = 6.74, 2.32 Hz, 1H), 3.85 (s, 3H), 3.69 (s, 1H), 3.22 (dd, *J* = 14.2, 5.0 Hz, 1H), 2.60–2.51 (m, 1H)

**^13^C NMR (126 MHz, CDCl_3_)** *δ* 171.7, 156.5, 130.4, 126.61,126.19, 125.40, 246.8 (q, *J* = 286.0 Hz), 120.86, 76.1 (q, *J* = 46.6 Hz), 76.11, 55.5, 37.4

**^19^F NMR (376 MHz, CDCl_3_)** *δ* -80.2 (s, 3F)

**HRMS AMM (ESI–TOF)** m/z calculated for C_12_H_11_F_3_NaO_4_^+^ [M**+**Na]**^+^** 299.0507, found 299.0496

**4-(4-hydroxy-5-oxo-4-(trifluoromethyl)tetrahydrofuran-2-yl)phenyl acetate (8)**

Prepared following the general procedure condition A. The crude material was purified by flash column chromatography (hexanes/EtOAc = 6:1) to afford 106.4 mg of **8** in 70% yield as a 3.6:1 diastereomeric mixture, isolated as a yellow oil.

**Major Diastereomer:**

**^1^H NMR (500 MHz, CDCl_3_)** *δ* 7.40–7.35 (m, 2H), 7.18–7.15 (m, 2H), 5.70 (dd, *J* = 9.6, 6.1 Hz, 1H), 3.66 (brs, 1H), 2.83 (dd, *J* = 14.3, 6.1 Hz, 1H), 2.56–2.50 (m, 1H)

**^13^C NMR (126 MHz, CDCl_3_)** *δ* 169.96, 151.26, 134.59, 126.99, 122.96 (q, *J* = 283.44 Hz), 122.38, 115.99, 78.51, 76.54 (q, *J* =32.77 Hz), 36.55, 27.53, 21.15

**^19^F NMR (376 MHz, CDCl_3_)** *δ* -79.79 (s, 3F)

**Minor Diastereomer:**

**^1^H NMR (500 MHz, CDCl_3_)** *δ* 7.40–7.35 (m, 2H), 7.18–7.15 (m, 2H), 5.53 (dd, *J* = 10.0, 6.4 Hz, 1H), 3.66 (brs, 1H), 3.19 (dd, *J* = 14.6, 6.4 Hz, 1H), 2.56–2.50 (m, 1H)

**^13^C NMR (126 MHz, CDCl_3_)** *δ* 170.12, 151.37, 134.86, 127.77, 285.74 (q, *J* = 122.96 Hz), 122.40, 115.99, 78.19, 76.50 (q, *J* = 32.09 Hz), 39.37, 27.53, 21.15

**^19^F NMR (376 MHz, CDCl_3_)** *δ* -79.76 (s, 3F)

**HRMS AMM (ESI–TOF)** m/z calculated for C_13_H_11_F_3_NaO_5_^+^ [M**+**Na]**^+^** 327.0456, found 327.0454

**5-(4-bromophenyl)-3-hydroxy-3-(trifluoromethyl)dihydrofuran-2(3H)-one (9)**

Prepared following the general procedure condition A. The crude material was purified by flash column chromatography (hexanes/EtOAc = 6:1) to afford 92.3 mg of **9** in 57% yield as a 4:1 diastereomeric mixture, isolated as a white solid.

**Major Diastereomer (8a):**

**^1^H NMR (500 MHz, CDCl_3_)** *δ* 7.59–7.57 (m, 2H), 7.24 (d, *J* = 8.4 Hz, 2H), 5.70 (dd, *J* = 9.5, 6.1 Hz, 1H), 4.16 (s, 1H), 3.22 (dd, *J* = 14.6, 6.5 Hz), 2.53–2.47 (m, 1H)

**^13^C NMR (126 MHz, CDCl_3_)** *δ* 170.1, 135.9, 132.40, 127.3, 123.7 (q, *J* = 338.9 Hz), 123.53, 78.4, 76.6 (q, *J* = 32.76 Hz), 39.21

**^19^F NMR (376 MHz, CDCl_3_)** *δ* -79.9 (s, 3F)

**Minor Diastereomer (8b):**

**^1^H NMR (500 MHz, CDCl_3_)** *δ* 7.59–7.57 (m, 2H), 7.28 (d, *J* = 3.5 Hz, 2H), 5.52 (dd, *J* = 9.8, 6.5 Hz), 4.02 (s, 1H), 2.87 (dd, *J* = 14.3, 6.1 Hz), 2.53–2.47 (m, 1H)

**^13^C NMR (126 MHz, CDCl_3_)** *δ* 170.4, 136.2, 132.44, 127.6, 123.9 (q, *J* = 336.42 Hz), 123.53, 78.1, 76.4 (q, *J* = 32.76 Hz), 39.24

**^19^F NMR (376 MHz, CDCl_3_)** *δ* -80.0 (s, 3F)

**HRMS AMM (ESI–TOF)** m/z calculated for C_11_H_7_BrF_3_O_3_^-^ [M**–**H]**^–^** 322.9536, found 322.9533

**5-(4-chlorophenyl)-3-hydroxy-3-(trifluoromethyl)dihydrofuran-2(3H)-one (10)**

Prepared following the general procedure condition A. The crude material was purified by flash column chromatography (hexanes/EtOAc = 6:1) to afford 77.0 mg of **10** in 55% yield as a 1.6:1 diastereomeric mixture, isolated as a white solid.

**Major Diastereomer:**

**^1^H NMR (500 MHz, CDCl_3_)** *δ* 7.38 (d, *J* = 8.5 Hz, 1H), δ 7.28–7.23 (m, 2H), δ 5.68 (dd, *J* = 9.5 ,6.1 Hz, 1H), δ 2.83 (dd, *J* = 14.5 ,6.5 Hz, 1H), δ 2.49 (dd, *J* = 14.3 ,9.5 Hz, 1H)

**^13^C NMR (126 MHz, CDCl_3_)** *δ* 170.3, 135.60, 135.43, 129.4, 129.4, 127.3, 127.1, 123.8 (q, *J* = 283.2 Hz), 78.4, 76.58 (q, *J* = 32.8), 39.3

**^19^F NMR (376 MHz, CDCl_3_)** δ -79.9 (s, 3F)

**Minor Diastereomer:**

**^1^H NMR (500 MHz, CDCl_3_)** *δ* 7.38 (d, *J* = 8.47 Hz, 1H), δ 7.28–7.23 (m, 2H), δ 5.5 (dd, *J* = 9.8, 6.4 Hz, 1H), δ 3.18 (dd, *J* = 14.5, 6.4 Hz, 1H), δ 2.49­–2.44 (m, 1H)

**^13^C NMR (126 MHz, CDCl_3_)** *δ* 171.5, 135.68, 135.60, 129.4, 129.4, 127.3, 127.1, 123.3 (q, *J* = 285.6 Hz), 78.4, 76.52 (q, *J* = 31.9), 39.3

**^19^F NMR (376 MHz, CDCl_3_)** *δ* -79.8 (s, 3F)

**HRMS AMM (ESI–TOF)** m/z calculated for C_11_H_7_ClF_3_O_3_^-^ [M**–**H]**^–^** 279.0041, found 279.0033

**5-(4-fluorophenyl)-3-hydroxy-3-(trifluoromethyl)dihydrofuran-2(3H)-one (11)**

Prepared following the general procedure condition A. The crude material was purified by flash column chromatography (hexanes/EtOAc = 6:1) to afford 77.8 mg of **11** in 59% yield as a 2.5:1 diastereomeric mixture, isolated as a white solid.

**Major Diastereomer:**

**^1^H NMR (500 MHz, CDCl_3_)** *δ* 7.37–7.41 (m, 2H), 7.14–7.40 (m, 2H), 5.69 (dd, *J* = 9.5, 6.1 Hz), 2.84 (dd, *J* = 14.3, 6.2 Hz), 2.53–2.48 (m, 2H).

**^13^C NMR (126 MHz, CDCl_3_)** *δ* 170.2, 164.2, 162.2, 132.6, 127.7, 120.5 (*q*, *J* =283.6 Hz), 116.2, 78.6, 76.6 (q, *J* = 32.8 Hz), 39.3.

**^19^F NMR (376 MHz, CDCl_3_)** *δ* -79.9 (s, 3F), 111.7 (s, 1F).

**Minor Diastereomer:**

**^1^H NMR (500 MHz, CDCl_3_)** *δ* 7.37–7.41 (m, 2H), 7.14–7.40 (m, 2H), 5.52 (dd, *J* = 10.0, 6.4 Hz, 3.19 (dd, *J* = 14.5, 6.4 Hz), 2.53–2.48 (m, 2H).

**^13^C NMR (126 MHz, CDCl_3_)** *δ* 174.5, 164.3, 162.3, 132.9, 128.1, 125.3 (q, *J* = 229.6 Hz), 116.2, 78.2, 76.6 (q, *J* = 31.9 Hz), 39.2.

**^19^F NMR (376 MHz, CDCl_3_)** *δ* -79.8 (s, 3F), 111.4 (s, 1F).

**HRMS AMM (ESI–TOF)** m/z calculated for C_11_H_7_F_4_O_3_^-^ [M**–**H]**^–^** 263.0337, found 263.0331

**5-(4-(tert-butyl)phenyl)-3-hydroxy-3-(trifluoromethyl)dihydrofuran-2(3H)-one (12)**

Prepared following the general procedure condition A. The crude material was purified by flash column chromatography (hexanes/EtOAc = 6:1) to afford 128.4 mg of **12** in 85% yield as a 4.2:1 diastereomeric mixture, isolated as a white solid.

**Major Diastereomer:**

**^1^H NMR (500 MHz, CDCl_3_)** *δ* 7.47–7.44 (m, 2H), 7.32–7.27 (m, 2H), 5.71 (dd, *J* = 9.7, 6.0 Hz, 1H), 3.96 (s, 1H), 3.17 (dd, *J* = 14.6, 6.4 Hz, 1H), 2.60–2.54 (m, 1H), 1.33 (s, 9H)

**^13^C NMR (126 MHz, CDCl_3_)** *δ* 170.5, 152.8, 133.8, 122.7 (q, J = 283.5 Hz), 126.11, 125.7, 79.3, 76.5(q, *J* = 32.7 Hz), 39.2, 34.86, 31.4

**^19^F NMR (376 MHz, CDCl_3_)** *δ* -79.9 (s, 3F),

**Minor Diastereomer:**

**^1^H NMR (500 MHz, CDCl_3_)** *δ* 7.47–7.44 (m, 2H), 7.32–7.27 (m, 2H), 5.53 (dd, *J* = 10.0, 6.4 Hz, 1H), 3.85 (s, 1H), 2.60–2.54 (m, 1H), 1.33 (s, 9H)

**^13^C NMR (126 MHz, CDCl_3_)** *δ* 171.7, 153.0, 134.1, 128.2 (q, *J* = 322.5 Hz), 126.15, 125.2, 78.9, 76.6 (q, *J* = 31.5 Hz), 39.2, 34.88, 31.4

**^19^F NMR (376 MHz, CDCl_3_)** *δ* -79.8 (s, 3F)

**HRMS AMM (ESI–TOF)** m/z calculated for C_15_H_16_F_3_O_3_^-^ [M**–**H]**^–^** 301.1057, found 301.1051

**3-hydroxy-3-(trifluoromethyl)-3a,4,5,9b-tetrahydronaphtho[1,2-b]furan-2(3H)-one (13)**

Prepared following the general procedure condition A. The crude material was purified by flash column chromatography (hexanes/EtOAc = 6:1) to afford 104.2 mg of **13** in 69% yield as a 1.3:1 diastereomeric mixture, isolated as a white solid.

**Major Diastereomer:**

**^1^H NMR (500 MHz, CDCl_3_)** *δ* 7.38 (d, *J* = 8.5 Hz, 1H), 6.85 (dd, *J* = 8.5, 2.6 Hz, 1H), 6.69 (d, *J* = 2.6 Hz, 1H), 5.71 (d, *J* = 4.5 Hz), 5.57 (d, *J* = 5.5 Hz, 1H), 3.81 (s, 3H), 3.08–3.04 (m, 1H), 2.88–2.66 (m, 2H), 2.13–2.08 (m, 1H), 1.54 (qd, *J* = 12.8, 4.2 Hz, 1H)

**^13^C NMR (126 MHz, CDCl_3_)** *δ* 171.7, 160.5, 139.0, 132.7, 122.9 (q, *J* = 282.2 Hz), 121.4, 113.54, 113.1, 79.7 (q, *J* = 31.5 Hz), 55.4, 44.9, 40.4, 28.3, 19.4.

**^19^F NMR (376 MHz, CDCl_3_)** *δ* 78.7 (s, 3F)

**Minor Diastereomer:**

**^1^H NMR (500 MHz, CDCl_3_)** *δ* 7.38 (d, *J* = 8.5 Hz, 1H), 6.85 (dd, *J* = 8.5, 2.6 Hz, 1H), 6.69 (d, *J* = 2.6 Hz, 1H), 5.71 (d, *J* = 4.5 Hz), 5.57 (d, *J* = 5.5 Hz, 1H), 3.81 (s, 3H), 2.88–2.66 (m, 3H), 2.04 (d, *J* = 13.4 Hz, 1H), 1.64 (qd, J = 12.09, 4.18 Hz, 1H),

**^13^C NMR (126 MHz, CDCl_3_)** *δ* 169.6, 160.5, 139.6, 132.5, 123.6 (q, *J* = 287.2 Hz), 121.7, 113.49, 113.3, 78.6 (q, *J* = 30.4 Hz), 55.4, 44.9, 40.4, 27.8, 19.2.

**^19^F NMR (376 MHz, CDCl_3_)** *δ* 74.6 (s, 3F)

**HRMS AMM (ESI–TOF)** m/z calculated for C_14_H_14_F_3_O_4_^+^ [M**+**H]**^+^** 303.0844, found 303.0834

*Relative configuration was determined using J values. Stereocenter alpha to carbonyl was set based off of analogy to crystal structure of 8a.*

**tert-butyl 3-(4-hydroxy-5-oxo-4-(trifluoromethyl)tetrahydrofuran-2-yl)-1H-indole-1-carboxylate (14)**

Prepared following the general procedure condition A. The crude material was purified by flash column chromatography (hexanes/EtOAc = 6:1) to afford 86.65 mg of **14** in 45% yield as a 1:1 diastereomeric mixture, isolated as a yellow oil.

**Major Diastereomer:**

**^1^H NMR (500 MHz, CDCl_3_)** *δ* 8.18 (d, *J* = 8.4 Hz, 1H), 7.70 (d, *J* = 17.6 Hz, 1H), 7.56–7.52 (m, 1H), 7.39 (ddd, *J* = 8.4, 7.1, 1.4 Hz, 1H), 7.30–7.28 (m, 1H), 5.96 (dd, *J* = 9.1, 6.6 Hz, 1H), 4.00 (s, 1H), 3.22 (dd, *J* = 14.5, 6.6 Hz, 1H), 2.89–2.81 (m, 1H), 1.68 (s, 9H)

**^13^C NMR (126 MHz, CDCl_3_)** *δ* 171.4, 149.5, 136.0, 127.5, 125.5, 124.6 (q, J = 286.0 Hz) 123.4, 119.3, 116.7, 84.83, 76.4 (q, *J* = 20.16 Hz), 73.4, 36.8, 28.3

**^19^F NMR (376 MHz, CDCl_3_)** *δ* 79.9 (s, 3F)

**Minor Diastereomer:**

**^1^H NMR (500 MHz, CDCl_3_)** *δ* 8.18 (d, *J* = 8.4 Hz, 1H), 7.70 (d, *J* = 17.6 Hz, 1H), 7.56–7.52 (m, 1H), 7.39 (ddd, *J* = 8.4, 7.1, 1.4 Hz, 1H), 7.30–7.28 (m, 1H), 5.81 (dd, *J* = 9.8, 6.5 Hz, 1H), 4.22 (s, 1H), 2.89–2.81 (m, 2H), 1.69 (s, 9H)

**^13^C NMR (126 MHz, CDCl_3_)** *δ* 170.1, 149.5, 136.0, 127.5, 125.4, 124.1 (q, *J* = 278.4 Hz), 123.3, 119.3, 115.84, 84.80, 76.1 (q, *J* = 18.90 Hz), 73.9, 36.9, 28.3

**^19^F NMR (376 MHz, CDCl_3_)** *δ* 79.7 (s, 3F)

**HRMS AMM (APPI–TOF)** m/z calculated for C_18_H_18_F_3_NO_5_^🞄^**^+^** [M^🞄^**^+^**] 385.1137, found 385.1138

**4-hydroxy-4-(trifluoromethyl)-3,4-dihydro-5H-spiro[furan-2,9'-thioxanthen]-5-one (15)**

Prepared following the general procedure condition A. The crude material was purified by flash column chromatography (hexanes/EtOAc = 6:1) to afford 112.6 mg of **15** in 64% yield, isolated as a yellow oil.

**^1^H NMR (500 MHz, CDCl_3_)** *δ* 7.62–7.53 (m, 4H), 7.37–7.28 (m, 4H), 3.54 (s, 1H), 2.95 (d, *J* = 15.1 Hz, 1H), 2.74 (d, *J* = 15.0 Hz, 1H)

**^13^C NMR (126 MHz, CDCl_3_)** *δ* 171.2, 137.9, 137.6, 130.5, 130.4, 128.1, 127.7, 127.2, 127.3, 127.1, 123.2 (q, *J* = 90.7 Hz) 123.2, 83.3, 76.0 (q, *J* = 32.7 Hz), 42.7

**^19^F NMR (376 MHz, CDCl_3_)** *δ* -80.2 (s, 3F)

**HRMS AMM (APPI–TOF)** m/z calculated for C_17_H_12_F_3_O_3_S^+^ [M**+**H]**^+^** 353.0454, found 353.0454

**3-hydroxy-5,5-diphenyl-3-(trifluoromethyl)dihydrofuran-2(3H)-one (16)**

Prepared following the general procedure condition A. The crude material was purified by flash column chromatography (hexanes/EtOAc = 6:1) to afford 138.49 mg of **16** in 86% yield, isolated as a white solid.

**^1^H NMR (500 MHz, CDCl_3_**) *δ* 7.45 – 7.41 (m, 2H), 7.40 – 7.35 (m, 6H), 7.33 – 7.29 (m, 2H), 3.39 (dd, J = 14.8, 0.8 Hz, 1H), 3.30 (d, J = 14.8 Hz, 1H), 3.18 (brs, 1H).

**^13^C NMR (126 MHz, CDCl_3_)** *δ* 169.9, 142.8, 142.6, 128.9, 128.8, 128.5, 128.4, 125.7, 125.1, 121.8 (q, *J* = 233.4 Hz*)*, 76.7, 88.2, (q, *J* = 97.3 Hz) *,*43.4.

**^19^F NMR (376 MHz, CDCl_3_)** *δ* -80.0 (s, 3F)

**HRMS AMM (APPI–TOF)** m/z calculated for C_17_H_14_F_3_O_3_^+^ [M**+**H]**^+^** 323.0890, found 323.0890

**3-hydroxy-5,5-diphenyldihydrofuran-2(3H)-one (17)**

Prepared following the general procedure condition B. The crude material was purified by flash column chromatography (hexanes/EtOAc = 6:1) to afford 102.95 mg of **17** in 81% yield, isolated as a white solid.

**^1^H NMR (500 MHz, CDCl_3_)** *δ* 7.47–7.40 (m, 2H), 7.39 – 7.32 (m, 6H), 7.32 – 7.26 (m, 2H), 4.48 (dd, *J* = 11.2, 7.8 Hz, 1H), 3.48 (dd, *J* = 12.5, 7.8 Hz, 1H), 2.78 (dd, *J* = 12.5, 11.2 Hz, 1H), 1.58 (brs, 1H)

**^13^C NMR (126 MHz, CDCl_3_)** *δ* 176.5, 143.2, 141.9, 128.9, 128.8, 128.4, 128.3, 125.4, 125.4, 86.8, 68.5, 43.6

**HRMS AMM (APPI–TOF)** m/z calculated for C_16_H_15_O_3_^+^ [M**+**H]**^+^** 255.1016, found 255.1017

**3-benzyl-3-hydroxy-5,5-diphenyldihydrofuran-2(3H)-one (18)**

Prepared following the general procedure condition B. The crude material was purified by flash column chromatography (hexanes/EtOAc = 6:1) to afford 110.12 mg of **18** in 64% yield, isolated as a white solid.

**1H NMR (500 MHz, CDCl_3_**) *δ* 7.38 – 7.29 (m, 6H), 7.29 – 7.20 (m, 3H), 7.23 – 7.16 (m, 4H), 6.99 – 6.90 (m, 2H), 3.08 (d, *J* = 13.6 Hz, 1H), 2.93 (dd, *J* = 13.6, 0.8 Hz, 1H), 2.87 (d, *J* = 14.1 Hz, 1H), 2.59 (d, *J* = 14.1 Hz, 1H), 2.39 (brs, 1H).

**^13^C NMR (126 MHz, CDCl_3_)** *δ* 177.2, 143.9, 143.5, 134.5, 130.6, 128.9, 128.8, 128.6, 128.1, 128.1, 127.5, 125.5, 125.3, 86.8, 76.6, 46.9, 43.2.

**HRMS AMM (APPI–TOF)** m/z calculated for C_23_H_21_O_3_^+^ [M**+**H]**^+^** 283.1329, found 283.1335

**3-hydroxy-3-methyl-5,5-diphenyldihydrofuran-2(3H)-one (19)**

Prepared following the general procedure condition B. The crude material was purified by flash column chromatography (hexanes/EtOAc = 6:1) to afford 92.50 mg of **19** in 69% yield, isolated as a white solid.

**^1^H NMR (500 MHz, CDCl_3_)** *δ* 7.48 – 7.39 (m, 4H), 7.38 – 7.30 (m, 4H), 7.31 – 7.26 (m, 2H), 3.19 – 3.10 (m, 2H), 1,57 (brs, 1H), 1.28 (s, 3H)

**^13^C NMR (126 MHz, CDCl_3_)** *δ* 178.1, 144.1, 143.2, 128.9, 128.8, 128.1, 128.1, 125.3, 125.2, 86.5, 73.9, 49.4, 25.3

**HRMS AMM (APPI–TOF)** m/z calculated for C_17_H_17_O_3_^+^ [M**+**H]**^+^** 269.1172, found 269.1174

**3-ethyl-3-hydroxy-5,5-diphenyldihydrofuran-2(3H)-one (20)**

Prepared following the general procedure condition B. The crude material was purified by flash column chromatography (hexanes/EtOAc = 6:1) to afford 63.47 mg of **20** in 45% yield, isolated as a white solid.

**^1^H NMR (500 MHz, CDCl_3_)** *δ* 7.47 – 7.40 (m, 4H), 7.34 (ddd, *J* = 13.6, 8.5, 6.9 Hz, 4H), 7.30 – 7.23 (m, 2H), 3.14 (d, *J* = 13.6 Hz, 1H), 3.06 (dd, *J* = 13.7, 0.8 Hz, 1H), 2.32 (brs, 1H), 1.67 – 1.58 (m, 1H), 1.44 (dq, *J* = 14.7, 7.4 Hz, 1H), 0.92 (t, *J* = 7.4 Hz, 3H).

**^13^C NMR (126 MHz, CDCl_3_)** *δ* 177.8, 144.3, 143.6, 128.9, 128.74, 128.0, 125.4, 125.12 86.8, 46.7, 30.4, 7.3.

**HRMS AMM (APPI–TOF)** m/z calculated for C_18_H_19_O_3_^+^ [M**+**H]**^+^** 283.1329, found 283.1335

# **Procedure for Intermolecular PCET of Ethyl Trifluoropyruvate**

To an oven dried 8-mL vial with a stir bar was added photocatalyst Ir(ppy)_3_ (3.25 mg, 0.005 mmol, 1 mol%) and anhydrous EtOAc (1 mL, 0.5 M). The vial was capped and degassed with bubbling nitrogen stream for 15 minutes. With the needle in the headspace, volatile compounds are then added in the following order: ethyl trifluoropyruvate (0.198 mL, 1.5 mmol, 3.0 equiv), formic acid (18.9 μL, 0.5 mmol, 1.0 equiv), and 4-methoxystyrene (67.0 μL, 0.5 mmol, 1.0 equiv). The headspace is purged for an additional 1 minute. The vial is then sealed with parafilm and irradiated using 34 W Kessil PR160L 456 nm (1 cm away, with cooling fan to keep the reaction at room temperature), while stirring at 500 RPM. After 16 hours, the reaction is taken off of the Kessil lamp, and the parafilm and cap are removed. Trifluorotoluene (internal standard, 0.5 mmol) was then added and the reaction mixture, recapped, and allowed to stir at 200 RPM for 5 minutes. An aliquot was then dissolved in CDCl_3_ and was analyzed by ^19^F NMR. The vial was opened, diluted with DCM, and reduced under pressure. The crude material was then dry loaded onto silica for flash column chromatography (hexanes/EtOAc = 6:1) to afford 98.0 mg of **4a** in 56% yield as a 1.5:1 diastereomeric mixture, isolated as a yellow oil.

**ethyl 4-(formyloxy)-2-hydroxy-4-(4-methoxyphenyl)-2-(trifluoromethyl)butanoate (4a)**

**Major Diastereomer:**

**^1^H NMR (500 MHz, CDCl_3_)** *δ* 7.85 (s, 1H), 7.29 – 7.27 (m, 2H), 6.96 – 6.78 (m, 2H), 6.11 (dd, *J* = 11.5, 2.7 Hz, 1H), 4.42 (qq, *J* = 7.2, 3.6 Hz, 2H), 4.09 (brs, 1H), 3.80 (s, 1H), 2.82 (dd, *J* = 14.6, 11.4 Hz, 1H), 2.26 (ddd, *J* = 14.7, 2.7, 1.0 Hz, 1H), 1.42 (t, *J* = 7.2 Hz, 3H).

**^13^C NMR (126 MHz, CDCl_3_)** *δ* 169.93, 159.86, 159.62, 130.90, 128.13, 123.5 (q, *J* = 286.9 Hz), 114.17, 77.36, 75.2 (q, *J* = 29.5 Hz), 70.28, 64.11, 55.35, 37.33, 13.98.

**^19^F NMR (376 MHz, CDCl_3_)** *δ* -79.1 (s, 3F).

**Minor Diastereomer:**

**^1^H NMR (500 MHz, CDCl_3_)** *δ* 7.98 (s, 1H), 7.29 – 7.27 (m, 2H), 6.96 – 6.78 (m, 2H), 6.03 (ddd, *J* = 7.6, 6.2, 1.0 Hz, 1H), 4.01 (dq, *J* = 10.7, 7.1 Hz, 1H), 3.94 (brs, 1H), 3.79 (s, 1H), 3.66 (dq, *J* = 10.7, 7.2 Hz, 1H), 2.64 (dd, *J* = 7.0, 2.5 Hz, 1H), 1.16 (t, *J* = 7.2 Hz, 3H).

**^13^C NMR (126 MHz, CDCl_3_)** *δ* 168.56, 160.06, 159.91, 129.97, 129.18, 123.3 (q, *J* = 287.0 Hz), 113.84, 77.36, 75.9 (q, *J* = 29.5 Hz), 70.84, 63.93, 55.38, 36.92, 13.62.

**^19^F NMR (376 MHz, CDCl_3_)** *δ* -79.3 (s, 3F).

**HRMS AMM (ESI–TOF)** m/z calculated for C_15_H_17_F_3_NaO_6_^+^ [M**+**Na]**^+^** 373.086, found 373.0817

# **General Procedure for One-Pot Trifluoromethyl Ketone Synthesis**

*Condition A*: **21­**–­**24** & **28**–­**30**:

*Step 1*. An oven dried 8 mL vial was charged with CF_3_-lactone (0.25 mmol. 1 equiv), DCM (1.25 mL, 0.2M), and FeCl_3_ (2.0 mg, 5 mol%). The reaction vial was degassed with a positive flow of nitrogen, for 15 minutes, while stirring. Afterwards, allyl TMS (81.5 $\mu$L, 0.5 mmol, 2 equiv) was added dropwise. The reaction was allowed to stir until consumption of starting material at room temperature for 2 hours. TBAF (250 $\mu$L, 1.0 M/THF, 1 equiv) was added to the vial and stirred for 15 minutes. The reaction was carried on to the next step without any further purification.

*Step 2.* The above reaction mixture was charged with DABCO (28.0 mg, 0.25 mmol, 1 equiv) and Fe(acac)_3_ (88.2 mg, 0.25 mmol, 1 equiv). Next, a positive flow of O_2_ was bubbled into the solution and allowed to stir for 20 minutes. The reaction vial was sealed with electrical tape and parafilm, and irradiated using 34 W Kessil PR160L 390 nm (1 cm away, with cooling fan to keep the reaction at room temperature) for 12 hours. After the reaction was complete, the mixture was washed with brine solution and extracted with DCM (3 x 50 mL). The organic extracts were combined, dried (Na2SO4), filtered and concentrated in vacuo. The residue was dry loaded and purified by flash column chromatography (hexanes/EtOAc = 50:1) to afford **21­**–­**24** & **28**–­**30**.

*Condition B:* **25**–­**27**:

*Step 1*. An oven dried 8 mL vial, CF_3_-lactone (0.25 mmol. 1 equiv), DCM (1.25 mL, 0.2M), and Sc(OTf)_3_ (61.50 mg, 0.50 equiv) were added. The reaction vial was degassed with a positive flow of nitrogen, for 15 minutes, while stirring. Afterwards, allyl TMS (163.0 $\mu$L, 1.0 mmol, 4 equiv) was added dropwise. The reaction was allowed to stir until consumption of starting material, at 40$^{\circ}$C, for 4 hours. *Note*: TBAF was not used. The reaction was carried on to the next step without any further purification.

*Step 2.* To the reaction mixture above, DABCO (28.0 mg, 0.25 mmol, 1 equiv) and Fe(acac)_3_ (88.2 mg, 0.25 mmol, 1 equiv) were added. Next, a positive flow of O_2_ was bubbled into the solution and allowed to stir for 20 minutes. The reaction vial was sealed with electrical tape and parafilm, and irradiated using 34 W Kessil PR160L 390 nm (1 cm away, with cooling fan to keep the reaction at room temperature) for 12 hours. After the reaction was complete, the mixture was washed with brine solution and extracted with DCM (3 x 50 mL). The organic extracts were combined, dried over Na_2_SO_4_, filtered and concentrated in vacuo. The residue was dry loaded and purified by flash column chromatography (hexanes/EtOAc = 50:1) to afford **25**–­**27**.

**1,1,1-trifluoro-4-(4-methoxyphenyl)hept-6-en-2-one (21)**

Prepared following the general procedure condition A. The crude material was purified by flash column chromatography (hexanes/EtOAc = 20:1) to afford 65.97 mg of **21** in 97% yield, isolated as a yellow oil.

**^1^H NMR (500 MHz, CDCl_3_)** *δ* 7.28 – 7.25 (m, 2H), 7.03 – 6.98 (m, 2H), 5.80 (dddd, *J* = 16.8, 10.2, 7.8, 6.4 Hz, 1H), 5.23 – 5.15 (m, 2H), 3.94 (s, 3H), 3.47 (dt, *J* = 14.9, 7.2 Hz, 1H), 3.25 – 3.07 (m, 2H), 2.54 (qt, *J* = 13.9, 7.2 Hz, 2H).

**^13^C NMR (126 MHz, CDCl_3_)** *δ* 190.4 (q, *J* = 35.3 Hz) 158.5, 135.6, 134.8, 128.3, 117.6, 116.3(q, *J* = 292.3 Hz), 114.1, 55.3, 42.4, 40.8, 38.7.

**^19^F NMR (376 MHz, CDCl_3_)** δ -79.5 (s, 3F).

**HRMS AMM (APPI–TOF)** m/z calculated for C_14_H_16_F_3_O_2_^+^ [M**+**H]**^+^** 273.1097, found 273.1100

**1,1,1-trifluoro-4-(3,4,5-trimethoxyphenyl)hept-6-en-2-one (22)**

Prepared following the general procedure condition A. The crude material was purified by flash column chromatography (hexanes/EtOAc = 20:1) to afford 80.54 mg of **22** in 95% yield, isolated as a yellow oil.

**^1^H NMR (500 MHz, CDCl_3_)** *δ* 6.39 (s, 2H), 5.66 (dddd, *J* = 16.5, 10.1, 8.0, 6.1 Hz, 1H), 5.10 – 5.01 (m, 2H), 3.85 (s, 6H), 3.82 (s, 3H), 3.29 (p, *J* = 7.2 Hz, 1H), 3.01 (dd, *J* = 6.9, 3.2 Hz, 2H), 2.47 – 2.29 (m, 2H).

**^13^C NMR (126 MHz, CDCl_3_)** *δ* 191.1 (q, *J* = 35.4 Hz)153.4, 138.5, 137.0, 135.4, 117.8, 117.4 (q, *J* = 292.6 Hz),104.3, 60.9, 56.2, 42.2, 40.7, 39.8.

**^19^F NMR (376 MHz, CDCl_3_)** *δ* -79.5 (s, 3F).

**HRMS AMM (APPI–TOF)** m/z calculated for C_16_H_19_F_3_O_4_^🞄^**^+^** [M^🞄^**^+^**] 332.1235, found 332.1229

**1,1,1-trifluoro-4-(2-methoxyphenyl)hept-6-en-2-one (23)**

Prepared following the general procedure condition A. The crude material was purified by flash column chromatography (hexanes/EtOAc = 20:1) to afford 63.94 mg of **23** in 94% yield, isolated as a yellow oil.

**^1^H NMR (500 MHz, CDCl_3_)** *δ* 7.21 (ddd, *J* = 8.2, 7.4, 1.7 Hz, 1H), 7.12 (dd, *J* = 7.6, 1.7 Hz, 1H), 6.93 – 6.84 (m, 2H), 5.67 (dddd, *J* = 16.8, 10.1, 7.8, 6.4 Hz, 1H), 5.05 – 4.97 (m, 2H), 3.83 (s, 3H), 3.69 (p, *J* = 7.1 Hz, 1H), 3.06 (dd, *J* = 6.8, 0.8 Hz, 2H), 2.53 – 2.36 (m, 2H).

**^13^C NMR (126 MHz, CDCl_3_)** *δ* 190.7 **(**q, J = 34.7 Hz), δ 157.2, 136.1, 130.5, 128.2, 128.0, 120.7, 117.2, 116.8 (q, J = 292.0 Hz), 110.9, 55.3, 40.9, 38.4, 34.2, 29.8.

**^19^F NMR (376 MHz, CDCl_3_)** *δ* -79.5 (s, 3F).

**HRMS AMM (APPI–TOF)** m/z calculated for C_14_H_15_F_3_O_2_^🞄^**^+^** [M^🞄^**^+^**] 272.1024, found 272.1021

**4-(7,7,7-trifluoro-6-oxohept-1-en-4-yl)phenyl acetate (24)**

Prepared following the general procedure condition A. The crude material was purified by flash column chromatography (hexanes/EtOAc = 20:1) to afford 46.51 mg of **24** in 62% yield, isolated as a yellow oil.

**^1^H NMR (500 MHz, CDCl_3_)** *δ* 7.21 – 7.18 (m, 2H), 7.06 – 7.02 (m, 2H), 5.63 (dddd, *J* = 15.9, 10.7, 7.8, 6.4 Hz, 1H), 5.06 – 5.00 (m, 2H), 3.36 (p, *J* = 7.1 Hz, 1H), 3.10 – 2.93 (m, 2H), 2.46 – 2.32 (m, 2H), 2.29 (s, 3H).

**^13^C NMR (126 MHz, CDCl_3_)** *δ* 190.3 (q, *J* = 35.4 Hz), 169.5, 149.6, 140.3, 135.2, 128.4, 121.8, 117.9, 116.7 (q, *J* = 121.4 Hz) 42.2, 40.6, 38.8, 21.2.

**^19^F NMR (376 MHz, CDCl_3_)** *δ* -79.5 (s, 3F).

**HRMS AMM (APPI–TOF)** m/z calculated for C_15_H_16_F_3_O_3_^+^ [M**+**H]**^+^** 301.1046, found 301.1052

**4-(4-bromophenyl)-1,1,1-trifluorohept-6-en-2-one (25)**

Prepared following the general procedure condition B. The crude material was purified by flash column chromatography (hexanes/EtOAc = 20:1) to afford 36.00 mg of **25** in 45% yield, isolated as a yellow oil.

**^1^H NMR (500 MHz, CDCl_3_)** *δ* 7.45 – 7.41 (m, 2H), 7.10 – 7.04 (m, 2H), 5.66 – 5.55 (m, 1H), 5.05 – 5.00 (m, 2H), 3.35 – 3.27 (m, 1H), 3.09 – 2.93 (m, 2H), 2.43 – 2.31 (m, 2H).

**^13^C NMR (126 MHz, CDCl_3_)** *δ* 190.1 (q, *J* = 35.5 Hz) ,141.7, 134.9, 131.9, 129.2, 120.8, 118.1, 116.4 (q, *J* = 292.3 Hz), 41.9, 40.5, 39.0.

**^19^F NMR (376 MHz, CDCl_3_)** *δ* -79.5 (s, 3F).

**HRMS AMM (APPI–TOF)** m/z calculated for C_13_H_13_BrF_3_O^+^ [M**+**H]**^+^** 321.0096, found 321.1487

**4-(4-chlorophenyl)-1,1,1-trifluorohept-6-en-2-one (26)**

Prepared following the general procedure condition B. The crude material was purified by flash column chromatography (hexanes/EtOAc = 20:1) to afford 32.44 mg of **26** in 47% yield, isolated as a yellow oil.

**^1^H NMR (500 MHz, CDCl_3_**) *δ* 7.42 – 7.39 (m, 2H),7.29 – 7.22 (m, 2H), 5.79 – 5.68 (m, 1H), 5.19 – 5.11 (m, 2H), 3.46 (p, *J* = 7.3 Hz, 1H), 3.24 – 3.07 (m, 2H), 2.50 (tq, *J* = 14.1, 7.1 Hz, 2H).

**^13^C NMR (126 MHz, CDCl_3_)** *δ* 190.2 (q, *J* = 35.6 Hz), 141.2, 135.0, 132.8, 129.0, 128.8, 118.2, 116.6 (q, *J* = 292.4 Hz), 42.0, 40.6, 38.9.

**^19^F NMR (376 MHz, CDCl_3_)** *δ* -79.5 (s, 3F).

**HRMS AMM (APPI–TOF)** m/z calculated for C_13_H_12_ClF_3_O^🞄^**^+^** [M^🞄^**^+^**] 276.0529, found 276.0529

**1,1,1-trifluoro-4-(4-fluorophenyl)hept-6-en-2-one (27)**

Prepared following the general procedure condition B. The crude material was purified by flash column chromatography (hexanes/EtOAc = 20:1) to afford 33.81 mg of **27** in 52% yield, isolated as a yellow oil.

**^1^H NMR (500 MHz, CDCl_3_)** *δ* 7.40 (dd, *J* = 8.0, 6.1 Hz, 2H), 7.27 – 7.22 (m, 2H), 5.78 – 5.69 (m, 1H), 5.19 – 5.13 (m, 2H), 3.49 – 3.42 (m, 1H), 3.23 – 3.05 (m, 2H), 2.56 – 2.45 (m, 2H).

**^13^C NMR (126 MHz, CDCl_3_)** *δ* 190.5 (q, *J* = 35.6 Hz), 141.2, 135.0, 132.8, 128.9, 128.8, 118.1, 116.6 (q, *J* = 291.7 Hz), 42.2, 40.6, 38.9.

**^19^F NMR (376 MHz, CDCl_3_)** *δ* -79.5 (s, 3F).

**HRMS AMM (APPI–TOF)** m/z calculated for C_13_H_12_F_4_O^🞄^**^+^** [M^🞄^**^+^**] 260.0824, found 260.0821

**4-(4-(tert-butyl)phenyl)-1,1,1-trifluorohept-6-en-2-one (28)**

Prepared following the general procedure condition A. The crude material was purified by flash column chromatography (hexanes/EtOAc = 20:1) to afford 60.37 mg of **28** in 81% yield, isolated as a yellow oil.

**^1^H NMR (500 MHz, CDCl_3_)** *δ* 7.35 – 7.29 (m, 2H), 7.17 – 7.08 (m, 2H), 5.65 (dddd, *J* = 16.6, 10.1, 7.9, 6.2 Hz, 1H), 5.11 – 4.96 (m, 2H), 3.34 (p, *J* = 7.1 Hz, 1H), 3.10 – 2.94 (m, 2H), 2.48 – 2.31 (m, 2H), 1.30 (s, 9H).

**^13^C NMR (126 MHz, CDCl_3_)** *δ* 190.5 (q, *J* = 35.1 Hz), 149.8, 139.7, 135.7, 127.0, 125.7, 117.6, 114.4 (q, *J* = 292.8 Hz), 42.1, 40.8, 38.8, 34.56, 31.4.

**^19^F NMR (376 MHz, CDCl_3_)** *δ* -79.4 (s, 3F).

**HRMS AMM (APPI–TOF)** m/z calculated for C_17_H_21_F_3_O^🞄^**^+^** [M^🞄^**^+^**] 298.1544, found 298.1544

**3-(9-allyl-9H-thioxanthen-9-yl)-1,1,1-trifluoropropan-2-one (29)**

Prepared following the general procedure condition A. The crude material was purified by flash column chromatography (hexanes/EtOAc = 20:1) to afford 83.54 mg of **29** in 96% yield, isolated as a yellow oil.

**^1^H NMR (500 MHz, CDCl_3_)** *δ* 7.46 – 7.39 (m, 2H), 7.28 – 7.16 (m, 6H), 5.38 (dddd, *J* = 16.0, 10.3, 7.3, 6.2 Hz, 1H), 5.11 – 5.05 (m, 2H), 3.69 (s, 2H), 2.95 (dt, *J* = 6.6, 1.3 Hz, 2H).

**^13^C NMR (126 MHz, CDCl_3_)** *δ* 188.5 (q, *J* = 35.0 Hz) 136.4, 133.5, 132.0, 127.3, 127.0, 126.5, 126.2, 119.2, 116.6 (q, *J* = 290.1 Hz), 45.42, 40.6, 39.5.

**^19^F NMR (376 MHz, CDCl_3_)** *δ* -79.2 (s, 3F).

**HRMS AMM (APPI–TOF)** m/z calculated for C_19_H_16_F_3_OS^+^ [M**+**H]**^+^** 349.0868, found 349.0876

**1-(1-allyl-6-methoxy-1,2,3,4-tetrahydronaphthalen-2-yl)-2,2,2-trifluoroethan-1-one (30)**

Prepared following the general procedure condition A. The crude material was purified by flash column chromatography (hexanes/EtOAc = 20:1) to afford 63.35 mg of **30** in 85% yield, isolated as a brown oil.

**Major Diastereomer:**

**^1^H NMR (500 MHz, CDCl_3_)** *δ* 7.18 (d, *J* = 8.6 Hz, 1H), 6.76 (dd, *J* = 8.6, 2.8 Hz, 1H), 6.60 (d, *J* = 2.7 Hz, 1H), 5.62 (ddt, *J* = 16.1, 11.0, 7.2 Hz, 1H), 5.08 (s, 1H), 5.05 (dd, *J* = 7.5, 1.4 Hz, 1H), 3.78 (s, 3H), 3.43 (td, *J* = 7.2, 4.9 Hz, 1H), 3.24 (ddd, *J* = 8.6, 6.6, 3.9 Hz, 1H), 2.83 – 2.73 (m, 2H), 2.48 – 2.37 (m, 2H), 2.29 – 2.15 (m, 2H), 1.84 (dtd, *J* = 13.7, 8.5, 5.4 Hz, 1H).

**^13^C NMR (126 MHz, CDCl_3_)** *δ* 194.2 (q, *J* = 33.3 Hz), 157.7, 136.8, 135.4, 129.4, 118.2, 113.7 (q, *J* = 131.1 Hz), 113.3, 112.9, 55.3, 45.6, 41.1, 39.14, 36.9, 28.4, 23.7.

**^19^F NMR (376 MHz, CDCl_3_)** *δ* -76.8 (s, 3F)

**Minor Diastereomer:**

**^1^H NMR (500 MHz, CDCl_3_)** *δ* 7.04 (d, *J* = 8.5 Hz, 1H), 6.71 (td, *J* = 8.5, 2.6 Hz, 1H), 6.65 (d, *J* = 2.7 Hz, 1H), 5.76 – 5.68 (m, 1H), 5.08 (s, 1H), 4.92 (dd, *J* = 17.0, 1.6 Hz, 1H), 3.78 (s, 3H), 3.33­3.30 (m, 2H), 2.99 – 2.85 (m, 4H), 2.05 – 1.95 (m, 2H).

**^13^C NMR (126 MHz, CDCl_3_)** *δ* 193.7 (q, *J* = 33.5 Hz), 158.3, 136.8, 135.9, 129.3, 117.8, 114.1 (q, *J* = 144.4 Hz), 113.3, 112.9, 55.3, 46.5, 41.1, 38.89, 37.6, 27.7, 23.7.

**^19^F NMR (376 MHz, CDCl_3_)** - *δ* 77.0.

**HRMS AMM (APPI–TOF)** m/z calculated for C_16_H_17_F_3_O_2_^🞄^**^+^** [M^🞄^**^+^**] 298.1181, found 298.1180

# **X-Ray Diffraction Data**

Crystal Data for Major Diastereomer CCDC **2425907 (9a)**

**Datablock kb_resolve_9009_4_a** - ellipsoid plot


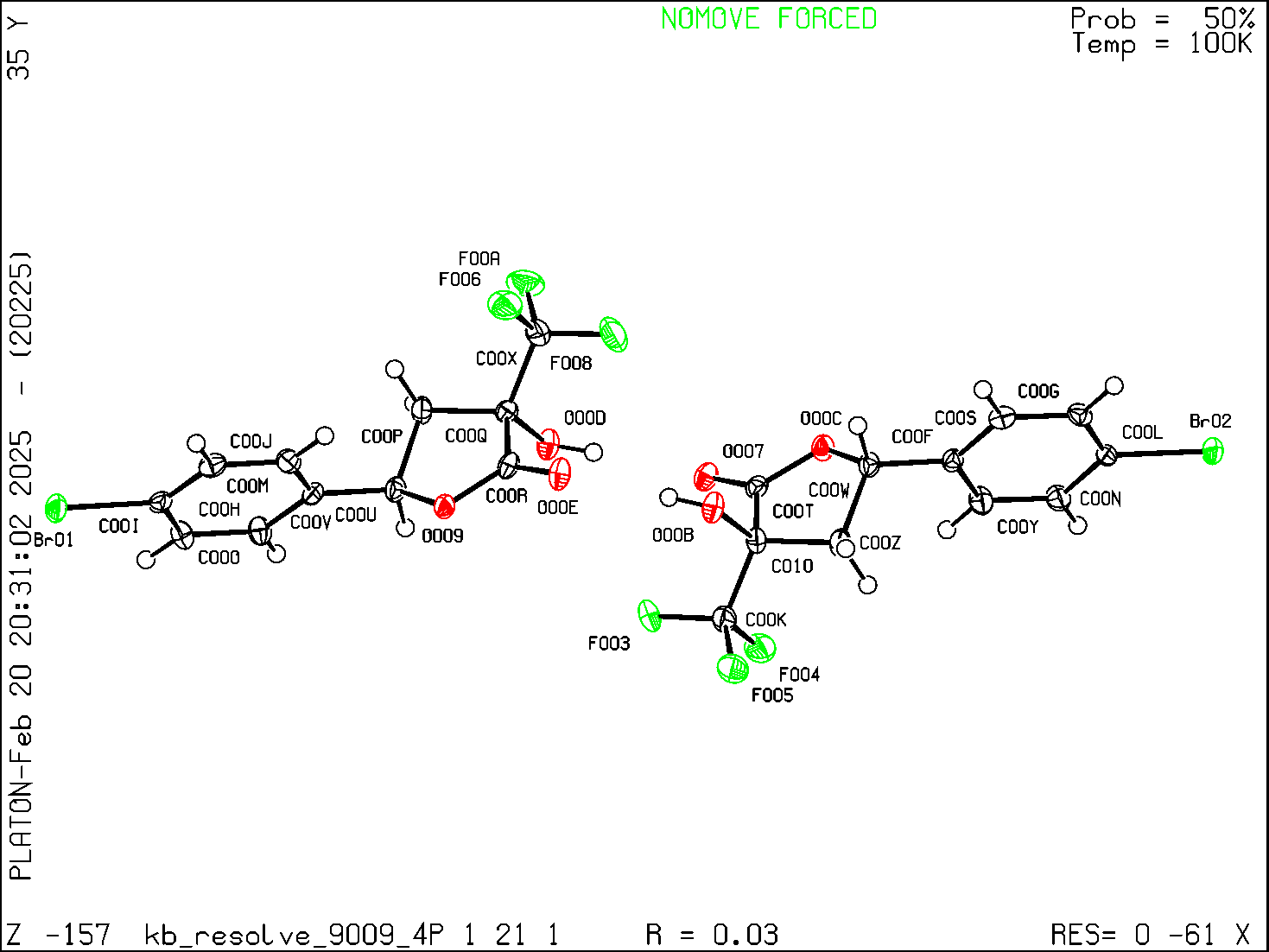


checkCIF/PLATON report

Structure factors have been supplied for datablock(s) kb_resolve_9009_4_a

Datablock: kb_resolve_9009_4_a

Bond precision: C-C = 0.0069 A Wavelength=0.71073

| Cell:  Temperature: | a=8.0112(3)  alpha=90  100 K | | b=14.2016(7) beta=93.550(2) | | c=10.2304(5)  gamma=90 |
| --- | --- | --- | --- | --- | --- |
|  | Calculated |  |  | Reported | |
| Volume Space group Hall group  Moiety formula | 1161.70(9)  P 21  P 2yb  C11 H8 Br | F3 | O3 | 1161.70(9)  P 1 21 1  P 2yb  2(C11 H8 Br F3 O3) | |
| Sum formula | C11 H8 Br | F3 | O3 | C22 H16 Br2 F6 O6 | |
| Mr | 325.07 |  |  | 650.17 | |
| Dx,g cm-3 | 1.859 |  |  | 1.859 | |
| Z | 4 |  |  | 2 | |
| Mu (mm-1) | 3.575 | | 3.575 | | |
| F000 | 640.0 | | 640.0 | | |
| F000’ | 639.32 | |  | | |
| h,k,lmax | 10,17,12 | | 10,17,12 | | |
| Nref | 4761[ 2480] | | 4741 | | |
| Tmin,Tmax | 0.619,0.905 | | 0.448,0.673 | | |
| Tmin’ | 0.272 | |  | | |

Correction method= # Reported T Limits: Tmin=0.448 Tmax=0.673 AbsCorr = MULTI-SCAN

Data completeness= 1.91/1.00 Theta(max)= 26.393

R(reflections)= 0.0294( 4480) wR2(reflections)=

0.0718( 4741)

S = 1.053 Npar= 328

| **Table 1 Crystal data and structure refinement for KB_resolve_9009_4_a.** | |
| --- | --- |
| Identification code | KB_resolve_9009_4_a |
| Empirical formula | C_22_H_16_Br_2_F_6_O_6_ |
| Formula weight | 650.17 |
| Temperature/K | 100.0 |
| Crystal system | monoclinic |
| Space group | P2_1_ |
| a/Å | 8.0112(3) |
| b/Å | 14.2016(7) |
| c/Å | 10.2304(5) |
| α/° | 90 |
| β/° | 93.550(2) |
| γ/° | 90 |
| Volume/Å^3^ | 1161.70(9) |
| Z | 2 |
| ρ_calc_g/cm^3^ | 1.859 |
| μ/mm^‑1^ | 3.575 |
| F(000) | 640.0 |
| Crystal size/mm^3^ | 0.361 × 0.114 × 0.028 |
| Radiation | MoKα (λ = 0.71073) |
| 2Θ range for data collection/° | 3.988 to 52.786 |
| Index ranges | -10 ≤ h ≤ 9, -17 ≤ k ≤ 17, -12 ≤ l ≤ 12 |
| Reflections collected | 28849 |
| Independent reflections | 4741 [R_int_ = 0.0812, R_sigma_ = 0.0525] |
| Data/restraints/parameters | 4741/1/328 |
| Goodness-of-fit on F^2^ | 1.053 |
| Final R indexes [I>=2σ (I)] | R_1_ = 0.0294, wR_2_ = 0.0704 |
| Final R indexes [all data] | R_1_ = 0.0327, wR_2_ = 0.0718 |
| Largest diff. peak/hole / e Å^-3^ | 0.69/-0.32 |
| Flack parameter | 0.318(10) |

| **Table 2 Fractional Atomic Coordinates (×10^4^) and Equivalent Isotropic Displacement Parameters (Å^2^×10^3^) for KB_resolve_9009_4_a. U_eq_ is defined as 1/3 of of the trace of the orthogonalised U_IJ_ tensor.** | | | | |
| --- | --- | --- | --- | --- |
| **Atom** | ***x*** | ***y*** | ***z*** | **U(eq)** |
| Br01 | 12030.1(6) | 3907.7(3) | -520.7(5) | 22.89(14) |
| Br02 | -6913.1(6) | 6357.4(3) | 11317.7(5) | 21.11(13) |
| F003 | 1108(4) | 6174(2) | 3876(3) | 28.5(7) |
| F004 | -1454(4) | 6404(3) | 4328(3) | 29.7(6) |
| F005 | 366(4) | 7521(2) | 4602(3) | 32.2(8) |
| F006 | 6434(4) | 3640(2) | 6380(3) | 33.9(8) |
| O007 | 313(4) | 4580(2) | 5581(4) | 20.3(7) |
| F008 | 3850(4) | 3791(3) | 6806(3) | 37.9(8) |
| O009 | 5620(4) | 4930(2) | 3295(3) | 19.1(7) |
| F00A | 4716(5) | 2482(2) | 6044(4) | 38.0(9) |
| O00B | 2253(4) | 6380(3) | 6461(3) | 20.3(7) |
| O00C | -698(4) | 5125(2) | 7421(3) | 19.0(7) |
| O00D | 2763(4) | 3620(2) | 4206(4) | 21.5(8) |
| O00E | 4656(4) | 5439(2) | 5153(4) | 23.1(8) |
| C00F | -2175(6) | 6085(3) | 8932(5) | 15.7(10) |
| C00G | -3419(6) | 6395(4) | 10968(4) | 17.3(9) |
| C00H | 8567(6) | 3680(3) | -195(5) | 20.2(11) |
| C00I | 10071(6) | 3950(4) | 427(5) | 17.6(9) |
| C00J | 10184(6) | 4255(3) | 1721(5) | 19.2(10) |
| C00K | 129(7) | 6593(3) | 4713(5) | 21.8(11) |
| C00L | -4982(6) | 6252(3) | 10342(5) | 16.2(10) |
| C00M | 8744(6) | 4282(3) | 2404(5) | 18.7(10) |
| C00N | -5173(7) | 6027(4) | 9029(5) | 21.5(11) |
| C00O | 7121(6) | 3724(3) | 511(5) | 19.5(11) |
| C00P | 5525(6) | 3283(3) | 3583(5) | 19.8(10) |
| C00Q | 4462(6) | 3752(3) | 4577(5) | 15.8(10) |
| C00R | 4918(6) | 4797(3) | 4420(5) | 17.8(10) |
| C00S | -2020(6) | 6302(4) | 10246(5) | 17.2(9) |
| C00T | 46(6) | 5231(3) | 6289(5) | 15.5(9) |
| C00U | 5647(6) | 4050(3) | 2550(5) | 17.3(10) |
| C00V | 7208(6) | 4019(3) | 1809(5) | 16.8(10) |
| C00W | -648(6) | 6016(3) | 8156(5) | 16.7(10) |
| C00X | 4870(7) | 3409(4) | 5965(5) | 24.3(11) |
| C00Y | -3760(6) | 5942(4) | 8323(5) | 21.5(11) |
| C00Z | -471(6) | 6767(3) | 7109(5) | 19.4(10) |
| C010 | 537(6) | 6262(3) | 6115(5) | 16.6(9) |

| **Table 3 Anisotropic Displacement Parameters (Å^2^×10^3^) for KB_resolve_9009_4_a. The Anisotropic displacement factor exponent takes the form: -2π^2^[h^2^a*^2^U_11_+2hka*b*U_12_+…].** | | | | | | |
| --- | --- | --- | --- | --- | --- | --- |
| **Atom** | **U_11_** | **U_22_** | **U_33_** | **U_23_** | **U_13_** | **U_12_** |
| Br01 | 18.6(2) | 23.8(2) | 27.3(3) | 2.0(2) | 9.67(18) | 2.7(2) |
| Br02 | 16.4(2) | 24.2(2) | 23.5(3) | 2.3(2) | 7.24(17) | 2.4(2) |
| F003 | 30.9(17) | 36.1(19) | 19.8(16) | -0.3(13) | 12.4(13) | 2.5(13) |
| F004 | 23.9(15) | 39.9(17) | 24.9(15) | 3.5(16) | -2.3(12) | 0.4(16) |
| F005 | 38(2) | 23.1(16) | 36.9(19) | 10.7(14) | 12.4(15) | 0.6(13) |
| F006 | 23.6(16) | 48(2) | 28.8(18) | 8.1(15) | -5.5(13) | -0.2(14) |
| O007 | 19.9(18) | 18.1(17) | 22.9(19) | -6.0(14) | 1.6(14) | 2.3(13) |
| F008 | 30.9(16) | 62(2) | 21.6(16) | 4.6(17) | 9.0(13) | 1.6(18) |
| O009 | 22.3(18) | 14.5(16) | 21.6(19) | 2.2(14) | 8.9(14) | 4.3(13) |
| F00A | 40(2) | 32.5(19) | 41(2) | 20.5(16) | 4.3(16) | -1.4(14) |
| O00B | 14.0(15) | 23.3(16) | 23.8(17) | -8.2(16) | 2.9(13) | -0.8(16) |
| O00C | 21.9(18) | 13.6(16) | 22.3(19) | -0.7(13) | 7.0(14) | 0.9(13) |
| O00D | 14.9(16) | 24.6(18) | 25(2) | -5.7(14) | 4.0(14) | -1.2(13) |
| O00E | 19.9(18) | 23.5(18) | 26(2) | -8.3(15) | 6.2(15) | 1.3(14) |
| C00F | 16(2) | 13(2) | 18(2) | 0.0(17) | 3.4(19) | 1.1(17) |
| C00G | 24(2) | 15(2) | 13(2) | -1(2) | 0.8(17) | 0(2) |
| C00H | 23(2) | 21(3) | 17(3) | -2.8(18) | 6(2) | -2.7(18) |
| C00I | 18(2) | 12(2) | 23(2) | 5(2) | 5(2) | 3(2) |
| C00J | 15(2) | 19(2) | 23(3) | 1(2) | -3(2) | -0.5(18) |
| C00K | 25(2) | 17(3) | 25(3) | 3(2) | 9(2) | 1(2) |
| C00L | 17(2) | 15(2) | 18(2) | 5(2) | 6.4(19) | 1.8(19) |
| C00M | 21(2) | 18(2) | 17(2) | 0.3(19) | 2(2) | 0.0(19) |
| C00N | 17(2) | 29(3) | 17(3) | 1(2) | 0(2) | 0(2) |
| C00O | 19(2) | 22(3) | 18(2) | -4.8(19) | 2.2(19) | -2.4(19) |
| C00P | 20(3) | 18(2) | 23(3) | -1(2) | 9(2) | 1.4(19) |
| C00Q | 13(2) | 18(3) | 17(2) | 0.8(18) | 0.0(17) | 0.6(18) |
| C00R | 9(2) | 21(2) | 23(3) | -2(2) | 0.6(18) | 1.0(18) |
| C00S | 16(2) | 15(2) | 20(2) | 0.1(19) | -2.9(17) | -2(2) |
| C00T | 13(2) | 17(2) | 17(2) | -0.1(19) | 0.0(18) | 1.8(17) |
| C00U | 17(2) | 19(3) | 16(2) | -4(2) | 2.1(18) | 3.0(19) |
| C00V | 16(2) | 15(2) | 20(2) | 1(2) | 3.2(18) | 4.4(19) |
| C00W | 16(2) | 19(2) | 15(2) | -3.5(18) | 1.6(18) | 1.3(18) |
| C00X | 21(3) | 30(3) | 22(3) | 5(2) | 6(2) | 1(2) |
| C00Y | 16(2) | 32(3) | 16(3) | -3(2) | 0(2) | -1(2) |
| C00Z | 20(2) | 15(2) | 25(3) | -1(2) | 9(2) | 0.9(18) |
| C010 | 16(2) | 16(2) | 18(2) | -2(2) | 5.5(18) | -1.0(18) |

| **Table 4 Bond Lengths for KB_resolve_9009_4_a.** | | | | | | |
| --- | --- | --- | --- | --- | --- | --- |
| **Atom** | **Atom** | **Length/Å** |  | **Atom** | **Atom** | **Length/Å** |
| Br01 | C00I | 1.897(5) |  | C00G | C00L | 1.386(7) |
| Br02 | C00L | 1.899(5) |  | C00G | C00S | 1.386(6) |
| F003 | C00K | 1.336(6) |  | C00H | C00I | 1.382(7) |
| F004 | C00K | 1.332(6) |  | C00H | C00O | 1.404(7) |
| F005 | C00K | 1.337(6) |  | C00I | C00J | 1.390(7) |
| F006 | C00X | 1.339(6) |  | C00J | C00M | 1.385(7) |
| O007 | C00T | 1.202(6) |  | C00K | C010 | 1.525(7) |
| F008 | C00X | 1.338(6) |  | C00L | C00N | 1.380(7) |
| O009 | C00R | 1.326(6) |  | C00M | C00V | 1.390(7) |
| O009 | C00U | 1.466(6) |  | C00N | C00Y | 1.386(7) |
| F00A | C00X | 1.326(6) |  | C00O | C00V | 1.390(7) |
| O00B | C010 | 1.408(5) |  | C00P | C00Q | 1.520(7) |
| O00C | C00T | 1.343(6) |  | C00P | C00U | 1.525(7) |
| O00C | C00W | 1.471(6) |  | C00Q | C00R | 1.539(7) |
| O00D | C00Q | 1.403(5) |  | C00Q | C00X | 1.518(7) |
| O00E | C00R | 1.207(6) |  | C00T | C010 | 1.530(7) |
| C00F | C00S | 1.377(7) |  | C00U | C00V | 1.503(6) |
| C00F | C00W | 1.503(7) |  | C00W | C00Z | 1.524(7) |
| C00F | C00Y | 1.394(7) |  | C00Z | C010 | 1.517(6) |

| **Table 5 Bond Angles for KB_resolve_9009_4_a.** | | | | | | | | |
| --- | --- | --- | --- | --- | --- | --- | --- | --- |
| **Atom** | **Atom** | **Atom** | **Angle/˚** |  | **Atom** | **Atom** | **Atom** | **Angle/˚** |
| C00R | O009 | C00U | 110.5(4) |  | O009 | C00R | C00Q | 110.1(4) |
| C00T | O00C | C00W | 110.2(4) |  | O00E | C00R | O009 | 122.1(4) |
| C00S | C00F | C00W | 120.2(4) |  | O00E | C00R | C00Q | 127.7(5) |
| C00S | C00F | C00Y | 119.6(5) |  | C00F | C00S | C00G | 121.0(4) |
| C00Y | C00F | C00W | 120.2(5) |  | O007 | C00T | O00C | 122.5(4) |
| C00L | C00G | C00S | 118.4(4) |  | O007 | C00T | C010 | 127.6(4) |
| C00I | C00H | C00O | 118.3(4) |  | O00C | C00T | C010 | 109.8(4) |
| C00H | C00I | Br01 | 118.8(4) |  | O009 | C00U | C00P | 104.2(4) |
| C00H | C00I | C00J | 121.9(4) |  | O009 | C00U | C00V | 109.0(4) |
| C00J | C00I | Br01 | 119.3(4) |  | C00V | C00U | C00P | 114.9(4) |
| C00M | C00J | C00I | 118.8(5) |  | C00M | C00V | C00O | 119.3(4) |
| F003 | C00K | F005 | 107.0(4) |  | C00M | C00V | C00U | 120.9(4) |
| F003 | C00K | C010 | 111.4(4) |  | C00O | C00V | C00U | 119.8(4) |
| F004 | C00K | F003 | 107.9(4) |  | O00C | C00W | C00F | 109.2(4) |
| F004 | C00K | F005 | 108.1(4) |  | O00C | C00W | C00Z | 104.1(4) |
| F004 | C00K | C010 | 111.2(4) |  | C00F | C00W | C00Z | 116.1(4) |
| F005 | C00K | C010 | 111.1(4) |  | F006 | C00X | C00Q | 111.2(4) |
| C00G | C00L | Br02 | 119.2(4) |  | F008 | C00X | F006 | 107.1(4) |
| C00N | C00L | Br02 | 119.0(4) |  | F008 | C00X | C00Q | 111.5(4) |
| C00N | C00L | C00G | 121.8(4) |  | F00A | C00X | F006 | 108.2(4) |
| C00J | C00M | C00V | 120.9(5) |  | F00A | C00X | F008 | 107.5(4) |
| C00L | C00N | C00Y | 118.8(5) |  | F00A | C00X | C00Q | 111.1(4) |
| C00V | C00O | C00H | 120.7(4) |  | C00N | C00Y | C00F | 120.4(5) |
| C00Q | C00P | C00U | 102.3(4) |  | C010 | C00Z | C00W | 102.6(4) |
| O00D | C00Q | C00P | 109.5(4) |  | O00B | C010 | C00K | 110.4(4) |
| O00D | C00Q | C00R | 109.5(4) |  | O00B | C010 | C00T | 109.7(4) |
| O00D | C00Q | C00X | 111.1(4) |  | O00B | C010 | C00Z | 109.1(4) |
| C00P | C00Q | C00R | 101.9(4) |  | C00K | C010 | C00T | 111.3(4) |
| C00X | C00Q | C00P | 112.9(4) |  | C00Z | C010 | C00K | 113.2(4) |
| C00X | C00Q | C00R | 111.6(4) |  | C00Z | C010 | C00T | 102.9(4) |

| **Table 6 Torsion Angles for KB_resolve_9009_4_a.** | | | | | | | | | | |
| --- | --- | --- | --- | --- | --- | --- | --- | --- | --- | --- |
| **A** | **B** | **C** | **D** | **Angle/˚** |  | **A** | **B** | **C** | **D** | **Angle/˚** |
| Br01 | C00I | C00J | C00M | -180.0(4) |  | C00P | C00Q | C00R | O009 | 17.7(5) |
| Br02 | C00L | C00N | C00Y | -179.4(4) |  | C00P | C00Q | C00R | O00E | -164.7(5) |
| F003 | C00K | C010 | O00B | 53.7(5) |  | C00P | C00Q | C00X | F006 | 64.3(6) |
| F003 | C00K | C010 | C00T | -68.4(5) |  | C00P | C00Q | C00X | F008 | -176.3(4) |
| F003 | C00K | C010 | C00Z | 176.4(4) |  | C00P | C00Q | C00X | F00A | -56.3(5) |
| F004 | C00K | C010 | O00B | 174.1(4) |  | C00P | C00U | C00V | C00M | -72.4(6) |
| F004 | C00K | C010 | C00T | 51.9(5) |  | C00P | C00U | C00V | C00O | 107.5(5) |
| F004 | C00K | C010 | C00Z | -63.3(5) |  | C00Q | C00P | C00U | O009 | 32.2(5) |
| F005 | C00K | C010 | O00B | -65.5(5) |  | C00Q | C00P | C00U | C00V | 151.4(4) |
| F005 | C00K | C010 | C00T | 172.4(4) |  | C00R | O009 | C00U | C00P | -22.5(5) |
| F005 | C00K | C010 | C00Z | 57.2(6) |  | C00R | O009 | C00U | C00V | -145.6(4) |
| O007 | C00T | C010 | O00B | -76.8(6) |  | C00R | C00Q | C00X | F006 | -49.8(6) |
| O007 | C00T | C010 | C00K | 45.8(7) |  | C00R | C00Q | C00X | F008 | 69.6(5) |
| O007 | C00T | C010 | C00Z | 167.2(5) |  | C00R | C00Q | C00X | F00A | -170.4(4) |
| O009 | C00U | C00V | C00M | 44.1(6) |  | C00S | C00F | C00W | O00C | 129.9(4) |
| O009 | C00U | C00V | C00O | -136.1(5) |  | C00S | C00F | C00W | C00Z | -112.9(5) |
| O00C | C00T | C010 | O00B | 100.5(4) |  | C00S | C00F | C00Y | C00N | 0.6(7) |
| O00C | C00T | C010 | C00K | -137.0(4) |  | C00S | C00G | C00L | Br02 | 179.1(4) |
| O00C | C00T | C010 | C00Z | -15.6(5) |  | C00S | C00G | C00L | C00N | -0.3(8) |
| O00C | C00W | C00Z | C010 | -32.0(5) |  | C00T | O00C | C00W | C00F | 148.3(4) |
| O00D | C00Q | C00R | O009 | -98.2(4) |  | C00T | O00C | C00W | C00Z | 23.7(5) |
| O00D | C00Q | C00R | O00E | 79.3(6) |  | C00U | O009 | C00R | O00E | -174.8(4) |
| O00D | C00Q | C00X | F006 | -172.3(4) |  | C00U | O009 | C00R | C00Q | 2.9(5) |
| O00D | C00Q | C00X | F008 | -52.8(6) |  | C00U | C00P | C00Q | O00D | 86.2(4) |
| O00D | C00Q | C00X | F00A | 67.1(5) |  | C00U | C00P | C00Q | C00R | -29.7(5) |
| C00F | C00W | C00Z | C010 | -152.1(4) |  | C00U | C00P | C00Q | C00X | -149.5(4) |
| C00G | C00L | C00N | C00Y | -0.1(8) |  | C00W | O00C | C00T | O007 | 172.3(4) |
| C00H | C00I | C00J | C00M | 0.4(8) |  | C00W | O00C | C00T | C010 | -5.1(5) |
| C00H | C00O | C00V | C00M | 0.6(7) |  | C00W | C00F | C00S | C00G | 178.1(5) |
| C00H | C00O | C00V | C00U | -179.2(4) |  | C00W | C00F | C00Y | C00N | -178.5(5) |
| C00I | C00H | C00O | C00V | -0.9(7) |  | C00W | C00Z | C010 | O00B | -87.9(5) |
| C00I | C00J | C00M | C00V | -0.7(7) |  | C00W | C00Z | C010 | C00K | 148.7(4) |
| C00J | C00M | C00V | C00O | 0.2(7) |  | C00W | C00Z | C010 | C00T | 28.5(5) |
| C00J | C00M | C00V | C00U | -180.0(4) |  | C00X | C00Q | C00R | O009 | 138.4(4) |
| C00L | C00G | C00S | C00F | 0.9(8) |  | C00X | C00Q | C00R | O00E | -44.0(7) |
| C00L | C00N | C00Y | C00F | -0.1(8) |  | C00Y | C00F | C00S | C00G | -1.0(7) |
| C00O | C00H | C00I | Br01 | -179.3(4) |  | C00Y | C00F | C00W | O00C | -50.9(6) |
| C00O | C00H | C00I | C00J | 0.4(8) |  | C00Y | C00F | C00W | C00Z | 66.3(6) |

| **Table 7 Hydrogen Atom Coordinates (Å×10^4^) and Isotropic Displacement Parameters (Å^2^×10^3^) for KB_resolve_9009_4_a.** | | | | |
| --- | --- | --- | --- | --- |
| **Atom** | ***x*** | ***y*** | ***z*** | **U(eq)** |
| H00C | 2809.41 | 6009.22 | 6018.24 | 30 |
| H00D | 2179.9 | 3957.18 | 4675.81 | 32 |
| H00G | -3308.35 | 6552.89 | 11871.94 | 21 |
| H00H | 8512.22 | 3469.94 | -1077.94 | 24 |
| H00J | 11228.95 | 4441.15 | 2129.59 | 23 |
| H00M | 8807.96 | 4482.42 | 3291.23 | 22 |
| H00N | -6255.33 | 5930.81 | 8616.93 | 26 |
| H00O | 6071.63 | 3550.1 | 97.31 | 23 |
| H00A | 4972.87 | 2711.47 | 3208.13 | 24 |
| H00B | 6642.58 | 3112.42 | 3978.91 | 24 |
| H00S | -938.75 | 6388.31 | 10663.55 | 21 |
| H00U | 4643.12 | 4020.24 | 1919.13 | 21 |
| H00W | 372.2 | 6023.42 | 8770.88 | 20 |
| H00Y | -3871.19 | 5786.55 | 7418.69 | 26 |
| H00E | -1577.08 | 6963.91 | 6718.05 | 23 |
| H00F | 132.02 | 7325.95 | 7472.32 | 23 |

**Crystal structure determination of [KB_resolve_9009_4_a]**

**Crystal Data** for C_22_H_16_Br_2_F_6_O_6_ (*M*=650.17 g/mol): monoclinic, space group P2_1_ (no. 4), *a* = 8.0112(3) Å, *b* = 14.2016(7) Å, *c* = 10.2304(5) Å, *β* = 93.550(2)°, *V*= 1161.70(9) Å^3^, *Z* = 2, *T* = 100.0 K, μ(MoKα) = 3.575 mm^-1^, *Dcalc* = 1.859 g/cm^3^, 28849 reflections measured (3.988° ≤ 2Θ ≤ 52.786°), 4741 unique (*R*_int_ = 0.0812, R_sigma_ = 0.0525) which were used in all calculations. The final *R*_1_ was 0.0294 (I > 2σ(I)) and *wR*_2_ was 0.0718 (all data).

Crystal Data for minor diastereomer CCDC **2425834** (**9b**)

**Datablock 8071_final_resolve_a** - ellipsoid plot
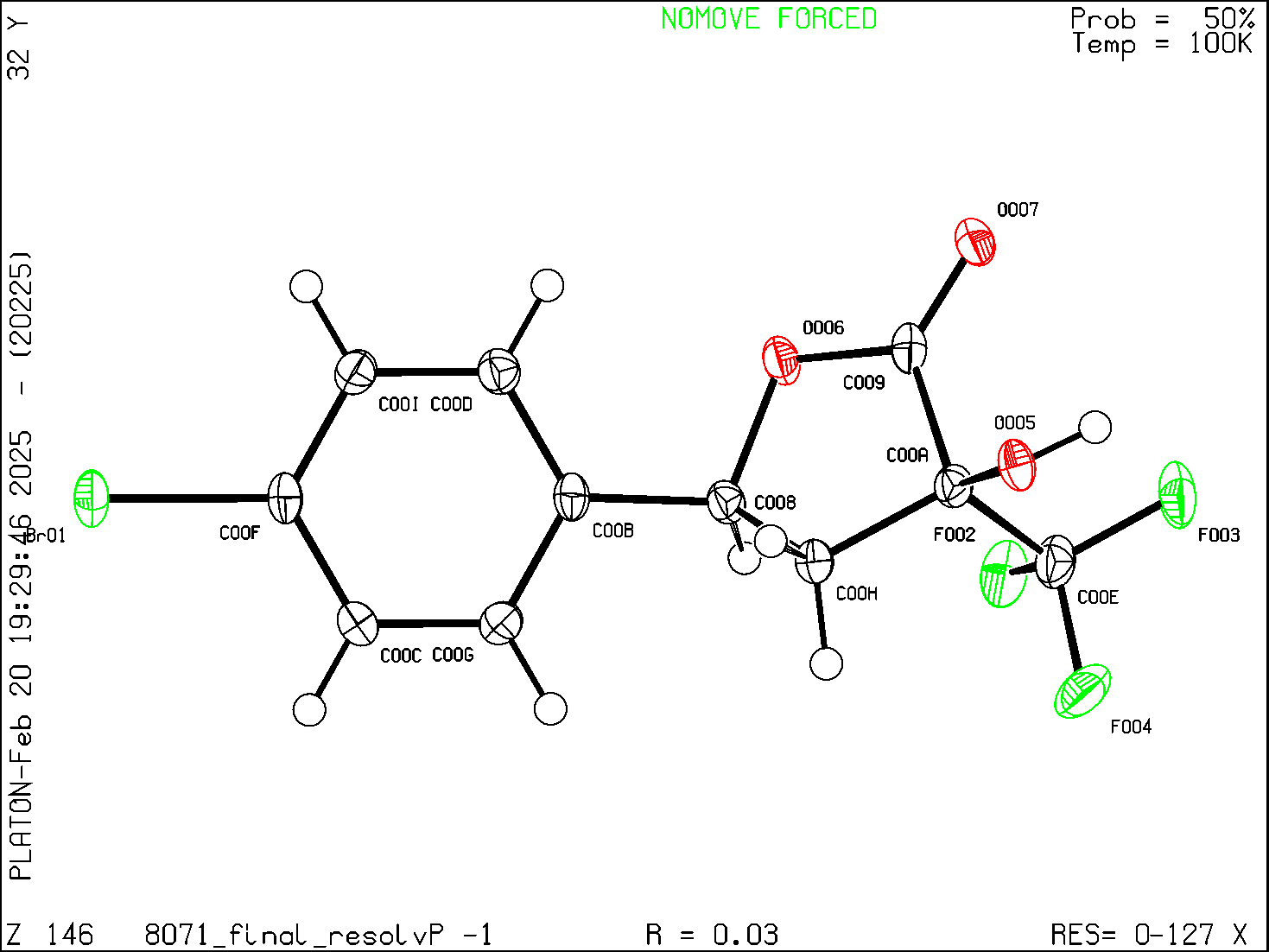


checkCIF/PLATON report

Structure factors have been supplied for datablock(s) 8071_final_resolve_a

Datablock: 8071_final_resolve_a

Bond precision: C-C = 0.0023 A Wavelength=0.71073

| Cell:  Temperature: | a=7.0542(2)  alpha=84.516(2)  100 K | | | b=8.8396(3) beta=70.939(2) | c=9.7383(3)  gamma=80.560(2) |
| --- | --- | --- | --- | --- | --- |
|  | Calculated |  |  | Reported | |
| Volume Space group Hall group  Moiety formula | 565.60(3)  P -1  -P 1  C11 H8 Br | F3 | O3 | 565.60(3)  P -1  -P 1  C11 H8 Br F3 O3 | |
| Sum formula | C11 H8 Br | F3 | O3 | C11 H8 Br F3 O3 | |
| Mr | 325.07 |  |  | 325.08 | |
| Dx,g cm-3 | 1.909 |  |  | 1.909 | |
| Z | 2 |  |  | 2 | |
| Mu (mm-1) | 3.672 | | 3.672 | | |
| F000 | 320.0 | | 320.0 | | |
| F000’ | 319.66 | |  | | |
| h,k,lmax | 8,11,12 | | 8,11,12 | | |
| Nref | 2312 | | 2302 | | |
| Tmin,Tmax | 0.522,0.832 | | 0.555,0.745 | | |
| Tmin’ | 0.236 | |  | | |

Correction method= # Reported T Limits: Tmin=0.555 Tmax=0.745 AbsCorr = MULTI-SCAN

Data completeness= 0.996 Theta(max)= 26.408

R(reflections)= 0.0298( 2109) wR2(reflections)=

0.0639( 2302)

S = 1.134 Npar= 152

 block-like specimen of C_11_H_4_BrF_4_O_2_ was used for the X-ray crystallographic analysis. The X-ray intensity data were measured (λ = 0.71073 Å).

| **Table 1: Data collection details for 8071.** |
| --- |

| **Axis** | **dx/mm** | **2θ/°** | **ω/°** | **φ/°** | **χ/°** | **Width/°** | **Frames** | **Time/s** | **Wavelength/Å** | **Voltage/kV** | **Current/mA** | **Temperature/K** |
| --- | --- | --- | --- | --- | --- | --- | --- | --- | --- | --- | --- | --- |
| Phi | 38.018 | 0.00 | 360.00 | 0.00 | 54.74 | 1.00 | 180 | 1.00 | 0.71073 | 50 | 1.4 | 100 |
| Phi | 38.018 | 0.00 | 360.00 | 180.00 | 54.74 | 1.00 | 180 | 1.00 | 0.71073 | 50 | 1.4 | 100 |
| Omega | 38.018 | 36.11 | 324.16 | 204.00 | 66.50 | 2.00 | 39 | 4.00 | 0.71073 | 50 | 1.4 | 100 |
| Omega | 38.017 | 36.11 | 135.67 | 153.00 | -44.50 | -2.00 | 50 | 4.00 | 0.71073 | 50 | 1.4 | 100 |
| Omega | 38.017 | 36.11 | 35.67 | 0.00 | -44.50 | 2.00 | 50 | 4.00 | 0.71073 | 50 | 1.4 | 100 |
| Omega | 38.019 | 36.11 | 324.16 | 360.00 | 66.50 | 2.00 | 39 | 4.00 | 0.71073 | 50 | 1.4 | 100 |
| Omega | 38.018 | 36.11 | 135.67 | 255.00 | -44.50 | -2.00 | 50 | 4.00 | 0.71073 | 50 | 1.4 | 100 |
| Omega | 38.017 | 36.11 | 324.16 | 102.00 | 66.50 | 2.00 | 39 | 4.00 | 0.71073 | 50 | 1.4 | 100 |
| Omega | 38.017 | 36.11 | 35.67 | 51.00 | -44.50 | 2.00 | 50 | 4.00 | 0.71073 | 50 | 1.4 | 100 |
| Omega | 38.018 | 36.11 | 324.16 | 306.00 | 66.50 | 2.00 | 39 | 4.00 | 0.71073 | 50 | 1.4 | 100 |
| Omega | 38.018 | 36.11 | 135.67 | 204.00 | -44.50 | -2.00 | 50 | 4.00 | 0.71073 | 50 | 1.4 | 101 |
| Omega | 38.018 | 36.11 | 135.67 | 306.00 | -44.50 | -2.00 | 50 | 4.00 | 0.71073 | 50 | 1.4 | 99 |
| Omega | 38.018 | 36.11 | 135.67 | 102.00 | -44.50 | -2.00 | 50 | 4.00 | 0.71073 | 50 | 1.4 | 100 |
| Omega | 38.017 | 36.11 | 324.16 | 153.00 | 66.50 | 2.00 | 39 | 4.00 | 0.71073 | 50 | 1.4 | 100 |
| Omega | 38.017 | 36.11 | 324.16 | 51.00 | 66.50 | 2.00 | 39 | 4.00 | 0.71073 | 50 | 1.4 | 100 |
| Omega | 38.017 | 36.11 | 324.16 | 255.00 | 66.50 | 2.00 | 39 | 4.00 | 0.71073 | 50 | 1.4 | 100 |
| Omega | 38.018 | 21.11 | 20.85 | 0.00 | -44.50 | 2.00 | 52 | 4.00 | 0.71073 | 50 | 1.4 | 100 |
| Omega | 38.018 | 21.11 | 20.85 | 180.00 | -44.50 | 2.00 | 52 | 4.00 | 0.71073 | 50 | 1.4 | 100 |
| Omega | 38.018 | 21.11 | 20.85 | 90.00 | -44.50 | 2.00 | 52 | 4.00 | 0.71073 | 50 | 1.4 | 100 |
| Phi | 38.018 | 36.11 | 38.81 | 0.00 | -24.00 | 2.00 | 180 | 4.00 | 0.71073 | 50 | 1.4 | 100 |
| Phi | 38.018 | 0.00 | 0.00 | 0.00 | 54.70 | -1.00 | 180 | 1.00 | 0.71073 | 50 | 1.4 | 100 |

A total of 1499 frames were collected. The total exposure time was 1.22 hours. The frames were integrated with the Bruker SAINT software package using a narrow-frame algorithm. The integration of the data using a triclinic unit cell yielded a total of 31397 reflections to a maximum θ angle of 26.40° (0.80 Å resolution), of which 2813 were independent (average redundancy 11.161, completeness = 100.0%, R_int_ = 10.74%, R_sig_ = 4.47%) and 2550 (90.65%) were greater than 2σ(F^2^). The final cell constants of a = 7.0542(3) Å, b = 8.8398(3) Å, c = 9.7389(4) Å, α = 84.5151(19)°, β = 70.9449(18)°, γ = 80.5558(18)°, volume = 565.67(6) Å^3^, are based upon the refinement of the XYZ-centroids of 8977 reflections above 20 σ(I) with 6.172° < 2θ < 52.73°. Data were corrected for absorption effects using the Multi-Scan method (SADABS). The ratio of minimum to maximum apparent transmission was 0.745.

The structure was solved and refined using the Bruker SHELXTL Software Package, using the space group P -1, with Z = 2 for the formula unit, C_11_H_4_BrF_4_O_2_. The final anisotropic full-matrix least-squares refinement on F^2^ with 163 variables converged at R1 = 4.39%, for the observed data and wR2 = 12.09% for all data. The goodness-of-fit was 1.278. The largest peak in the final difference electron density synthesis was 1.331 e^-^/Å^3^ and the largest hole was -1.142 e^-^/Å^3^ with an RMS deviation of 0.301 e^-^/Å^3^. On the basis of the final model, the calculated density was 1.903 g/cm^3^ and F(000), 314 e^-^.

|  |  |  |  |  |
| --- | --- | --- | --- | --- |
|  | | |  |  |
| **Table 2. Sample and crystal data for 8071.** | | | | |

| **Identification code** | 8071 | |
| --- | --- | --- |
| **Chemical formula** | C_11_H_4_BrF_4_O_2_ | |
| **Formula weight** | 324.05 g/mol | |
| **Temperature** | 100(2) K | |
| **Wavelength** | 0.71073 Å | |
| **Crystal system** | triclinic | |
| **Space group** | P -1 | |
| **Unit cell dimensions** | a = 7.0542(3) Å | α = 84.5151(19)° |
|  | b = 8.8398(3) Å | β = 70.9449(18)° |
|  | c = 9.7389(4) Å | γ = 80.5558(18)° |
| **Volume** | 565.67(6) Å^3^ |  |
| **Z** | 2 | |
| **Density (calculated)** | 1.903 g/cm^3^ | |
| **Absorption coefficient** | 3.677 mm^-1^ | |
| **F(000)** | 314 | |

| **Table 3. Data collection and structure refinement for 8071.** |
| --- |

| **Theta range for data collection** | 2.21 to 26.40° | |
| --- | --- | --- |
| **Index ranges** | -9<=h<=9, -11<=k<=11, -12<=l<=12 | |
| **Reflections collected** | 31397 | |
| **Independent reflections** | 2813 [R(int) = 0.1074] | |
| **Coverage of independent reflections** | 100.0% | |
| **Absorption correction** | Multi-Scan | |
| **Structure solution technique** | direct methods | |
| **Structure solution program** | XT, VERSION 2018/2 | |
| **Refinement method** | Full-matrix least-squares on F^2^ | |
| **Refinement program** | SHELXL-2019/1 (Sheldrick, 2019) | |
| **Function minimized** | Σ w(F_o_^2^ - F_c_^2^)^2^ | |
| **Data / restraints / parameters** | 2813 / 0 / 163 | |
| **Goodness-of-fit on F^2^** | 1.278 | |
| **Final R indices** | 2550 data; I>2σ(I) | R1 = 0.0439, wR2 = 0.1181 |
|  | all data | R1 = 0.0482, wR2 = 0.1209 |
| **Weighting scheme** | w=1/[σ^2^(F_o_^2^)+(0.0679P)^2^+0.1384P] where P=(F_o_^2^+2F_c_^2^)/3 | |
| **Largest diff. peak and hole** | 1.331 and -1.142 eÅ^-3^ | |
| **R.M.S. deviation from mean** | 0.301 eÅ^-3^ | |

| **Table 4. Atomic coordinates and equivalent isotropic atomic displacement parameters (Å^2^) for 8071.** |
| --- |
| U(eq) is defined as one third of the trace of the orthogonalized U_ij_ tensor. |
|  |

|  | **x/a** | **y/b** | **z/c** | **U(eq)** |
| --- | --- | --- | --- | --- |
| Br1 | 0.82235(4) | 0.24890(4) | 0.69133(3) | 0.02225(14) |
| F1 | 0.0384(3) | 0.2390(2) | 0.9495(2) | 0.0299(5) |
| F2 | 0.2997(3) | 0.4348(2) | 0.9588(2) | 0.0301(5) |
| F3 | 0.3447(4) | 0.1224(2) | 0.8894(2) | 0.0318(5) |
| F4 | 0.1336(3) | 0.0737(2) | 0.0994(2) | 0.0276(4) |
| O1 | 0.1392(3) | 0.3037(3) | 0.3200(2) | 0.0193(4) |
| O2 | 0.9342(3) | 0.4485(3) | 0.2110(2) | 0.0216(5) |
| C1 | 0.1929(5) | 0.1862(4) | 0.9996(3) | 0.0214(6) |
| C2 | 0.2578(4) | 0.3142(3) | 0.0628(3) | 0.0156(5) |
| C3 | 0.4365(5) | 0.2571(4) | 0.1192(3) | 0.0170(6) |
| C4 | 0.3349(4) | 0.2046(3) | 0.2774(3) | 0.0159(5) |
| C5 | 0.4491(4) | 0.2187(3) | 0.3805(3) | 0.0157(5) |
| C6 | 0.3739(5) | 0.3191(4) | 0.4940(3) | 0.0178(6) |
| C7 | 0.4844(5) | 0.3269(3) | 0.5876(3) | 0.0183(6) |
| C8 | 0.6691(4) | 0.2352(3) | 0.5658(3) | 0.0163(5) |
| C9 | 0.0893(5) | 0.3642(3) | 0.2030(3) | 0.0166(6) |
| C10 | 0.6341(5) | 0.1258(3) | 0.3622(3) | 0.0179(6) |
| C11 | 0.7460(5) | 0.1338(3) | 0.4532(3) | 0.0180(6) |

| **Table 5. Bond lengths (Å) for 8071.** |
| --- |

| Br1-C8 | 1.900(3) | F1-C1 | 1.334(4) |
| --- | --- | --- | --- |
| F2-C2 | 1.396(3) | F3-C1 | 1.336(4) |
| F4-C1 | 1.340(4) | O1-C9 | 1.339(3) |
| O1-C4 | 1.464(3) | O2-C9 | 1.203(4) |
| C1-C2 | 1.535(4) | C2-C3 | 1.529(4) |
| C2-C9 | 1.535(4) | C3-C4 | 1.534(4) |
| C4-C5 | 1.503(4) | C5-C10 | 1.390(4) |
| C5-C6 | 1.393(4) | C6-C7 | 1.392(4) |
| C6-H3 | 0.930000 | C7-C8 | 1.381(4) |
| C7-H4 | 0.930000 | C8-C11 | 1.393(4) |
| C10-C11 | 1.378(4) | C10-H2 | 0.930000 |
| C11-H1 | 0.930000 |  |  |

| **Table 6. Bond angles (°) for 8071.** |
| --- |

| C9-O1-C4 | 111.0(2) | F1-C1-F3 | 107.4(3) |
| --- | --- | --- | --- |
| F1-C1-F4 | 107.2(3) | F3-C1-F4 | 107.2(3) |
| F1-C1-C2 | 111.6(3) | F3-C1-C2 | 111.4(3) |
| F4-C1-C2 | 111.8(2) | F2-C2-C3 | 111.6(2) |
| F2-C2-C1 | 109.5(2) | C3-C2-C1 | 112.7(2) |
| F2-C2-C9 | 112.7(2) | C3-C2-C9 | 101.7(2) |
| C1-C2-C9 | 108.5(2) | C2-C3-C4 | 103.5(2) |
| O1-C4-C5 | 110.0(2) | O1-C4-C3 | 104.0(2) |
| C5-C4-C3 | 115.2(2) | C10-C5-C6 | 119.6(3) |
| C10-C5-C4 | 118.2(3) | C6-C5-C4 | 122.2(3) |
| C7-C6-C5 | 120.0(3) | C7-C6-H3 | 120.000000 |
| C5-C6-H3 | 120.000000 | C8-C7-C6 | 119.2(3) |
| C8-C7-H4 | 120.400000 | C6-C7-H4 | 120.400000 |
| C7-C8-C11 | 121.4(3) | C7-C8-Br1 | 119.2(2) |
| C11-C8-Br1 | 119.4(2) | O2-C9-O1 | 123.0(3) |
| O2-C9-C2 | 126.4(3) | O1-C9-C2 | 110.6(2) |
| C11-C10-C5 | 120.9(3) | C11-C10-H2 | 119.600000 |
| C5-C10-H2 | 119.600000 | C10-C11-C8 | 118.8(3) |
| C10-C11-H1 | 120.600000 | C8-C11-H1 | 120.600000 |

| **Table 7. Torsion angles (°) for 8071.** |
| --- |

| F1-C1-C2-F2 | -54.5(3) | F3-C1-C2-F2 | 65.6(3) |
| --- | --- | --- | --- |
| F4-C1-C2-F2 | -174.5(2) | F1-C1-C2-C3 | -179.3(2) |
| F3-C1-C2-C3 | -59.2(3) | F4-C1-C2-C3 | 60.7(3) |
| F1-C1-C2-C9 | 68.9(3) | F3-C1-C2-C9 | -171.0(2) |
| F4-C1-C2-C9 | -51.1(3) | F2-C2-C3-C4 | 148.0(2) |
| C1-C2-C3-C4 | -88.4(3) | C9-C2-C3-C4 | 27.6(3) |
| C9-O1-C4-C5 | 144.0(2) | C9-O1-C4-C3 | 20.2(3) |
| C2-C3-C4-O1 | -29.6(3) | C2-C3-C4-C5 | -150.0(3) |
| O1-C4-C5-C10 | 176.3(2) | C3-C4-C5-C10 | -66.6(3) |
| O1-C4-C5-C6 | -2.6(4) | C3-C4-C5-C6 | 114.5(3) |
| C10-C5-C6-C7 | 0.5(4) | C4-C5-C6-C7 | 179.4(3) |
| C5-C6-C7-C8 | 0.5(4) | C6-C7-C8-C11 | -0.7(4) |
| C6-C7-C8-Br1 | 178.8(2) | C4-O1-C9-O2 | 179.4(3) |
| C4-O1-C9-C2 | -2.1(3) | F2-C2-C9-O2 | 42.0(4) |
| C3-C2-C9-O2 | 161.6(3) | C1-C2-C9-O2 | -79.4(4) |
| F2-C2-C9-O1 | -136.4(3) | C3-C2-C9-O1 | -16.8(3) |
| C1-C2-C9-O1 | 102.2(3) | C6-C5-C10-C11 | -1.2(4) |
| C4-C5-C10-C11 | 179.9(3) | C5-C10-C11-C8 | 1.0(4) |
| C7-C8-C11-C10 | 0.0(4) | Br1-C8-C11-C10 | -179.5(2) |

| **Table 8. Anisotropic atomic displacement parameters (Å^2^) for 8071.** |
| --- |
| The anisotropic atomic displacement factor exponent takes the form: -2π^2^[ h^2^ a^*2^ U_11_ + ... + 2 h k a^*^ b^*^ U_12_ ] |

|  | **U_11_** | **U_22_** | **U_33_** | **U_23_** | **U_13_** | **U_12_** |
| --- | --- | --- | --- | --- | --- | --- |
| Br1 | 0.02343(19) | 0.0301(2) | 0.01889(19) | 0.00030(12) | -0.01391(13) | -0.00540(13) |
| F1 | 0.0415(12) | 0.0254(10) | 0.0372(11) | 0.0017(8) | -0.0314(10) | -0.0078(9) |
| F2 | 0.0331(11) | 0.0310(11) | 0.0277(10) | 0.0049(8) | -0.0123(9) | -0.0066(9) |
| F3 | 0.0476(13) | 0.0268(11) | 0.0229(10) | -0.0108(8) | -0.0122(9) | -0.0026(9) |
| F4 | 0.0447(12) | 0.0199(9) | 0.0269(10) | 0.0062(7) | -0.0210(9) | -0.0134(8) |
| O1 | 0.0156(10) | 0.0264(11) | 0.0166(10) | 0.0020(8) | -0.0084(8) | 0.0000(8) |
| O2 | 0.0188(10) | 0.0283(12) | 0.0185(10) | -0.0004(9) | -0.0104(8) | 0.0030(9) |
| C1 | 0.0325(17) | 0.0170(14) | 0.0201(14) | 0.0006(11) | -0.0163(13) | -0.0026(12) |
| C2 | 0.0202(14) | 0.0142(13) | 0.0146(13) | -0.0004(10) | -0.0088(11) | -0.0016(10) |
| C3 | 0.0189(13) | 0.0203(14) | 0.0129(12) | 0.0001(10) | -0.0085(10) | 0.0007(11) |
| C4 | 0.0170(13) | 0.0156(13) | 0.0172(13) | 0.0005(10) | -0.0099(11) | 0.0002(10) |
| C5 | 0.0191(13) | 0.0163(14) | 0.0144(12) | 0.0041(10) | -0.0094(10) | -0.0044(11) |
| C6 | 0.0182(13) | 0.0200(14) | 0.0160(13) | 0.0006(11) | -0.0074(11) | -0.0014(11) |
| C7 | 0.0215(14) | 0.0196(14) | 0.0158(13) | -0.0022(11) | -0.0086(11) | -0.0024(11) |
| C8 | 0.0189(13) | 0.0197(14) | 0.0139(12) | 0.0025(10) | -0.0097(10) | -0.0051(11) |
| C9 | 0.0201(14) | 0.0174(13) | 0.0158(13) | 0.0011(10) | -0.0099(11) | -0.0055(11) |
| C10 | 0.0205(14) | 0.0179(14) | 0.0162(13) | -0.0022(10) | -0.0076(11) | -0.0004(11) |
| C11 | 0.0168(13) | 0.0198(14) | 0.0189(14) | -0.0003(11) | -0.0090(11) | 0.0000(11) |

| **Table 9. Hydrogen atomic coordinates and isotropic atomic displacement parameters (Å^2^) for 8071.** |
| --- |

|  | **x/a** | **y/b** | **z/c** | **U(eq)** |
| --- | --- | --- | --- | --- |
| H3 | 0.2498 | 0.3809 | 0.5073 | 0.021000 |
| H4 | 0.4344 | 0.3932 | 0.6640 | 0.022000 |
| H2 | 0.6830 | 0.0574 | 0.2876 | 0.021000 |
| H1 | 0.8706 | 0.0725 | 0.4396 | 0.022000 |

Crystal Data for CCDC **2425657** (X)

**Datablock solve2_a_pl** - ellipsoid plot
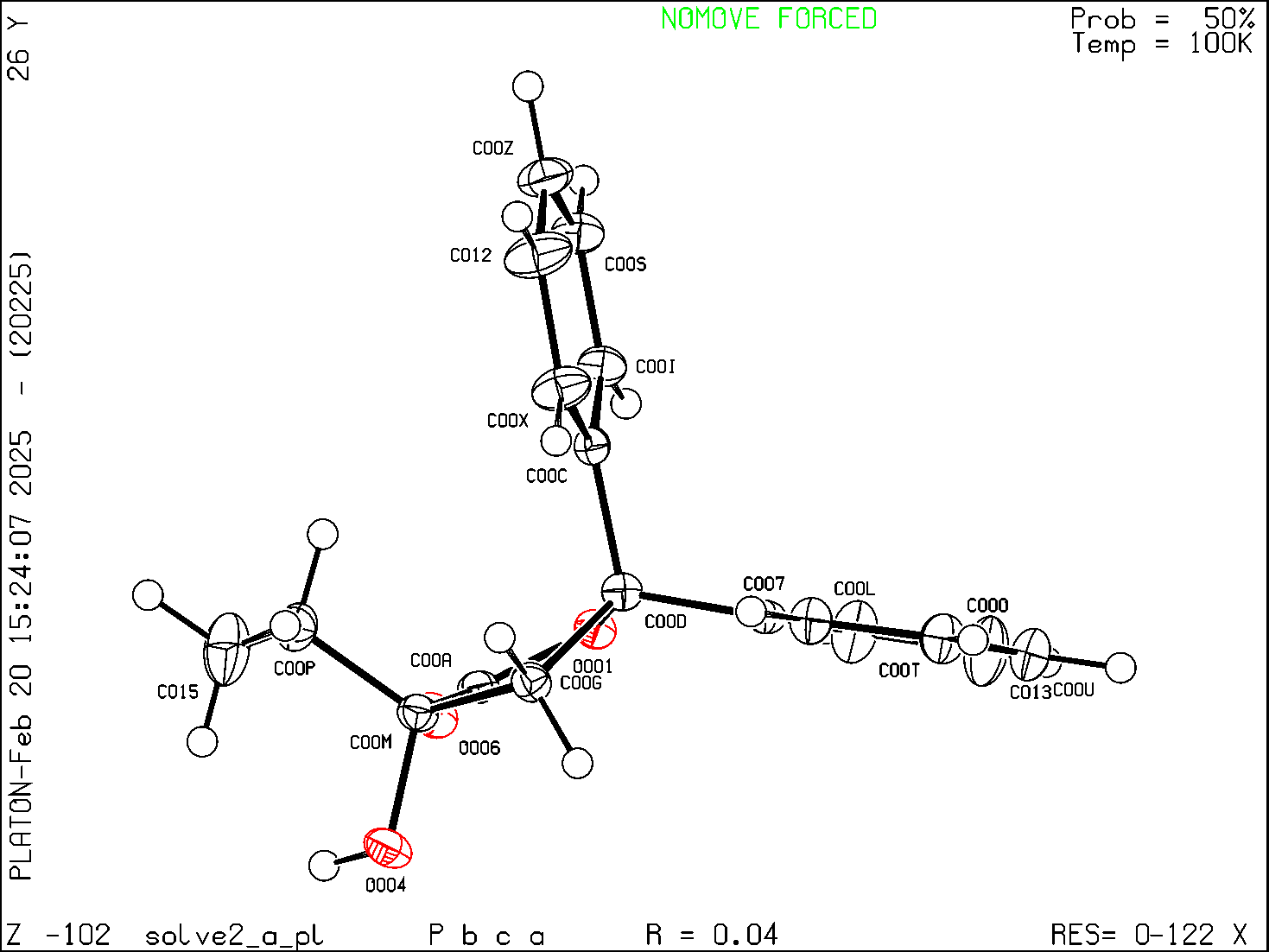


checkCIF/PLATON report

Datablock: solve2_a_pl

Bond precision: C-C = 0.0016 A Wavelength=1.54178

| Cell:  Temperature: | a=8.3214(7)  alpha=90  100 K | b=16.3296(13)  beta=90 | c=21.9255(17)  gamma=90 |
| --- | --- | --- | --- |
|  | Calculated | Reported | |
| Volume | 2979.4(4) | 2979.3(4) | |
| Space group | P b c a | P b c a | |
| Hall group | -P 2ac 2ab | -P 2ac 2ab | |
| Moiety formula | C18 H18 O3 | C18 H18 O3 | |
| Sum formula | C18 H18 O3 | C18 H18 O3 | |
| Mr | 282.32 | 282.32 | |
| Dx,g cm-3 | 1.259 | 1.259 | |
| Z | 8 | 8 | |
| Mu (mm-1) | 0.683 | 0.683 | |
| F000 | 1200.0 | 1200.0 | |
| F000’ | 1203.65 |  | |
| h,k,lmax | 10,20,27 | 10,20,27 | |
| Nref | 3067 | 2986 | |
| Tmin,Tmax | 0.899,0.966 | 0.629,0.754 | |
| Tmin’ | 0.761 |  | |

Correction method= # Reported T Limits: Tmin=0.629 Tmax=0.754 AbsCorr = MULTI-SCAN

Data completeness= 0.974 Theta(max)= 74.769

R(reflections)= 0.0374( 2823) wR2(reflections)=

0.0912( 2986)

S = 1.027 Npar= 192

| **Table 1 Crystal data and structure refinement for solve2_a_pl.** | |
| --- | --- |
| Identification code | solve2_a_pl |
| Empirical formula | C_18_H_18_O_3_ |
| Formula weight | 282.32 |
| Temperature/K | 100.15 |
| Crystal system | orthorhombic |
| Space group | Pbca |
| a/Å | 8.3214(7) |
| b/Å | 16.3296(13) |
| c/Å | 21.9255(17) |
| α/° | 90 |
| β/° | 90 |
| γ/° | 90 |
| Volume/Å^3^ | 2979.3(4) |
| Z | 8 |
| ρ_calc_g/cm^3^ | 1.259 |
| μ/mm^‑1^ | 0.683 |
| F(000) | 1200.0 |
| Crystal size/mm^3^ | 0.4 × 0.13 × 0.05 |
| Radiation | CuKα (λ = 1.54178) |
| 2Θ range for data collection/° | 19.39 to 149.538 |
| Index ranges | -9 ≤ h ≤ 10, -16 ≤ k ≤ 20, -27 ≤ l ≤ 27 |
| Reflections collected | 21466 |
| Independent reflections | 2986 [R_int_ = 0.0351, R_sigma_ = 0.0204] |
| Data/restraints/parameters | 2986/0/192 |
| Goodness-of-fit on F^2^ | 1.027 |
| Final R indexes [I>=2σ (I)] | R_1_ = 0.0374, wR_2_ = 0.0896 |
| Final R indexes [all data] | R_1_ = 0.0401, wR_2_ = 0.0912 |
| Largest diff. peak/hole / e Å^-3^ | 0.30/-0.18 |

| **Table 2 Fractional Atomic Coordinates (×10^4^) and Equivalent Isotropic Displacement Parameters (Å^2^×10^3^) for solve2_a_pl. U_eq_ is defined as 1/3 of of the trace of the orthogonalised U_IJ_ tensor.** | | | | |
| --- | --- | --- | --- | --- |
| **Atom** | ***x*** | ***y*** | ***z*** | **U(eq)** |
| O001 | 6667.3(8) | 1123.8(4) | 1276.2(3) | 16.39(17) |
| O004 | 5526.3(9) | 1319.8(5) | -193.3(3) | 21.87(19) |
| O006 | 5484.3(9) | 102.3(5) | 785.5(4) | 22.11(19) |
| C007 | 7291.6(12) | 2517.6(6) | 1553.3(5) | 15.4(2) |
| C00A | 6259.2(12) | 726.3(6) | 764.9(5) | 16.7(2) |
| C00C | 9458.6(12) | 1519.3(6) | 1286.2(5) | 16.1(2) |
| C00D | 7756.6(12) | 1811.5(6) | 1139.8(5) | 15.0(2) |
| C00G | 7428.7(13) | 1987.2(6) | 459.6(5) | 17.0(2) |
| C00I | 9688.4(13) | 910.7(7) | 1721.9(5) | 20.7(2) |
| C00L | 7847.4(13) | 3301.9(6) | 1420.1(5) | 19.7(2) |
| C00M | 6858.8(13) | 1172.5(6) | 196.1(5) | 17.1(2) |
| C00O | 7491.0(14) | 3951.2(7) | 1804.3(5) | 22.3(2) |
| C00P | 8157.9(14) | 656.9(7) | -115.1(5) | 24.0(2) |
| C00S | 11222.2(14) | 645.1(7) | 1871.1(5) | 24.8(3) |
| C00T | 6370.6(15) | 2397.0(7) | 2071.9(6) | 27.1(3) |
| C00U | 6560.4(14) | 3828.3(7) | 2318.7(5) | 24.4(3) |
| C00X | 10800.5(14) | 1876.2(7) | 1017.6(6) | 26.4(3) |
| C00Z | 12548.3(14) | 993.4(8) | 1595.7(6) | 27.7(3) |
| C012 | 12333.4(15) | 1611.9(9) | 1173.7(6) | 33.0(3) |
| C013 | 6001.6(17) | 3051.5(7) | 2451.3(6) | 32.1(3) |
| C015 | 8789.0(19) | 1041.1(9) | -700.7(6) | 38.2(3) |

| **Table 3 Anisotropic Displacement Parameters (Å^2^×10^3^) for solve2_a_pl. The Anisotropic displacement factor exponent takes the form: -2π^2^[h^2^a*^2^U_11_+2hka*b*U_12_+…].** | | | | | | |
| --- | --- | --- | --- | --- | --- | --- |
| **Atom** | **U_11_** | **U_22_** | **U_33_** | **U_23_** | **U_13_** | **U_12_** |
| O001 | 15.8(4) | 14.9(3) | 18.5(4) | 1.0(3) | -0.7(3) | -3.3(3) |
| O004 | 24.7(4) | 18.0(4) | 22.9(4) | 0.8(3) | -9.8(3) | -0.7(3) |
| O006 | 23.7(4) | 17.3(4) | 25.3(4) | 0.7(3) | -4.1(3) | -4.8(3) |
| C007 | 13.5(4) | 16.3(5) | 16.5(5) | 0.6(4) | -2.3(4) | 0.7(4) |
| C00A | 13.6(5) | 15.4(5) | 21.1(5) | -0.2(4) | -3.4(4) | 2.4(4) |
| C00C | 16.1(5) | 15.3(5) | 17.0(5) | -2.8(4) | -0.8(4) | 0.4(4) |
| C00D | 14.5(5) | 12.9(5) | 17.7(5) | 2.1(4) | 0.1(4) | -2.8(4) |
| C00G | 19.1(5) | 15.7(5) | 16.3(5) | 0.8(4) | -1.5(4) | 0.0(4) |
| C00I | 18.5(5) | 21.3(5) | 22.4(5) | 3.3(4) | -1.7(4) | -1.7(4) |
| C00L | 20.9(5) | 18.8(5) | 19.4(5) | 0.8(4) | 3.5(4) | -2.4(4) |
| C00M | 17.5(5) | 16.2(5) | 17.6(5) | 0.4(4) | -3.0(4) | 0.6(4) |
| C00O | 25.8(6) | 15.9(5) | 25.3(6) | 0.0(4) | 1.3(5) | -2.5(4) |
| C00P | 23.3(6) | 21.9(5) | 26.8(6) | -5.6(4) | 1.2(5) | 1.4(4) |
| C00S | 23.6(6) | 25.1(6) | 25.8(6) | 4.8(4) | -4.6(5) | 3.1(5) |
| C00T | 35.3(6) | 18.2(5) | 27.6(6) | 1.0(4) | 11.3(5) | -4.2(5) |
| C00U | 28.1(6) | 19.9(5) | 25.3(6) | -4.6(4) | 4.1(5) | 2.1(4) |
| C00X | 19.2(5) | 28.5(6) | 31.4(6) | 10.1(5) | 3.2(5) | 1.7(5) |
| C00Z | 17.3(5) | 33.7(6) | 32.1(6) | 2.2(5) | -1.5(5) | 6.6(5) |
| C012 | 16.5(6) | 40.6(7) | 42.0(7) | 12.4(6) | 5.8(5) | 1.7(5) |
| C013 | 42.6(7) | 26.1(6) | 27.5(6) | -1.9(5) | 17.8(5) | -3.3(5) |
| C015 | 44.1(8) | 36.6(7) | 34.0(7) | -9.4(6) | 16.9(6) | -5.2(6) |

| **Table 4 Bond Lengths for solve2_a_pl.** | | | | | | |
| --- | --- | --- | --- | --- | --- | --- |
| **Atom** | **Atom** | **Length/Å** |  | **Atom** | **Atom** | **Length/Å** |
| O001 | C00A | 1.3391(12) |  | C00G | C00M | 1.5259(14) |
| O001 | C00D | 1.4738(11) |  | C00I | C00S | 1.3871(16) |
| O004 | C00M | 1.4199(12) |  | C00L | C00O | 1.3862(15) |
| O006 | C00A | 1.2067(13) |  | C00M | C00P | 1.5307(15) |
| C007 | C00D | 1.5170(14) |  | C00O | C00U | 1.3827(16) |
| C007 | C00L | 1.3927(14) |  | C00P | C015 | 1.5224(17) |
| C007 | C00T | 1.3853(15) |  | C00S | C00Z | 1.3806(17) |
| C00A | C00M | 1.5281(14) |  | C00T | C013 | 1.3887(17) |
| C00C | C00D | 1.5286(14) |  | C00U | C013 | 1.3820(17) |
| C00C | C00I | 1.3917(15) |  | C00X | C012 | 1.3895(17) |
| C00C | C00X | 1.3904(15) |  | C00Z | C012 | 1.3813(17) |
| C00D | C00G | 1.5429(14) |  |  |  |  |

| **Table 5 Bond Angles for solve2_a_pl.** | | | | | | | | |
| --- | --- | --- | --- | --- | --- | --- | --- | --- |
| **Atom** | **Atom** | **Atom** | **Angle/˚** |  | **Atom** | **Atom** | **Atom** | **Angle/˚** |
| C00A | O001 | C00D | 110.82(8) |  | C00S | C00I | C00C | 120.77(10) |
| C00L | C007 | C00D | 119.28(9) |  | C00O | C00L | C007 | 120.33(10) |
| C00T | C007 | C00D | 121.58(9) |  | O004 | C00M | C00A | 108.45(8) |
| C00T | C007 | C00L | 119.11(10) |  | O004 | C00M | C00G | 108.83(8) |
| O001 | C00A | C00M | 111.68(8) |  | O004 | C00M | C00P | 112.13(8) |
| O006 | C00A | O001 | 120.89(10) |  | C00A | C00M | C00P | 109.40(9) |
| O006 | C00A | C00M | 127.42(9) |  | C00G | C00M | C00A | 102.01(8) |
| C00I | C00C | C00D | 119.63(9) |  | C00G | C00M | C00P | 115.39(9) |
| C00X | C00C | C00D | 121.62(9) |  | C00U | C00O | C00L | 120.30(10) |
| C00X | C00C | C00I | 118.67(10) |  | C015 | C00P | C00M | 113.13(10) |
| O001 | C00D | C007 | 107.52(8) |  | C00Z | C00S | C00I | 120.23(10) |
| O001 | C00D | C00C | 106.82(8) |  | C007 | C00T | C013 | 120.31(10) |
| O001 | C00D | C00G | 103.24(8) |  | C013 | C00U | C00O | 119.55(10) |
| C007 | C00D | C00C | 110.37(8) |  | C012 | C00X | C00C | 120.18(11) |
| C007 | C00D | C00G | 113.03(8) |  | C00S | C00Z | C012 | 119.39(11) |
| C00C | C00D | C00G | 115.14(8) |  | C00Z | C012 | C00X | 120.73(11) |
| C00M | C00G | C00D | 105.00(8) |  | C00U | C013 | C00T | 120.40(11) |

| **Table 6 Torsion Angles for solve2_a_pl.** | | | | | | | | | | |
| --- | --- | --- | --- | --- | --- | --- | --- | --- | --- | --- |
| **A** | **B** | **C** | **D** | **Angle/˚** |  | **A** | **B** | **C** | **D** | **Angle/˚** |
| O001 | C00A | C00M | O004 | -124.74(9) |  | C00D | C00G | C00M | O004 | 136.87(8) |
| O001 | C00A | C00M | C00G | -9.99(11) |  | C00D | C00G | C00M | C00A | 22.39(10) |
| O001 | C00A | C00M | C00P | 112.68(10) |  | C00D | C00G | C00M | C00P | -96.09(10) |
| O001 | C00D | C00G | C00M | -26.99(10) |  | C00G | C00M | C00P | C015 | -67.67(13) |
| O004 | C00M | C00P | C015 | 57.68(13) |  | C00I | C00C | C00D | O001 | -26.91(12) |
| O006 | C00A | C00M | O004 | 53.88(14) |  | C00I | C00C | C00D | C007 | 89.69(11) |
| O006 | C00A | C00M | C00G | 168.64(10) |  | C00I | C00C | C00D | C00G | -140.88(10) |
| O006 | C00A | C00M | C00P | -68.69(14) |  | C00I | C00C | C00X | C012 | 1.53(18) |
| C007 | C00D | C00G | C00M | -142.84(8) |  | C00I | C00S | C00Z | C012 | 0.43(19) |
| C007 | C00L | C00O | C00U | -0.95(17) |  | C00L | C007 | C00D | O001 | -164.18(9) |
| C007 | C00T | C013 | C00U | -0.6(2) |  | C00L | C007 | C00D | C00C | 79.66(11) |
| C00A | O001 | C00D | C007 | 141.44(8) |  | C00L | C007 | C00D | C00G | -50.91(12) |
| C00A | O001 | C00D | C00C | -100.09(9) |  | C00L | C007 | C00T | C013 | 0.33(18) |
| C00A | O001 | C00D | C00G | 21.73(10) |  | C00L | C00O | C00U | C013 | 0.71(18) |
| C00A | C00M | C00P | C015 | 178.04(10) |  | C00O | C00U | C013 | C00T | 0.0(2) |
| C00C | C00D | C00G | C00M | 89.05(10) |  | C00S | C00Z | C012 | C00X | -1.0(2) |
| C00C | C00I | C00S | C00Z | 1.17(18) |  | C00T | C007 | C00D | O001 | 17.99(13) |
| C00C | C00X | C012 | C00Z | 0.0(2) |  | C00T | C007 | C00D | C00C | -98.18(12) |
| C00D | O001 | C00A | O006 | 173.65(9) |  | C00T | C007 | C00D | C00G | 131.26(11) |
| C00D | O001 | C00A | C00M | -7.62(11) |  | C00T | C007 | C00L | C00O | 0.42(16) |
| C00D | C007 | C00L | C00O | -177.46(10) |  | C00X | C00C | C00D | O001 | 156.50(10) |
| C00D | C007 | C00T | C013 | 178.17(11) |  | C00X | C00C | C00D | C007 | -86.91(12) |
| C00D | C00C | C00I | C00S | -178.83(10) |  | C00X | C00C | C00D | C00G | 42.53(14) |
| C00D | C00C | C00X | C012 | 178.16(11) |  | C00X | C00C | C00I | C00S | -2.14(16) |

| **Table 7 Hydrogen Atom Coordinates (Å×10^4^) and Isotropic Displacement Parameters (Å^2^×10^3^) for solve2_a_pl.** | | | | |
| --- | --- | --- | --- | --- |
| **Atom** | ***x*** | ***y*** | ***z*** | **U(eq)** |
| H004 | 5219.9 | 875.79 | -348.2 | 33 |
| H00A | 6590.3 | 2412.8 | 413.33 | 20 |
| H00B | 8419.38 | 2174.51 | 252.17 | 20 |
| H00I | 8784.26 | 674.35 | 1919.38 | 25 |
| H00L | 8473.86 | 3392.47 | 1064.26 | 24 |
| H00O | 7887.84 | 4482.96 | 1713.79 | 27 |
| H00C | 9064.48 | 580.11 | 171.61 | 29 |
| H00D | 7711.04 | 109.57 | -209.78 | 29 |
| H00S | 11360.54 | 222.06 | 2163.84 | 30 |
| H00T | 5989.71 | 1864.12 | 2168.44 | 32 |
| H00U | 6306.84 | 4274.91 | 2579.18 | 29 |
| H00X | 10668.81 | 2301.92 | 726.57 | 32 |
| H00Z | 13598.99 | 809.48 | 1695.46 | 33 |
| H012 | 13243.73 | 1859.29 | 988.15 | 40 |
| H013 | 5361.3 | 2964.71 | 2804.15 | 38 |
| H01A | 9302.42 | 1566.18 | -606.51 | 57 |
| H01B | 7894.36 | 1131.02 | -983.5 | 57 |
| H01C | 9576.54 | 673.34 | -888.9 | 57 |

# **References**

(1) Pavlishchuk, V. V.; Addison, A. W. Conversion Constants for Redox Potentials Measured versus Different Reference Electrodes in Acetonitrile Solutions at 25 °C. Inorg. Chim. Acta. 2000, 298, 1, 97.

(2) 1. Espinoza, E. M.; Clark, J. A.; Soliman, J.; Derr, J. B.; Morales, M.; Vullev, V. I. Practical Aspects of Cyclic Voltammetry: How to Estimate Reduction Potentials When Irreversibility Prevails. J. Electrochem. Soc. 2019, 166, H3175.

(3) Dixon, I.; Collin, J.; Sauvage, J.; Flamigni, L.; Encinas, S.; Barigelletti, F. A family of luminescent coordination compounds: iridium(iii) polyimine complexes. *Chem. Soc. Rev****.***, 2000, 29, 385-391

(4) J. Burés, Variable Time Normalization Analysis: General Graphical Elucidation of Reaction Orders from Concentration Profiles. Angew. Chem. Int. Ed. 55, 16084–16087 (2016)

(5) Gao, Daxin. Heterocylcic Derivatives, Preparation Processes and Medical Uses Thereof. WO2012/71684.

(6) Zhang, X.; Cheng, X. Electrochemical Reductive Functionalization of Alkenes with Deuterochloroform as a One-Carbon Deuteration Block. *Org. Lett*. **2022**, 24, 47, 8645–8650.

(7) Sund, C.; Roue, N.; Lindsroem, S.; Dmitry, A.; Sahlberg, C.; Katarina, J. Non-Nucleotide Reverse Transcriptase Inhibitors. WO2005066131A1·2005-07-21.

(8) Camp, J.; Craig, D.; Funai, K.; White, A. Decarboxylative Claisen rearrangement reactions: synthesis and reactivity of alkylidene-substituted indolines. *Org. Biomol. Chem.*, **2011**,9, 7904-7912.

# **1H, 13C NMR and 19F NMR spectra of all compounds**

**3,3,3-trifluoro-2-oxopropanoic acid (1)**

**3,3,3-trifluoro-2-oxopropanoic acid-*d* (1-*d*)**

**1,2,3-trimethoxy-5-vinylbenzene (S2)**

**7-methoxy-1,2-dihydronaphthalene (S9)**

**tert-butyl-3-formyl-1H-indole-1-carboxylate (S10a)**

**tert-butyl-3-vinyl-1H-indole-1-carboxylate (S10)**

**9-methylene-9H-thioxanthene (S11)**

**9-methylene-9H-thioxanthene (S11)**

**3-hydroxy-5-(4-methoxyphenyl)-3-(trifluoromethyl)dihydrofuran-2(3H)-one (5)**

**3-hydroxy-3-(trifluoromethyl)-5-(3,4,5-trimethoxyphenyl)dihydrofuran-2(3H)-one (6)**

**3-hydroxy-5-(2-methoxyphenyl)-3-(trifluoromethyl)dihydrofuran-2(3H)-one (7)**

**4-(4-hydroxy-5-oxo-4-(trifluoromethyl)tetrahydrofuran-2-yl)phenyl acetate (8)**

**5-(4-bromophenyl)-3-hydroxy-3-(trifluoromethyl)dihydrofuran-2(3H)-one (9)**

**(3R,5S)-5-(4-bromophenyl)-3-hydroxy-3-(trifluoromethyl)dihydrofuran-2(3H)-one (9a-major)**

**(3S,5S)-5-(4-bromophenyl)-3-hydroxy-3-(trifluoromethyl)dihydrofuran-2(3H)-one (9b-minor)**

**5-(4-chlorophenyl)-3-hydroxy-3-(trifluoromethyl)dihydrofuran-2(3H)-one (10)**

**5-(4-fluorophenyl)-3-hydroxy-3-(trifluoromethyl)dihydrofuran-2(3H)-one (11)**

**5-(4-(tert-butyl)phenyl)-3-hydroxy-3-(trifluoromethyl)dihydrofuran-2(3H)-one (12)**

**3-hydroxy-3-(trifluoromethyl)-3a,4,5,9b-tetrahydronaphtho[1,2-b]furan-2(3H)-one (13)**

**tert-butyl 3-(4-hydroxy-5-oxo-4-(trifluoromethyl)tetrahydrofuran-2-yl)-1H-indole-1-carboxylate (14)**

**4-hydroxy-4-(trifluoromethyl)-3,4-dihydro-5H-spiro[furan-2,9'-thioxanthen]-5-one (15)**

**3-hydroxy-5,5-diphenyl-3-(trifluoromethyl)dihydrofuran-2(3H)-one (16)**

**3-hydroxy-5,5-diphenyldihydrofuran-2(3H)-one (17)**

**3-benzyl-3-hydroxy-5,5-diphenyldihydrofuran-2(3H)-one (18)**

**3-hydroxy-3-methyl-5,5-diphenyldihydrofuran-2(3H)-one (19)**

**3-ethyl-3-hydroxy-5,5-diphenyldihydrofuran-2(3H)-one (20)**

**ethyl 4-(formyloxy)-2-hydroxy-4-(4-methoxyphenyl)-2-(trifluoromethyl)butanoate (4a)**

**1,1,1-trifluoro-4-(4-methoxyphenyl)hept-6-en-2-one (21)**

**1,1,1-trifluoro-4-(3,4,5-trimethoxyphenyl)hept-6-en-2-one (22)**

**1,1,1-trifluoro-4-(2-methoxyphenyl)hept-6-en-2-one (23)**

**4-(7,7,7-trifluoro-6-oxohept-1-en-4-yl)phenyl acetate (24)**

**4-(4-bromophenyl)-1,1,1-trifluorohept-6-en-2-one (25)**

**4-(4-chlorophenyl)-1,1,1-trifluorohept-6-en-2-one (26)**

**1,1,1-trifluoro-4-(4-fluorophenyl)hept-6-en-2-one (27)**

**4-(4-(tert-butyl)phenyl)-1,1,1-trifluorohept-6-en-2-one (28)**

**3-(9-allyl-9H-thioxanthen-9-yl)-1,1,1-trifluoropropan-2-one (29)**

**1-(1-allyl-6-methoxy-1,2,3,4-tetrahydronaphthalen-2-yl)-2,2,2-trifluoroethan-1-one (30)**
